# Candidate gene screen for potential interaction partners and regulatory targets of the Hox gene *labial* in the spider *Parasteatoda tepidariorum*

**DOI:** 10.1007/s00427-020-00656-7

**Published:** 2020-02-08

**Authors:** Christoph Schomburg, Natascha Turetzek, Nikola-Michael Prpic

**Affiliations:** 1 Institut für Allgemeine Zoologie und Entwicklungsbiologie, AG Zoologie mit dem Schwerpunkt Molekulare Entwicklungsbiologie, Justus-Liebig-Universität Gießen, Heinrich-Buff-Ring 38, 35392 Gießen, Germany; 2 Ludwig-Maximilians-Universität München, Lehrstuhl für Evolutionäre Ökologie, Biozentrum II, Großhadernerstraße 2, 82152 Planegg-Martinsried, Germany

**Keywords:** Hox gene, *Labial*, Tritocerebral segment, Arthropod head evolution, Head segmentation

## Abstract

The Hox gene *labial* (*lab*) governs the formation of the tritocerebral head segment in insects and spiders. However, the morphology that results from *lab* action is very different in the two groups. In insects, the tritocerebral segment (intercalary segment) is reduced and lacks appendages, whereas in spiders the corresponding segment (pedipalpal segment) is a proper segment including a pair of appendages (pedipalps). It is likely that this difference between *lab* action in insects and spiders is mediated by regulatory targets or interacting partners of *lab*. However, only a few such genes are known in insects and none in spiders. We have conducted a candidate gene screen in the spider *Parasteatoda tepidariorum* using as candidates *Drosophila melanogaster* genes known to (potentially) interact with *lab* or to be expressed in the intercalary segment. We have studied 75 *P. tepidariorum* genes (including previously published and duplicated genes). Only 3 of these (*proboscipedia-A* (*pb-A*) and two paralogs of *extradenticle* (*exd*)) showed differential expression between leg and pedipalp. The low success rate points to a weakness of the candidate gene approach when it is applied to lineage specific organs. The spider pedipalp has no counterpart in insects, and therefore relying on insect data apparently cannot identify larger numbers of factors implicated in its specification and formation. We argue that in these cases a de novo approach to gene discovery might be superior to the candidate gene approach.

**Electronic supplementary material:**

The online version of this article (10.1007/s00427-020-00656-7) contains supplementary material, which is available to authorized users.

## Introduction

One of the main reasons for the evolutionary success of arthropods is the diversity of their appendages (Williams and Nagy 2001; Angelini and Kaufman 2005a). The serially homologous segments of these animals can bear appendages adapted for different purposes (reviewed in Prpic and Damen 2008; Jockusch 2017). While these structures share many developmental features, such as their regionalisation into proximal, median and distal domains, the evolution and development of different morphologies between species and among serially homologous appendages along the body axis of a species is not yet well understood (Angelini and Kaufman 2005a). One of the basic decisions for the development of particular appendage morphologies is the positional information derived from the expression of Hox genes along the anterio-posterior axis. For instance, the presence of *Antennapedia* (*Antp*) discriminates between antennal and walking leg fate in the vinegar fly *Drosophila melanogaster* (Casares and Mann 2001), and the absence of *Ultrabithorax* (*Ubx*) in the isopod crustacean *Parhyale hawaiensis* leads to the transformation of gnathopods into a second pair of maxillopods (Liubicich et al. 2009). Recent studies have begun to reveal some of the target genes that are regulated by the Hox genes to achieve specific morphologies. For example, the development of different leg morphologies in water striders is achieved by the interaction of *Ubx* and the gene encoding the Gamma interferon-inducible thiol reductase (Gilt) as a new target gene, which leads to the morphological changes needed for the jumping escape reflex (Armisén et al. 2015).

We study appendage development and appendage-type specification and diversification in the spider *Parasteatoda tepidariorum*. The spider body is divided into two parts, an anterior prosoma and a posterior opisthosoma. Spiders possess three types of prosomal appendages with different morphologies: the chelicerae, the pedipalps and the walking legs. Pedipalps and walking legs are morphologically very similar and also the expression of appendage patterning genes between pedipalps and walking legs resemble each other closely (reviewed in Pechmann et al. 2010). The main morphological difference between pedipalps and walking legs is that the pedipalps are usually smaller and shorter than the legs, and always lack one of the distal segments, the metatarsus. In addition, pedipalps possess specific modifications, i.e. the gnathendite at the base that is used for food processing, and the bulb at the tip (of the adult male pedipalp only) that is used as a copulatory organ.

The pedipalpal segment is the tritocerebral segment of the spiders and is therefore homologous to the insect intercalary segment (reviewed in Angelini and Kaufman 2005b). Previous work has shown that the positional information of the pedipalp segment and its pair of appendages in *P. tepidariorum* is provided by the expression of the Hox gene *labial-1* (*lab-1*) (Pechmann et al. 2015). RNA interference with the *lab-1* gene in *P. tepidariorum* leads to the loss of the pedipalps and an increase in cell death in the remaining tissue of the pedipalpal segment (Pechmann et al. 2015). The resulting reduced and appendage-less pedipalpal segment very much resembles the insect intercalary segment, not only morphologically, but also in terms of segmental gene expression (Pechmann et al. 2015). Thus, *lab-1* is required for normal pedipalp segment formation, and an insect-like reduced segment results from impaired function of *lab-1*. An obvious hypothesis is therefore that the normal insect intercalary segment is produced by an evolutionary loss of the function of *labial* orthologs in insects, but this is not the case. In fact, *labial* orthologs in insects are required for intercalary segment development as well, and loss of *labial* orthologs of several insects leads to increased cell death in the intercalary segment and adjacent head segments, very similar to the effect of *lab-1* loss in spiders (Merrill et al. 1989; Posnien and Bucher 2010; Schaeper et al. 2010). The general roles of *labial* orthologs are therefore very similar in insects and spiders, yet the segmental morphology orchestrated by this Hox gene in the two groups is very different. It is likely that divergent co-factors of *labial* orthologs or divergent regulatory targets downstream of *labial* orthologs are responsible for these differences, but in spiders no such factors have been identified yet. Therefore, we have compiled a list of candidate genes, which are either known or predicted interaction partners of *lab* in *D. melanogaster* or are expressed in the intercalary segment during *D. melanogaster* embryonic development. We have then systematically identified homologs of these genes in *P. tepidariorum* and have analysed their expression in embryos of *P. tepidariorum*. We reasoned that genes that are specifically expressed in the pedipalpal segment or that are expressed differentially between the pedipalps and the adjacent walking legs could represent possible interaction partners of *lab-1* and/or be involved in producing the specific morphology of the pedipalp appendages.

## Materials and methods

### Identification of candidate genes

A list of candidate genes was retrieved from The Drosophila Interactions Database (DroID) (Murali et al. 2011), containing all (known and putative) interaction partners of *lab*, as well as genes, which are expressed in the intercalary segment of *D. melanogaster* embryos, according to FlyBase (FB2014_03) (Thurmond et al. 2019).

Protein sequences of genes of interest from *D. melanogaster* were subjected to similarity search via BLAST (Altschul et al. 1990; Boratyn et al. 2013) against the *P. tepidariorum* transcriptome (Posnien et al. 2014). The BLAST parameters were: matrix: BLOSUM62, word size 6; cut-off E-value: 10-1, maximum of 50 hits per sequence. These hits were then reduced, so that only the sequence with the highest blast score per locus was kept. These sequences were subsequently used in a second BLAST-search against the RefSeq (O’Leary et al. 2016) and UniProt (UniProt Consortium T 2019) databases (“back-BLAST”). All sequences retained from the original BLAST search and the back-BLAST search were then aligned using clustalOmega (Sievers and Higgins 2014) and a phylogenetic tree was inferred using FastTree (Price et al. 2009) (results not shown). Since these trees contained a huge amount of genes, we decided to take from each tree the branch with our gene of interest and its neighbouring branch and infer a new maximum likelihood tree with these sequences (see Table S2 for the substitution model chosen by the program and amount of samples used in the analysis), using MrBayes (Ronquist and Huelsenbeck 2003; Ronquist et al. 2012).

### Animal cultivation and gene cloning

All animals used in this study originate from the Göttingen strain of *P. tepidariorum*. The embryos were staged after the embryonic staging table published by Mittmann and Wolff (2012). Total RNA was extracted from a mix of all embryonic stages using TRIzol® (Life Technologies, Carlsbad, CA, USA). cDNA was synthesized from total RNA with the SMARTer™ PCR cDNA Synthesis Kit (Clontech, Mountain View, CA, USA). Gene-specific cDNA fragments were amplified with primers (see Table S1) designed with Primer3 (Untergasser et al. 2012) and cloned into the pCR®II vector using the TA Cloning® Kit Dual Promoter (Invitrogen, Life Technologies, Carlsbad, CA, USA), with the exception of *exd-2*, which had previously been cloned with the same degenerate primers as *exd-1*.

### In situ staining and imaging

In situ hybridization and nuclear staining with SYTOX® Green were performed as described before (Prpic et al. 2008; Pechmann et al. 2009) with minor modifications. We used commercially available blocking reagent from Roche (Basel, Switzerland) (2% in PBST). Images were taken with a Leica M205 FA binocular (Leica Microsystems, Wetzlar, Germany) equipped with a QImaging MicroPublisher 5.0 RTV camera (QImaging, Surrey, Canada) and UV light. Images were corrected for colour values and brightness with Adobe Photoshop image processing software and arranged with Adobe Illustrator (both version CS6).

## Results

### Compilation of possible interaction partners of *labial*

**Fig. 1:**
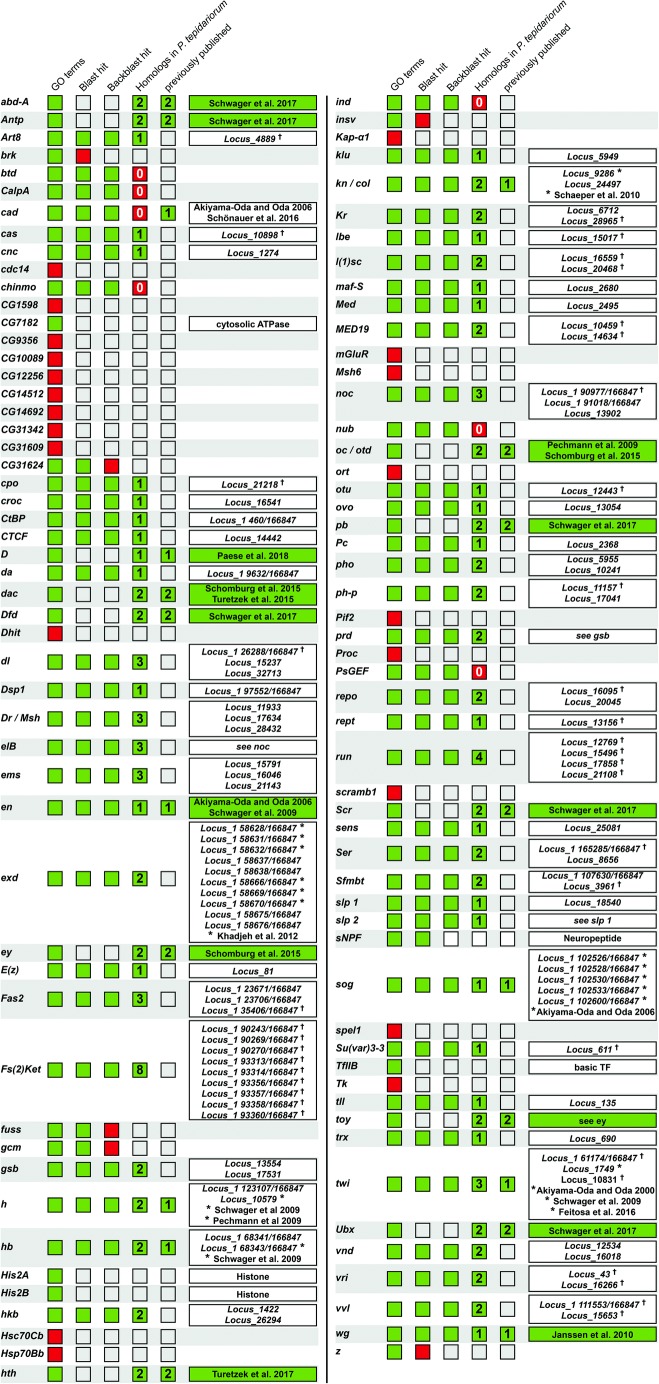
Overview of all genes on the candidate gene list. The first column gives the gene name according to *D. melanogaster* gene nomenclature. The second column shows whether the gene has been selected for further study after an initial GO terms analysis (green) or excluded from further study (red). The columns “Blast hit” and “Backblast hit” document whether the similarity analysis with BLAST was able to identify possible homologs in the *P. tepidariorum* transcriptome (green: significant BLAST hits were returned; red: no significant BLAST hits were returned; grey: no BLAST analysis was performed). The fifth column gives the number of identified homologs after phylogenetic analysis (green: at least one homolog is present in *P. tepidariorum*; red: no unambiguous homolog has been identified; grey: no phylogenetic analysis was performed). The sixth column shows whether the gene (or its duplicate(s), if any) has been studied previously (green: at least one paralog has been studied before, the numeral gives the number of paralogs studied previously). The last column gives additional information for selected genes. If a gene has been studied previously, key references are given; if only some of the identified paralogs have been studied previously, these are marked with an asterisk and the key publications are then given below the locus number. For identified homologs, the locus number in the *P. tepidariorum* transcriptome is also given. If more than one paralog have been identified, all locus numbers are given separately. Note that for *Female sterile (2) Ketel* (*Fs(2)Ket*) eight separate annotations were identified, but these overlap and therefore appear to comprise a single transcript (Fig. S8). A cross symbol next to the locus number indicates that no fragment of the corresponding cDNA could be cloned. For most of the genes a fragment of at least one paralog could be cloned with the exception of: *Arginine methyltransferase 8* (*Art8*; Fig. S10), *castor* (*cas*; Fig. S13), *couch potato* (*cpo*; Fig. S16), *Fs(2)Ket* (Fig. S30), *ladybird early* (*lbe*; Fig. S39), *lethal of scute* (*l(1)sc*; Fig. S40), *Mediator complex subunit 19* (*MED19*; Fig. S43), *ovarian tumor* (*otu*; Fig. S46), *reptin* (*rept*; Fig. S53), *runt* (*run*; Fig. S54), *Suppressor of variegation 3-3* (*Su(var)3-3*; Fig. S59), *vrille* (*vri*; Fig. S64), *ventral veins lacking* (*vvl*; Fig. S65)

We compiled altogether 105 genes in *D. melanogaster*, which were either annotated to be a potential interaction partner of *labial* (*lab*) in *D. melanogaster*, or which are expressed in the embryonic intercalary segment during *D. melanogaster* development (Fig. 1). The genes expressed in the embryonic intercalary segment are co-expressed with *lab*, but this does not mean that they are also necessarily interaction partners of *lab*. Therefore, we attempted to reduce the initially very high number of candidate genes. We excluded all genes that, based on their annotated GO terms (taken from FlyBase (FB2014_03) (Thurmond et al. 2019)), have a more general role in the cell, that may not be specific to the intercalary segment. We have instead focused on those genes for which the corresponding gene ontology (GO) terms suggested a function as transcription factor, or suggested a role in development, and/or gene expression. This first round of selection excluded the following 21 genes from further analysis: *cell division cycle 14* (*cdc14*), *CG1598*, *CG9356*, *CG10089*, *CG12256*, *CG14512*, *CG14692*, *CG31342*, *CG31609*, *Double hit* (*Dhit*), *Hsc70Cb*, *Heat-shock-protein-70Bb* (*Hsp70Bb*), *karyopherin α1* (*Kapα1*), *metabotropic Glutamate Receptor* (*mGluR*), *Msh6*, *ora transientless* (*ort*), *PFTAIRE-interacting factor 2* (*Pif2*), *Proctolin* (*Proc*), *scramblase 1* (*scramb1*), *spellchecker 1* (*spel1*) and *Tachykinin* (*Tk*). We further excluded the gene *CG7182*, since it is predicted to encode a cytosolic ATPase, and we also excluded the two histone-coding genes *His2A* and *His2B*, the short neuropeptide F precursor gene (*sNPF*), and *TfIIB* (coding for the general transcription initiator TFIIB), for being part of the basic cellular machinery. Of the remaining 79 genes, 12 had previously been studied extensively in *P. tepidariorum*, namely the Hox genes *abdominal-A* (*abd-A*)*, Antennapedia* (*Antp*)*, Deformed* (*Dfd*)*, proboscipedia* (*pb*)*, Sex combs reduced* (*Scr*)*, and Ubx* (Schwager et al. 2017), and the genes *dachshund* (*dac*) (Schomburg et al. 2015; Turetzek et al. 2015), *Dichaete* (*D*) (Bonatto Paese et al. 2018; Paese et al. 2018), *eyeless* (*ey*) (Schomburg et al. 2015), *homothorax* (*hth*) (Turetzek et al. 2017), *orthodenticle* (*otd*) (Pechmann et al. 2009; Schomburg et al. 2015) and *twin of eyeless* (*toy*) (Schomburg et al. 2015). Because a full analysis of these genes has already been published, we have not analysed these genes here again and have instead relied on those previous studies.

Three further genes, *brinker* (*brk*), *insensitive* (*insv*) and *zeste* (*z*), had no BLAST hit in the *P. tepidariorum* transcriptome, while another three genes (*CG31624*, *fussel* (*fuss*) and *glia cells missing* (*gcm*)) did not identify the original query sequence in the back-BLAST analysis. This left 61 genes for further analysis. For 8 of these genes, namely *caudal* (*cad*) (Akiyama-Oda and Oda 2006; Schönauer et al. 2016), *engrailed* (*en*) (Akiyama-Oda and Oda 2006; Schwager et al. 2009), *hairy* (*h*) (Pechmann et al. 2009; Schwager et al. 2009), *hunchback* (*hb*) (Schwager et al. 2009), *knot/collier* (*kn/col*) (Schaeper et al. 2010), *short gastrulation* (*sog*) (Akiyama-Oda and Oda 2006), *twist* (*twi*) (Akiyama-Oda and Oda 2003; Schwager et al. 2009; Feitosa et al. 2017) and *wingless* (*wg*) (Janssen et al. 2010), at least one homolog had previously been studied, but was published without comprehensive sequence analysis. For the genes *en* and *wg* we did not identify any additional similar sequences in the transcriptome, indicating that no additional paralogs of these genes are present in the transcriptome (Figs. S25 and S66). However, we identified additional sequences with similarity to the following genes: *h* (Fig. S32), *hb* (Fig. S33), *kn/col* (Fig. S37), *sog* (Fig. S58) and *twi* (Fig. S62). The phylogenetic analyses of the sequences similar to *h*, *kn*/*col* and *twi* suggest that these genes are each present as two or three paralogous genes in the *P. tepidariorum* genome (two paralogs of *h* and *kn/col*, three paralogs of *twi*). We have identified three sequences with similarity to *hb*, but only two of these appear to be proper homologs of *hb*, while the third sequence could not be placed unequivocally in the phylogenetic analysis (Fig. S33). The five sequences identified for *sog* perfectly match partially overlapping portions of the published sequence (Fig. S9). Therefore, we conclude that they do not represent individual paralogs, but are parts of the same transcript, which was artificially split up in the transcriptome assembly. We could not identify any homolog for *cad* in the transcriptome, despite the fact that a *P. tepidariorum* sequence for this gene has been identified previously (Akiyama-Oda and Oda 2006). We therefore performed a BLAST search with the published *P. tepidariorum cad* sequence against the transcriptome sequence and found 3 very short sequence fragments, which perfectly align with the query sequence, but are apparently too short to surface in the original BLAST search that was using the *D. melanogaster cad* sequence (Fig. S5).

For the following 6 genes on the candidate list, we were unable to identify an unambiguous homolog in the available *P. tepidariorum* transcriptome after phylogenetic analysis: *buttonhead* (*btd*) (Fig. S11), *CalpainA* (*CalpA*) (Fig. S12), *chinmo* (Fig. S15)*, intermediate neuroblasts defective* (*ind*) (Fig. S35), *nubbin* (*nub*) (Fig. S45) and *Protostome-specific GEF* (*PsGEF*) (Fig. S51).

The remaining 47 genes plus their spider-specific duplicates (if any), comprising altogether 78 candidate genes in *P. tepidariorum*, were then considered for further analysis. Not all of the 78 candidate genes could be cloned from cDNA derived from embryonic stages of *P. tepidariorum*: a full overview of the isolated sequences, as well as the unsuccessful molecular cloning attempts, is given in Fig. 1. We successfully cloned 43 genes (including paralogs) for further analysis within the scope of the in situ hybridisation screen. Together with the previously published genes plus their newly studied paralogs (32 genes), our screen for genes expressed specifically in the pedipalpal segment or expressed at least differentially in pedipalpal and leg segments included 75 genes in *P. tepidariorum* (summarized in Fig. 1).

### Expression of candidate genes: previously published genes with known or new paralogs

**Fig. 2:**
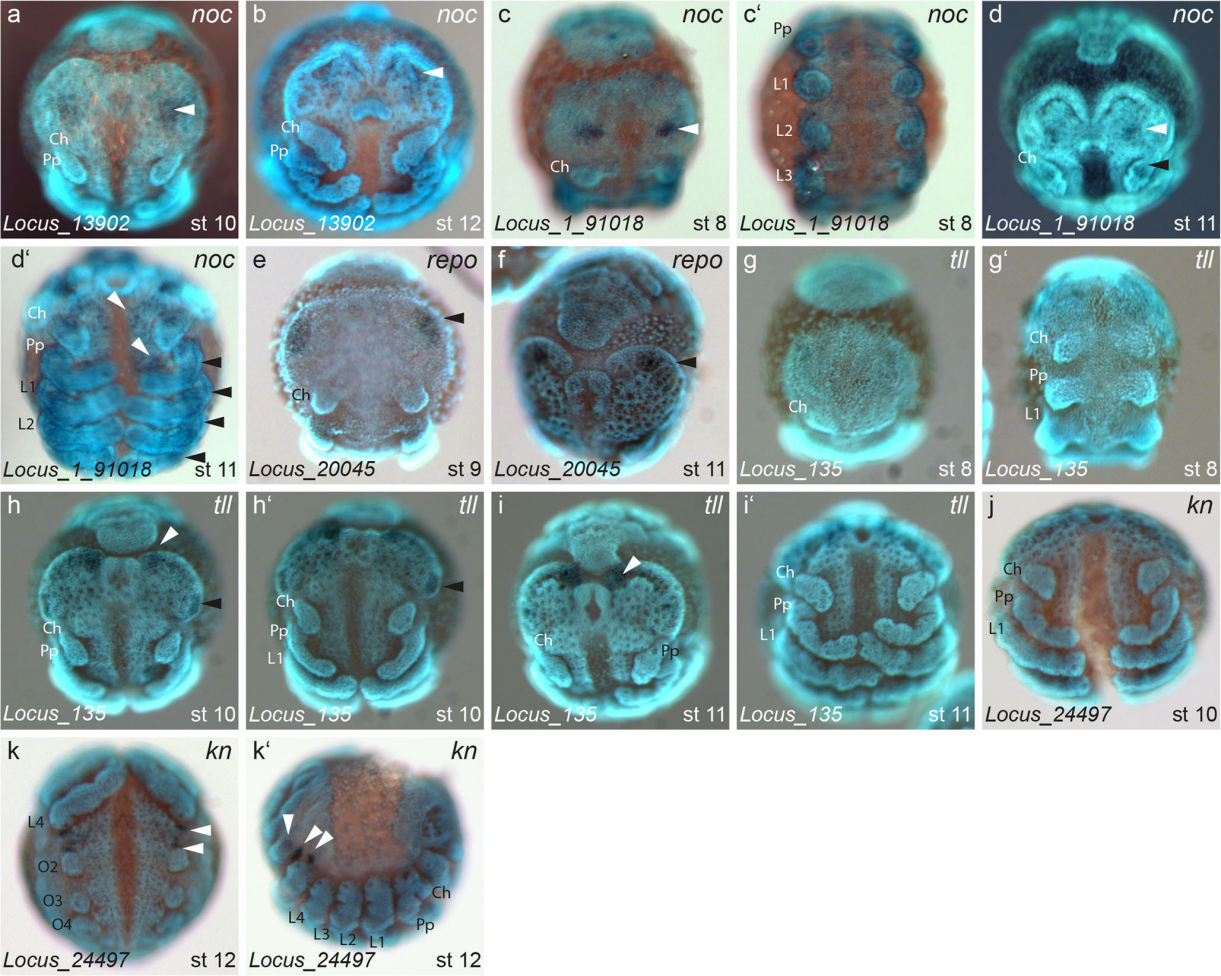
Genes with expression in the pre-cheliceral lobes. Expression of *noc/elB* locus_13902 (a, b), *noc/elB* locus_1_91018 (c, d′), *repo* locus 20045 (e, f), *tll* locus 135 (g–i′) and *kn /col* locus_24497. Arrowheads in a–d point to the expression domain in the head lobes. Black arrowhead in d points to expression in the chelicera. White arrowheads in d′ denote expression in proneural cell clusters, black arrowheads point to expression at the base of the appendages. Arrowheads in e, f denote expression in the head lobes. White arrowheads in h, i point to expression near the stomodeum. Black arrowheads in h, h′ point to expression in the head lobe. White arrowheads in k, k′ denote segmental expression. All embryos are shown with anterior to the top, except for k, which is a ventral aspect of the opisthosoma, and k′, which is a lateral aspect with anterior to the right. Abbreviations: Ch = chelicera, Pp = pedipalp, L = walking leg, O = opisthosomal segment

First, we used previously published expression data for the genes on the candidate gene list to assess possible differential expression of the genes in the pedipalps or the pedipalpal segment compared with the walking leg segments and their appendages. One paralog of the Hox gene *pb*, namely *pb-A*, is strongly expressed in the pedipalpal segment, but only weakly expressed in the walking leg segments (Schwager et al. 2017). The gene thus shows an expression pattern very similar to *lab-1* itself (Pechmann et al. 2015). The other Hox genes on the candidate gene list are not expressed in the pedipalpal segment at all, but are expressed in more posterior body segments (Schwager et al. 2017). This suggests that these Hox genes do not normally interact with *lab-1* in the pedipalpal segment, but of course this does not rule out the possibility that they may act as repressors of *lab-1* in other segments. Based on previously published accounts (see references in previous chapter and Fig. 1) of their expression pattern, the genes *dac*, *D*, *ey*, *hth*, *otd*, *toy*, *cad*, *en*, *sog* and *wg* (and their paralogs, if any) do not show apparent differential expression between the pedipalpal and the neighbouring walking leg bearing segments. The same is true for the genes *h*, *hb*, *kn/col* and *twi*, but in these four cases we have identified additional new putative paralogous genes in the genome of *P. tepidariorum* that have not been studied in the initial published accounts. The additional paralog of *h* (Locus_1 123107/166847) is expressed ubiquitously (Fig. S1). The newly identified paralog of *hb* (Locus_1 68341/166847) is expressed weakly in the pro-neural clusters during stages 10 and 11 (Fig. 5a, a′, b). The newly identified paralog of *kn/col* (Locus_24479) is only expressed at stage 12 as three spots in the segments L4 to O2 (white arrowheads in Fig. 2k, k′). It is not expressed in the pedipalpal segment or the head in general (Fig. 2j) and is therefore unlikely to interact with *lab-1* in the pedipalpal segment. The newly identified additional paralogs of *twi* (Locus_1 61174/166847 and Locus_10831) could not be studied further, because we failed to clone fragments of these transcripts from the cDNA preparations used for molecular cloning.

### Ubiquitously expressed genes or genes without detectable embryonic expression

Of the genes we have analysed, a relatively large proportion is expressed ubiquitously. The homologs of *cap ‘n’ collar* (*cnc*) (Locus_1274) (Fig. S14), *C-terminal binding protein* (*CtBP*) (Locus_1 460/166847) (Fig. S17), *CTCF* (Locus_14442) (Fig. S18), *daughterless* (*da*) (Locus_1 9632/166847) (Fig. S19), *dorsal* (*dl*) (Locus_15237, Locus_32713) (Fig. S21), *Drop* (*Dr*) (Locus_17634) (Fig. S21), *Dorsal switch protein 1* (*Dsp1*) (Locus_1 97552/166847) (Fig. S22), *empty spiracles* (*ems*) (Locus_21143) (Fig. S24), *Enhancer of zeste* (*E(z)*) (Locus_81) (Fig. S27), *Fasciclin-2* (*Fas2*) (Locus_23706) (Fig. S28), *hairy* (*h*) (Locus_1 123,107/166847) (Fig. S32), *huckebein* (*hkb*) (Locus_26294) (Fig. S34), *Medea* (*Med*) (Locus_2595) (Fig. S42), *Polycomb* (*Pc*) (Locus_2368) (Fig. S48), *polyhomeotic proximal* (*ph-p*) (Locus_17041) (Fig. S50), *pleiohomeotic* (*pho*) (Locus_5955, Locus_10241) (Fig. S49), *senseless* (*sens*) (Locus_25081) (Fig. S55), *Scm-related gene containing four mbt domains* (*Sfmbt*) (Locus_1 107630/166847) (Fig. S57), *Serrate* (*Ser*) (Locus_8656) (Fig. S56), *trithorax* (*trx*) (Locus_690) (Fig. S61) and *ventral nervechord defective* (*vnd*) (Locus_16018) (Fig. S63) show no separate expression domains in the embryos, but uniform and ubiquitous expression throughout the embryo (Figs. S1 and S2). Moreover, *Dr* (Locus_28432) (Fig. S21), *maf-S* (Locus_2680) (Fig. S41) and *pax3/7* (Locus_13554) (Fig. S31), a homolog of the *D. melanogaster* genes *paired* (*prd*) and *gooseberry* (*gsb*), did not display any detectable staining in the embryonic stages analysed (Figs. S1 and S2). Please note, however, that during the screening process no in situ hybridisation experiments have been repeated and, therefore, a cautionary note concerning the ubiquitously expressed and non-expressed genes is appropriate: all probes were subjected to the same quality checks (size control with electrophoresis and quantity control with spectrophotometry). However, we did not control for digoxigenin labelling efficiency during probe synthesis and we therefore cannot guarantee that every probe (especially in the cases where no expression was detected) worked properly.

### Expression in the pre-cheliceral lobes

Several of the analysed genes show prominent expression in the pre-cheliceral lobes. The two isolated paralogs of *no ocelli* (*noc*)/*elbow (elB)* (Locus_13902, Locus_1 91018/166847; Figs. S23 and S44) are expressed in two spots on either side of the head lobes (white arrowheads in Fig. 2a, b, c, d). While the sequence from Locus_13902 shows no further expression domains, its paralog is expressed ubiquitously in the limb buds at stage 8 (Fig. 2c′). At stage 11, additional expression is seen in the chelicerae (black arrowhead in Fig. 2d), as well as a segmental expression in several pro-neural clusters (white arrowheads in Fig. 2d′), and proximal expression domains in all appendages (black arrowheads in Fig. 2d′).

The homolog of *reversed polarity* (*repo*) (Locus_20045; Fig. S52) shows only one expression domain in an anterior-lateral position on either side of the head lobes (arrowheads in Fig. 2e, f).

The *tailless* (*tll*) *gene* with only one homolog in *P. tepidariorum* (Locus_135; Fig. S60) shows no expression at stage 8 (Fig. 2g, g′). From stage 10 on *tll* is expressed in one domain on either side of the stomodeum (white arrowheads in Fig. 2h, i). An additional expression domain is present at stage 10, at the lateral rim of the head lobes (black arrowheads in Fig. 2h, h′), which vanishes again by stage 11 (Fig. 2i, i′).

### Segmentally repeated expression

**Fig. 3:**
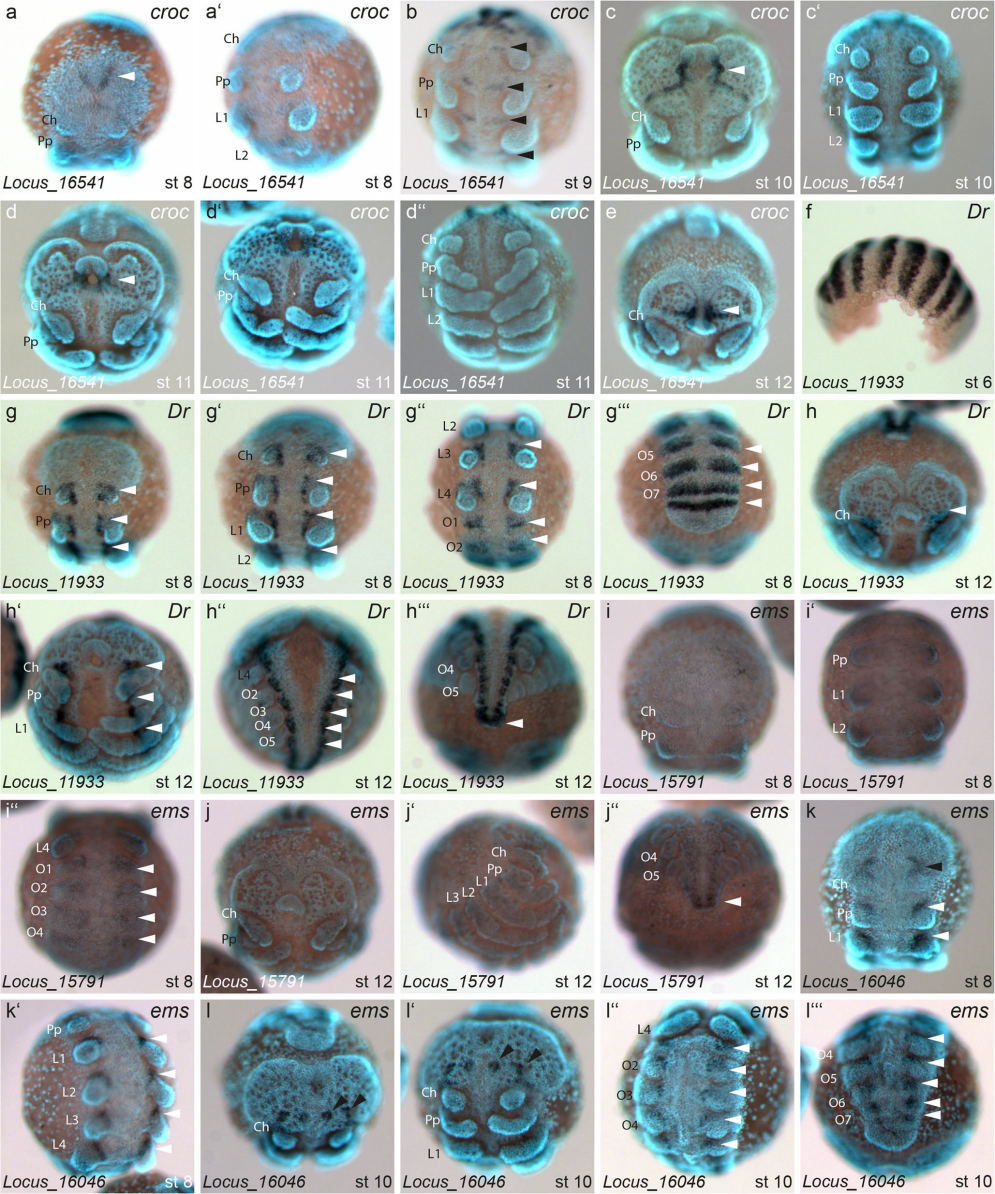
Genes with segmentally repeated expression patterns I. Expression of *croc* locus_16541 (a–e), *Drop* locus_11933 (f–h‴), *ems*_locus 15791 (i, j″), and *ems*_locus 16046. White arrowheads in a, c, d, e point to anterior expression. Black arrowheads in b point to segmentally repeated expression. White arrowheads in g–g‴ point to segmental patches (or stripes) of expression. White arrowheads in h–h‴ point to segmental expression along the entire body axis up to the very end of the germ band. White arrowheads in i″ and j″ denote segmental expression patches that include the posterior end of the germ band. White arrowheads in k, k′, l″, l‴ point to segmentally iterated patches of expression all along the body. All embryos are shown with anterior to the top, except for f, which is a lateral aspect with anterior to the left, and g‴, h″, h‴, i″, j″, l″, l‴, which are ventral aspects of the opisthosoma. Abbreviations: Ch = chelicera, Pp = pedipalp, L = walking leg, O = opisthosomal segment

We found several of the candidate genes to display a prominent segmental expression. The homolog of *crocodile* (*croc*) (Locus_16541; Fig. S29) is expressed in the pre-cheliceral lobes in a v-shape pattern and in later stages surrounds the stomodeum on either side with an additional later domain towards the edge of the head lobes (white arrowheads in Fig. 3a, c, d, e). We also observed a transient segmental expression at stage 9 (arrowheads in Fig. 3b), which vanishes again at stage 10 (Fig. 3c′, d″).

One of the identified homologs of *Dr* (Locus_11933; Fig. S21) is expressed in broad segmental stripes at stage 6 (Fig. 3f), which at stage 8 become a segmentally repeated pattern at the base of the appendages (arrowheads in Fig. 3g, g′, g″), while the striped pattern in the opisthosomal segments splits up along the midline in more anterior segments (arrowheads in Fig. 3g‴). At stage 12, *Dr* expression is present in every segment on the ventral side near the bases of the appendages and strongly in the former segment addition zone, as well as in the neural precursor groups along the body axis (arrowheads in Fig. 3h, h′, h″, h‴).

Two of the identified paralogs of *ems* (Fig. S25) also show segmental expression. Locus_15791 is weakly expressed at stage 8, in a pattern of segmentally repeated stripes in the opisthosomal segments (Fig. 3i″), and at stage 12 expression is only present in the most posterior tip of the germ band (Fig. 3j″). Locus_16046 is expressed in two v-shaped domains in the pre-cheliceral lobes at stage 8 (black arrowhead in Fig. 3k). This domain splits up into four distinct spots in later stages, which form a line across the head lobes (black arrowheads in Fig. 3l, l′). This transcript has additional expression domains in segmentally repeated stripes around the anterior-median quarter of the limb buds, which in later stages are located in several pro-neural clusters in every segment (white arrowheads in Fig. 3k, k′, l′, l″, l‴).

**Fig. 4:**
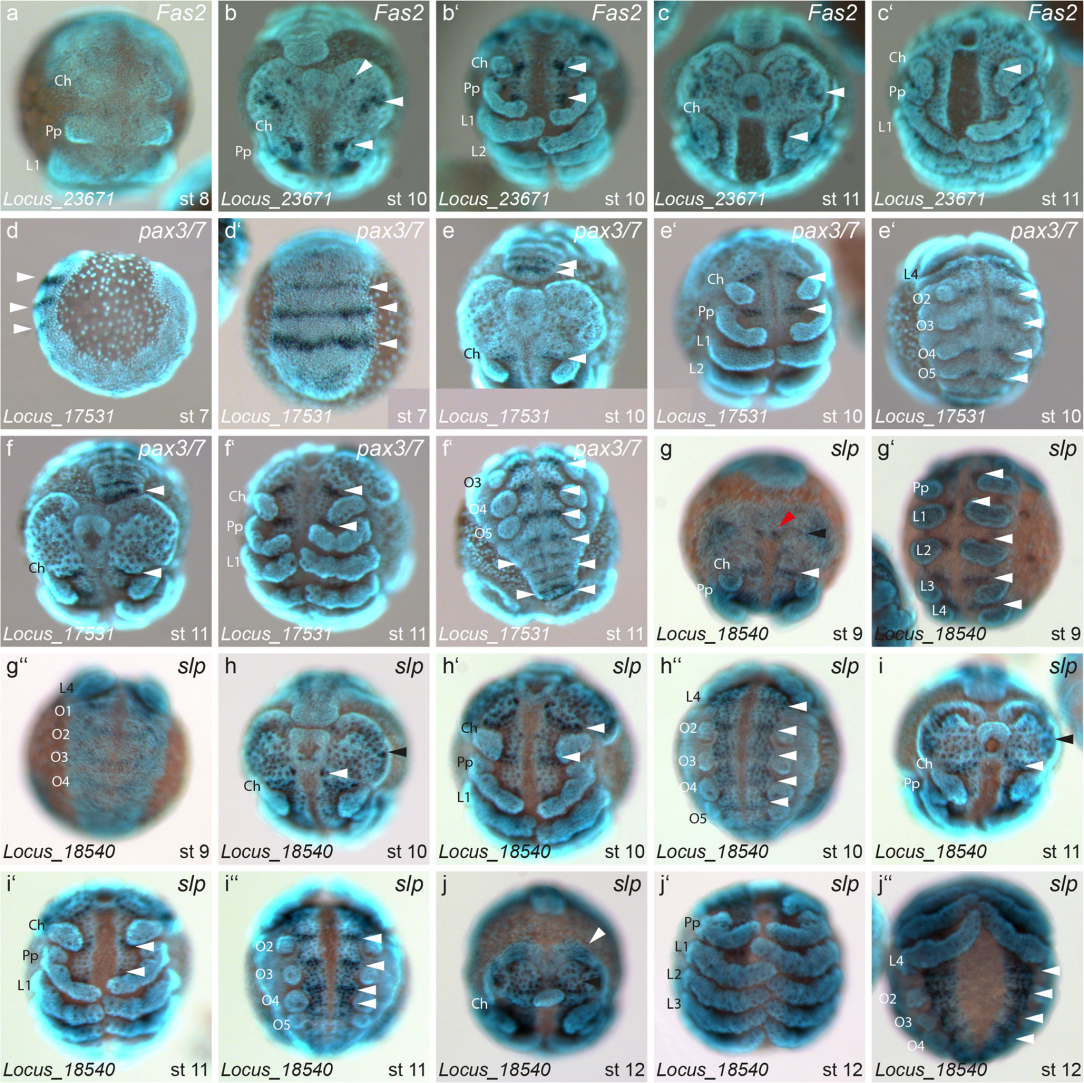
Genes with segmentally repeated expression patterns II. Expression of *Fas2* locus_23671 (a–c′), *pax3/7* locus_17531 (d–f″) and *slp* locus_18540 (g–j″). Arrowheads in a–c′ point to separate expression patches in the head segments. Arrowheads in d–f″ denote diverse segmental patches/stripes of the dynamic expression profile. White arrowheads in g–j″ point to segmental expression patches including a single patch in the head lobes. The black arrowheads in g, h, i, j point to expression near the lateral rim of the head lobes. The red arrowhead in g points to expression at the anterior end of the median sulcus near the stomodeum. All embryos are shown with anterior to the top, except for d, which is a lateral aspect with anterior to the right, and d′, e′, f″, g″, h″, i″, j″ which are ventral aspects of the opisthosoma. Abbreviations: Ch = chelicera, Pp = pedipalp, L = walking leg, O = opisthosomal segment

Locus_23671, which we identified as a homolog of *Fas2* (Fig. S28), shows no expression at stage 8 (Fig. 4a). In later stages, staining is present in two domains on either side of the head lobes (black arrowheads in Fig. 4b, c), as well as in segmentally repeated expression domains along the body, and also in the pro-neural clusters (white arrowheads in Fig. 4b, b′, c, c′).

The gene *pax3/7* (Locus_17531), which is a homolog of the *D. melanogaster* genes *prd* and *gsb* (Fig. S31), is expressed in segmentally repeated stripes throughout the embryonic stages analysed. At stage 6, it is expressed in the most posterior segments, emerging from the segment addition zone, with broader expression in the more recently formed segments (arrowheads in Fig. 4d, d′). In later stages, this gene is expressed in every segment (Fig. 4e, e′, e″, f, f′, f″).

Finally, the single *P. tepidariorum* homolog of the two *sloppy paired* genes in *D. melanogaster* (*slp1* and *slp2*; Fig. S29), is expressed in segmental stripes, which also include the neural precursor clusters along the body axis in the stages analysed (white arrowheads in Fig. 4g, g′, g″, h, h′, h″, i, i′, i″, j′, j″). Furthermore, we observed expression around the median sulcus at stage 8 (red arrowheads in Fig. 4g), two broader domains in the lateral portion of the head lobes (black arrowheads in Fig. 4g, h, i, j) and a domain in the anterior region of the pre-cheliceral lobes at stage 11 and 12 (Fig. 4i, j).

### Genes predominantly expressed in the nervous system

**Fig. 5:**
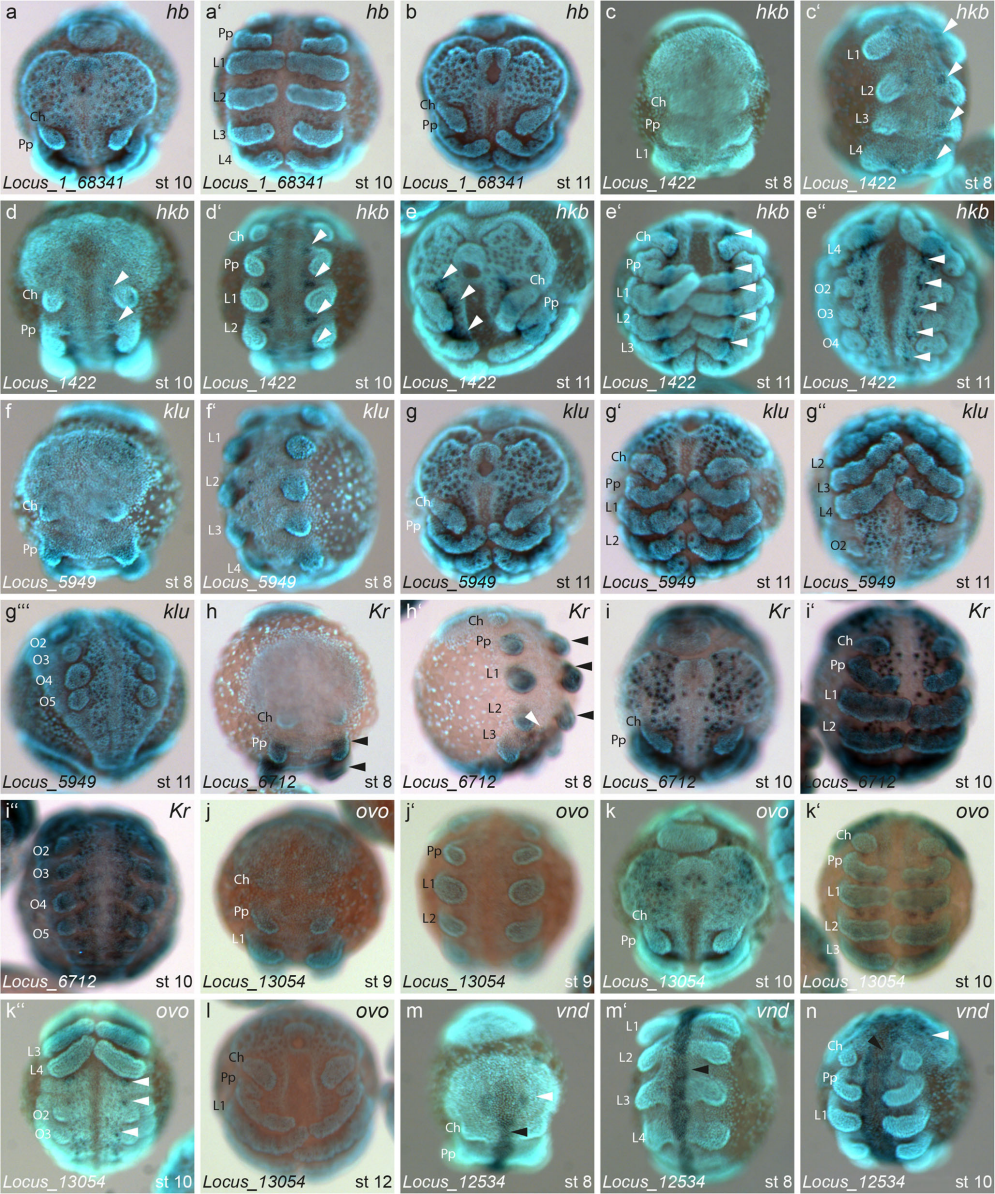
Genes with expression in the developing nervous system. Expression of *hb* locus_1_68341 (a, b), *hkb* locus_1422 (c–e″), *klu* locus_5949 (f, g‴), *Kr* locus_6712 (h–i″), *ovo* locus_13054 (j, k″) and *vnd* locus 12534 (m, n). White arrowheads in c′, d, d′, e, e′, e″ point to segmentally interated expression in the ventral nervous system. Black arrowheads in h, h′ denote ubiquitous expression in the appendages. White arrowhead in h′ points to the anterior border of posterior expression in the nervous system. White arrowheads in k″ denote expression in proneural clusters. White arrowheads in m, n point to expression in the developing brain. Black arrowheads in m-n denote expression along the ventral midline. All embryos are shown with anterior to the top, except for e″, g″, g‴, i″, k″ which are ventral aspects of the opisthosoma. Abbreviations: Ch = chelicera, Pp = pedipalp, L = walking leg, O = opisthosomal segment

Six of the analysed genes indicated a role in the development of the nervous system by their expression patterns. The newly identified paralog of *hb* (Locus_1 68,341/166847; Fig. S33) is expressed weakly in the pro-neural clusters during stages 10 and 11 (Fig. 5a, a′, b).

One of the paralogs of *hkb* (Locus_1422; Fig. S34) shows a segmental expression pattern, starting at stage 8 at the base of the appendages (arrowheads in Fig. 5c′, d, d′). At stage 11, this expression is then present in a subset of pro-neural clusters in each segment (arrowheads in Fig. 5e, e′, e″).

The homolog of *klumpfuss* (*klu*) (Locus_5949; Fig. S36) shows no expression at stage 8 (Fig. 5f, f′), but at stage 11 is weakly expressed in all pro-neural clusters of the embryo and the sensory organ precursors at the tips of the appendages (Fig. 5g, g′, g″, g‴).

The homolog of *Krueppel* (*Kr*) (Locus_6712; Fig. S38) is expressed ubiquitously in the appendages at stages 8 and 10 (black arrowheads in Fig. 5h, h′, i, i′). There is additional ubiquitous expression at stage 8 from the L3 segment towards the posterior end of the germ band (white arrowhead in Fig. 5h′). At stage 10, *Kr* also stains a large number of pro-neural clusters along the anterio-posterior axis (Fig. 5i, i′, i″).

At earlier stages, the homolog of *ovo* (Locus_13054; Fig. S47) showed no staining in the entire embryo, by stage 10 it was expressed in a subset of pro-neural clusters in the prosomal segments (Fig. 5k, k′), and a single cluster in each opisthosomal segment (white arrowheads in Fig. 5k″).

Finally, the homolog of *vnd* (Locus_12534; Fig. S63) appears as one spot of expression on either side in the centre of the pre-cheliceral lobes (white arrowheads in Fig. 5m, n), as well as a median stripe of expression along the ventral midline, which at stage 10 splits up on either side of the median sulcus (black arrowheads in Fig. 5m, m′, n).

### *Extradenticle* is differentially expressed between pedipalps and legs

**Fig. 6:**
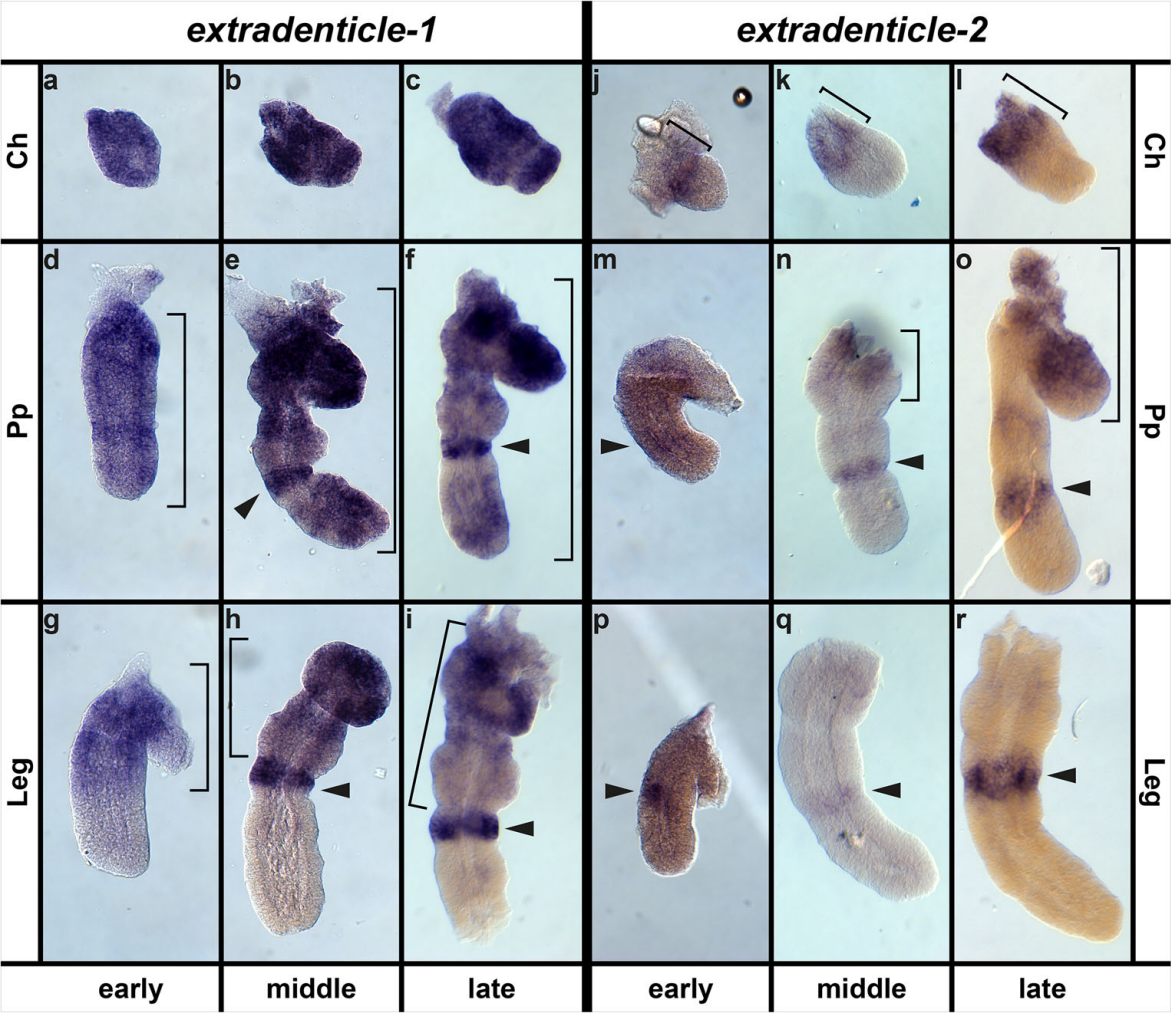
Expression of *exd-1* and *exd-2* in different appendage types. Expression of *exd-1* (**a**–**i**) and *exd-2* (**j**–**r**) in chelicera (top row), pedipalp (center row) and walking leg (bottom row) at early limb formation (first and fourth column), midterm (second and fifth row) and towards the end of inversion (third and sixth row). In all panels, brackets denote continuous extension of expression from the proximal end towards distal, and arrowheads point to locally elevated expression levels (“rings”). Abbreviations: Ch, chelicera; Pp, pedipalp

The gene *extradenticle* (*exd*) is present as 10 sequences in the transcriptome of *P. tepidariorum* (Fig. S26). However, these sequences map to only two predicted sequences (Figs. S6 and S7). We therefore assumed that there are two paralogous loci in the genome of *P. tepidariorum*, one of which is the previously published *exd-1* (Khadjeh et al. 2012) and the other (*exd-2*) we newly describe here. The *exd-1* gene is expressed at the base of the appendages throughout embryonic development (black arrowheads in Fig. S3a, a′, b, b′, c, c′, d, d′, Fig. 6a–i). Additionally, *exd-1* is expressed in the region of the labrum (white arrowheads in Fig. S3b, c), in a distinct ring in the pedipalps and legs (white arrowheads in Fig. S3b′, c′, d, black arrowheads in Fig. 6e, f, h, i) and in the opisthosomal appendages, as well as the more posterior opisthosomal segments (black arrowheads in Fig. S3c‴, d‴). We identified one significant difference in expression between the pedipalps and legs: while the legs show a medium-level expression in their proximal region only, the pedipalps show this expression from base to tip (red arrowheads in Fig. S3c′, d, d′, brackets in Fig. 6d–i).

The second *exd* gene, *exd-2*, is expressed at the base of the chelicerae (white arrowheads in Fig. S4a, a′, b, b′, brackets in Fig. 6j–l), and as a faint stripe along the embryo at the base of the appendages (Fig. S4a′), which becomes more prominent in the pedipalps as embryonic development progresses (compare pedipalpal expression in Fig. 6n, o to Fig. 6q, r). The *exd-2* gene is also expressed in a ring-shaped domain in the appendages (black arrowheads in Fig. S4b′, c′, d, d′, e′ and Fig. 6m–r), which is slightly fainter in the pedipalps than in the legs (red arrowhead in Fig. S4e′). Moreover, *exd-2* is expressed in a domain surrounding the pre-cheliceral lobes and the labrum at the edge of the non-neurogenic ectoderm. This domain continues along the lateral edges of the embryo, which represents future dorsal tissue (red and grey arrowheads in Fig. S4c, d, e).

## Discussion

### Basal cellular and developmental processes

The ubiquitous expression of many of the analysed *P. tepidariorum* genes supports a conserved function of these genes in basal cellular and developmental processes. In *D. melanogaster* and other animal species, many genes that are involved in chromatin remodelling or the maintenance of a certain transcriptional state (*CtBP*, *E(z)*, *Dsp1*, *Pc*, *ph-p*, *pho*, *sfmbt*, *trx*) (Courey and Jia 2001; Schuettengruber et al. 2007) are expressed ubiquitously. This is possible, because spatial specificity of their function is conferred by other means, e.g. local events of chromatin modification or spatially restricted binding partners (Cao et al. 2002; Czermin et al. 2002). Homologs of these genes are expressed ubiquitously in *P. tepidariorum* as well, suggestive of a conserved function of these genes in insects and spiders. The same is true for *CTCF*, which in *D. melanogaster* is an ubiquitous transcription factor that blocks enhancers (Moon et al. 2005; Mohan et al. 2007). The homolog of *Med* also shows ubiquitous expression in *P. tepidariorum* as well as in *D. melanogaster* (Tomancak et al. 2002, 2007). This is not unexpected for a gene that is involved in the transduction of TGF-beta-like signals (Das et al. 1998; Marquez et al. 2001) that rely on the correct spatio-temporal activation by factors from the outside rather than the specific expression of effectors themselves.

As in *D. melanogaster* (Cronmiller and Cummings 1993), *da* is expressed ubiquitously in *P. tepidariorum*. However, since the molecular mechanisms of the sex-determination system in spiders are largely unknown, no conclusion can be drawn yet on a conserved role of *da* in *P. tepidariorum*.

In *D. melanogaster*, *dl* establishes dorso-ventral patterning of the body axis through a nuclear concentration gradient of the protein (Anderson et al. 1985; Rushlow et al. 1989; Lall and Patel 2001) and is also involved in the immune response after injury (Lemaitre et al. 1995). The specification of the dorso-ventral axis in *P. tepidariorum* has been shown to be facilitated by the migration of cells of the cumulus, and thus by a mechanism apparently not dependent on *dorsal* function (Akiyama-Oda and Oda 2006).

### Head and brain development

The *D. melanogaster* paralogs of *noc/elB* are expressed in the region of the developing brain, and also appear to be involved in the development of appendages (Cheah et al. 1994; Weihe et al. 2004). In *P. tepidariorum*, the orthologs of *noc/elB* do not seem to be involved in the development of the appendages, but the patch of expression in the pre-cheliceral lobes suggests a conserved role in brain formation. The role of *tll* seems to be partly conserved with respect to the development of the optic system (Rudolph et al. 1997), while terminal structures (Casanova 1990; Moran and Jiménez 2006) appear not to be under its influence in *P. tepidariorum*, at least in those embryonic stages studied in the present work. This is consistent with previous findings that the terminal system is not entirely conserved among arthropods (Duncan et al. 2013).

Both, *cnc* and *kn/col* are involved in the segmentation of head structures in *D. melanogaster* (Veraksa et al. 2000; Peel 2004; Ntini and Wimmer 2011). Previous studies have shown that the expression patterns of both genes are not conserved among all arthropods. The eponymous cap and collar expression (distal “cap” and a “collar” around the stomodeum) is restricted to the Mandibulata clade, while expression in chelicerates and onychophorans is ubiquitous (Sharma et al. 2014; Janssen et al. 2011a; Hunnekuhl and Akam 2017; Janssen 2017). The head-specific expression of *kn/col* has been shown to be conserved in insects and myriapods, but not in other arthropod groups (Schaeper et al. 2010; Janssen et al. 2011b). The newly identified second *P. tepidariorum* homolog of *kn/col* is not expressed in the head either, thus supporting the previous conclusion that the role of *kn/col* in head segmentation is restricted to insects and myriapods.

The *P. tepidariorum* homolog of *repo* is expressed in patches in the pre-cheliceral lobes. This indicates a role in brain development, but provides no evidence for a conserved role in glial cell maintenance known from *D. melanogaster* (Halter et al. 1995).

### Aspects of segmentation

The anterior expression domains of *croc* are conserved among insects and myriapods (Birkan et al. 2011; Janssen et al. 2011a). Our results in *P. tepidariorum* indicate that the expression (and by inference also the role) of *croc* in head development is conserved in all arthropods. In *P. tepidariorum croc* also shows segmental expression in the mesoderm in the earlier stages, which correlates to mesodermal expression reported in *D. melanogaster* (Tomancak et al. 2002, 2007).

The segmentally iterated expression of *ems* paralogs in *P. tepidariorum* suggests that the role in the segmental development of the nervous system is conserved, but no paralog appears to be involved in identity specification of anterior segments as known in *D. melanogaster* (Schöck et al. 2000; Peel 2004).

A role of *pax3/7* in segmentation appears to be conserved in *P. tepidariorum*, although *pax3/7* is not expressed in a pair-rule pattern, like *prd* in *D. melanogaster*, but is expressed in segmentally repeated stripes. This difference has been shown for several pair-rule genes in spiders, especially members of the Pax group III (Damen et al. 2005; Schoppmeier and Damen 2005) and has been hypothesized to be the ancestral condition in arthropod segmentation (Peel et al. 2005). Based on the expression pattern in *P. tepidariorum*, *pax3/7* seems to have a function in the establishment of newly formed segments in the segment addition zone, and a role in the maintenance of mature segments, indicated by the segmental expression in later stages.

The *sloppy-paired* genes in *D. melanogaster* are pair-rule genes involved in trunk segmentation (Cadigan et al. 1994) and the specification of head segments (Grossniklaus et al. 1994). The segmental expression of *slp* in *P. tepidariorum* suggests that a function in segmentation might be conserved, although *slp* seems to specify one segment at a time and not in a pair-rule fashion.

### Similarities in neurogenesis between spiders and insects

Neuroblasts in *D. melanogaster* develop from a field of cells in the neuroectoderm, which expresses several proneural genes, such as *achaete*, *scute* and *lethal of scute* (Skeath and Thor 2003). While the establishment of neural precursors by *achaete-scute* genes and the subsequent lateral inhibition in these groups by Delta / Notch signalling is regarded to be generally conserved in spiders (Stollewerk 2002; Stollewerk et al. 2003), the mechanisms of separating neural precursors are different. While in *D. melanogaster* single neuroblasts delaminate from the neuro-ectoderm, in spiders there are several rounds of delamination of a larger group of cells, called neural progenitor groups (Stollewerk et al. 2003).

Interestingly, despite the mechanistical differences of conferring proneural identity between chelicerates and insects, *P. tepidariorum* and *D. melanogaster* share many of the genes to mark neural precursor cells in the ectoderm. Paralogs of *Dr*, *ems*, *Fas2*, *hb*, *hkb*, *klu*, *Kr*, *slp* and *vnd* are all expressed in subsets of neural precursor clusters in *P. tepidariorum*, similar to the expression in neuroblasts in *D. melanogaster* (Isshiki et al. 2001; Urbach and Technau 2003). Despite this superficial conservation, these genes do not mark separate neural precursors in the same area of their respective segments, but appear only to be expressed as marker genes to set aside the bulk of these cells from the rest of the neurogenic ectoderm. Notable differences concerning these genes include *tll*, which is expressed in *D. melanogaster* neuroblasts, but shows no neural expression in *P. tepidariorum*, and *ovo*, which marks neural precursors in *P. tepidariorum*, but not in *D. melanogaster*.

### *extradenticle* is differently expressed in pedipalps and legs

While *exd-1 is* expressed in the proximal part of the legs up to a prominent ring of expression in the median part, in the pedipalp the whole appendage expresses the transcript. Thus, *exd-1* is differentially expressed especially in the distal portion of legs and pedipalps. This expression therefore correlates with the lack of the metatarsus leg segment in the distal part of the pedipalp compared with the legs. The expression of *exd-2* at the base of the pedipalps in later embryonic stages correlates with the other morphological peculiarity of the pedipalps, the gnathendite. Only in the pedipalp *exd-2* is expressed in a proximal-ventral region, i.e. the area in which the gnathendite develops later. Since both *exd* paralogs show additional expression domains in the pedipalp, and because *exd* is implicated as a regulatory target of *lab* in *D. melanogaster*, the two *exd* paralogs are good candidates for genes that mediate the formation of major morphological peculiarities of the pedipalp under the control of *lab-1*.

## Conclusions

We have compiled a list of all genes from *D. melanogaster* that may be regulated by *lab* in the intercalary segment based on known interactions with *lab* and/or gene expression in the intercalary segment (the main expression locus of *lab*). We have then tested whether homologs of these genes from the spider *P. tepidariorum* are expressed in the pedipalp segment, which is the homolog of the insect intercalary segment and also the main expression locus of the spider *lab-1* gene. We have also tested whether the genes are differentially expressed in the pedipalpal segment and the adjacent leg bearing segments. Differential gene expression is a first indication that a gene might be involved in pedipalpal segment or pedipalp appendage specification and may be controlled by *lab-1*. After having compiled gene expression data from all previously published genes on the candidate gene list (32 genes, including duplicates), plus expression patterns of 43 newly studied genes (including duplicates), we find that three genes show differential expression between the intercalary and walking leg segments, namely *exd-1*, *exd-2* and *pb-A*. Thus, our screen for potential *lab* regulatory targets or co-factors in *P. tepidariorum* successfully identified 3 interesting candidates that now warrant further study, but the overall success rate is only 4% (3 genes out of 75 studied genes). This rather disappointingly low success rate points to a potential general problem of the candidate gene approach when it is applied to lineage-specific organs or evolutionary novelties. The spider pedipalp has no counterpart in *D. melanogaster* or other insects, and therefore relying on insect data as a basis for its potential genetic basis apparently cannot identify a larger number of conserved factors implicated in its specification and formation. Based on our results, we suggest that pedipalp-specific factors, and factors that are regulated by *lab-1* in *P. tepidariorum*, should be identified more reliably by de novo gene discovery approaches, for example transcriptome analyses of developing pedipalp tissue and differential transcriptomics of pedipalp tissue versus non-pedipalp tissue. In addition, co-factors and regulatory targets of *labial* orthologs specific to the spider model might also be identified more reliably via biochemical methods, such as ChIP-Seq and Co-IP pull-down.

## Electronic supplementary material

ESM 1 (XLSX 24 kb).

ESM 2 (XLSX 9 kb).

ESM 3 (PDF 9591 kb).

## References

[CR1] Akiyama-Oda Y, Oda H (2003). Early patterning of the spider embryo: a cluster of mesenchymal cells at the cumulus produces Dpp signals received by germ disc epithelial cells. Development.

[CR2] Akiyama-Oda Y, Oda H (2006). Axis specification in the spider embryo: dpp is required for radial-to-axial symmetry transformation and sog for ventral patterning. Development.

[CR3] Altschul SF, Gish W, Miller W, Myers EW, Lipman DJ (1990). Basic local alignment search tool. J Mol Biol.

[CR4] Anderson KV, Jurgens G, Nusslein-Volhard C (1985). Establishment of dorsal-ventral polarity in the *Drosophila* embryo. Genetic studies on the role of the *Toll* gene product. Cell.

[CR5] Angelini DR, Kaufman TC (2005). Insect appendages and comparative ontogenetics. Dev Biol.

[CR6] Angelini DR, Kaufman TC (2005). Comparative developmental genetics and the evolution of arthropod body plans. Annu Rev Genet.

[CR7] Armisén D, Nagui Refki P, Crumière AJJ, Viala S, Toubina W, Khila A (2015). Predator strike shapes antipredator phenotype through new genetic interactions in water striders. Nat Commun.

[CR8] Birkan M, Schaeper ND, Chipman AD (2011). Early patterning and blastodermal fate map of the head in the milkweed bug *Oncopeltus fasciatus*. Evol Dev.

[CR9] Bonatto Paese CL, Leite DJ, Schönauer A, McGregor AP, Russel S (2018). Duplication and expression of Sox genes in spiders. BMC Evol Biol.

[CR10] Boratyn GM, Camacho C, Cooper PS, Coulouris G, Fong A, Ma N, Madden TL, Matten WWT, McGinnis SD, Merezhuk Y, Raytselis Y, Sayers EW, Tao T, Ye J, Zaretskaya I (2013). BLAST: a more efficient report with usability improvements. Nucleic Acids Res.

[CR11] Cadigan KM, Grossniklaus U, Gehring WJ (1994). Localized expression of *sloppy paired* protein maintains the polarity of *Drosophila* parasegments. Genes Dev.

[CR12] Cao R, Wang L, Wang H, Xia L, Erdjument-Bromage H, Tempst P, Jones RS, Zhang Y (2002). Role of histone H3 lysine 27 methylation in Polycomb group silencing. Science.

[CR13] Casanova J (1990). Pattern formation under the control of the terminal system in the *Drosophila* embryo. Development.

[CR14] Casares F, Mann RS (2001). The ground state of the ventral appendage in *Drosophila*. Science.

[CR15] Cheah PY, Meng YB, Yang X, Kimbrell D, Ashburner M, Chia W (1994). The *Drosophila l(2)35Ba/nocA* gene encodes a putative Zn finger protein involved in the development of the embryonic brain and the adult ocellar structures. Mol Cell Biol.

[CR16] Courey AJ, Jia S (2001). Transcriptional repression: the long and the short of it. Genes Dev.

[CR17] Cronmiller C, Cummings CA (1993). The *daughterless* gene product in *Drosophila* is a nuclear protein that is broadly expressed throughout the organism during development. Mech Dev.

[CR18] Czermin B, Melfi R, McCabe D, Seitz V, Imhof A, Pirrotta V (2002). *Drosophila* enhancer of Zeste/ESC complexes have a histone H3 methyltransferase activity that marks chromosomal polycomb sites. Cell.

[CR19] Damen WGM, Janssen R, Prpic NM (2005). Pair rule gene orthologs in spider segmentation. Evol Dev.

[CR20] Das P, Maduzia LL, Wang H, Finelli AL, Cho S, Smith MM, Padgett RW (1998). The *Drosophila* gene *Medea* demonstrates the requirement for different classes of Smads in *dpp* signaling. Development.

[CR21] Duncan EJ, Benton MA, Dearden PK (2013). Canonical terminal patterning is an evolutionary novelty. Dev Biol.

[CR22] Feitosa NM, Pechmann M, Schwager EE, Tobias-Santos V, McGregor AP, Damen WG, Nunes da Fonseca R (2017). Molecular control of gut formation in the spider *Parasteatoda tepidariorum*. Genesis.

[CR23] Grossniklaus U, Cadigan KM, Gehring WJ (1994). Three maternal coordinate systems cooperate in the patterning of the *Drosophila* head. Development.

[CR24] Halter DA, Urban J, Rickert C, Ner SS, Ito K, Travers AA, Technau GM (1995). The homeobox gene *repo* is required for the differentiation and maintenance of glia function in the embryonic nervous system of *Drosophila melanogaster*. Development.

[CR25] Hunnekuhl VS, Akam M (2017). Formation and subdivision of the head field in the centipede *Strigamia maritima*, as revealed by the expression of head gap gene orthologues and hedgehog dynamics. Evodevo.

[CR26] Isshiki T, Pearson B, Holbrook S, Doe CQ (2001). *Drosophila* neuroblasts sequentially express transcription factors which specify the temporal identity of their neuronal progeny. Cell.

[CR27] Janssen R (2017). Comparative analysis of gene expression patterns in the arthropod labrum and the onychophoran frontal appendages, and its implications for the arthropod head problem. Evodevo.

[CR28] Janssen R, Le Gouar M, Pechmann M, Poulin F, Bolognesi R, Schwager EE, Hopfen C, Colbourne JK, Budd GE, Brown SJ, Prpic NM, Kosiol C, Vervoot M, Damen WGM, Balavoine G, McGregor AP (2010). Conservation, loss, and redeployment of Wnt ligands in protostomes: implications for understanding the evolution of segment formation. BMC Evol Biol.

[CR29] Janssen R, Budd GE, Damen WGM (2011a) Gene expression suggests conserved mechanisms patterning the heads of insects and myriapods. Dev Biol 357:64–72. 10.1016/j.ydbio.2011.05.670 10.1016/j.ydbio.2011.05.670 21658375

[CR30] Janssen R, Damen WG, Budd GE (2011b) Expression of *collier* in the premandibular segment of myriapods: support for the traditional Atelocerata concept or a case of convergence? BMC Evol Biol 11:50. 10.1186/1471-2148-11-50 10.1186/1471-2148-11-50 PMC3053236 21349177

[CR31] Jockusch EL (2017). Developmental and evolutionary perspectives on the origin and diversification of arthropod appendages. Integr Comp Biol.

[CR32] Khadjeh S, Turetzek N, Pechmann M, Schwager EE, Wimmer EA, Damen WGM, Prpic NM (2012). Divergent role of the Hox gene *Antennapedia* in spiders is responsible for the convergent evolution of abdominal limb repression. Proc Natl Acad Sci.

[CR33] Lall S, Patel N (2001). Conservation and divergence in molecular mechanisms of axis formation. Annu Rev Genet.

[CR34] Lemaitre B, Meister M, Govind S, Georgel P, Steward R, Reichart JM, Hoffmann JA (1995). Functional analysis and regulation of nuclear import of dorsal during the immune response in *Drosophila*. EMBO J.

[CR35] Liubicich DM, Serano JM, Pavlopoulos A, Kontarakis Z, Protas ME, Kwan E, Chatterjee S, Tran KD, Averof M, Patel NH (2009). Knockdown of *Parhyale Ultrabithorax* recapitulates evolutionary changes in crustacean appendage morphology. Proc Natl Acad Sci.

[CR36] Marquez RM, Singer MA, Takaesu NT, Waldrip WR, Kraytsberg Y, Newfeld SJ (2001). Transgenic analysis of the Smad family of TGF-β signal transducers in *Drosophila melanogaster* suggests new roles and new interactions between family members. Genetics.

[CR37] Merrill VKL, Diederich RJ, Turner FR, Kaufman TC (1989). A genetic and developmental analysis of mutations in *labial*, a gene necessary for proper head formation in *Drosophila melanogaster*. Dev Biol.

[CR38] Mittmann B, Wolff C (2012). Embryonic development and staging of the cobweb spider *Parasteatoda tepidariorum* C. L. Koch, 1841 (syn.: *Achaearanea tepidariorum*; Araneomorphae; Theridiidae). Dev Genes Evol.

[CR39] Mohan M, Bartkuhn M, Herold M, Philippen A, Heinl N, Bardenhagen I, Leers J, White RAH, Renkawitz-Pohl R, Saumweber H, Renkawitz R (2007). The *Drosophila* insulator proteins CTCF and CP190 link enhancer blocking to body patterning. EMBO J.

[CR40] Moon H, Filippova G, Loukinov D, Pugacheva E, Chen Q, Smith ST, Munhall A, Grewe B, Bartkuhn M, Arnold R, Burke LJ, Renkawitz-Pohl R, Ohlsson R, Zhou J, Renkawitz R, Lobanankov V (2005). CTCF is conserved from *Drosophila* to humans and confers enhancer blocking of the Fab-8 insulator. EMBO Rep.

[CR41] Moran E, Jiménez G (2006). The tailless nuclear receptor acts as a dedicated repressor in the early *Drosophila* embryo. Mol Cell Biol.

[CR42] Murali T, Pacifico S, Yu J, Guest S, Roberts GG, Finley RL (2011). DroID 2011: a comprehensive, integrated resource for protein, transcription factor, RNA and gene interactions for *Drosophila*. Nucleic Acids Res.

[CR43] Ntini E, Wimmer EA (2011). Second order regulator Collier directly controls intercalary-specific segment polarity gene expression. Dev Biol.

[CR44] O’Leary NA, Wright MW, Brister JR, Ciufo S, Haddad D, McVeigh R, Rajput B, Robbertse B, Smith-White B, Ako-Adjei AA, Bradetdin A, Bao Y, Blinkova O, Brover V, Chetvernin V, Choi J, Cox E, Ermolaeva O, Farrell CM, Goldfarb T, Gupta T, Haft D, Hatcher E, Hlvaina W, Joardar VS, Kodali VK, Li W, Maglott D, Masterson P, McGarvey KM, Murphy MR, O’Neill K, Pujar S, Rangwala SH, Rausch D, Riddick LD, Schoch C, Shkeda A, Storz SS, Sun H, Thibaud-Nissen F, Tolstoy I, Tully RE, Vatsan AR, Wallin C, Webb D, Wu W, Landrum MJ, Kimchi A, Tatusova T, DiCuccio M, Kitts P, Murphy TD, Pruitt KD (2016). Reference sequence (RefSeq) database at NCBI: current status, taxonomic expansion, and functional annotation. Nucleic Acids Res.

[CR45] Paese CLB, Schoenauer A, Leite DJ, Russel S, McGregor AP (2018). A SoxB gene acts as an anterior gap gene and regulates posterior segment addition in a spider. eLife.

[CR46] Pechmann M, McGregor AP, Schwager EE, Feitosa NM, Damen WGM (2009). Dynamic gene expression is required for anterior regionalization in a spider. Proc Natl Acad Sci.

[CR47] Pechmann M, Khadjeh S, Sprenger F, Prpic NM (2010). Patterning mechanisms and morphological diversity of spider appendages and their importance for spider evolution. Arthropod Struct Dev.

[CR48] Pechmann M, Schwager EE, Turetzek N, Prpic NM (2015). Regressive evolution of the arthropod tritocerebral segment linked to functional divergence of the Hox gene *labial*. Proc R Soc B Biol Sci.

[CR49] Peel A (2004). The evolution of arthropod segmentation mechanisms. BioEssays.

[CR50] Peel AD, Chipman AD, Akam M (2005). Arthropod segmentation: beyond the *Drosophila* paradigm. Nat Rev Genet.

[CR51] Posnien N, Bucher G (2010). Formation of the insect head involves lateral contribution of the intercalary segment, which depends on *Tc-labial* function. Dev Biol.

[CR52] Posnien N, Zeng V, Schwager EE, Pechmann M, Hilbrant M, Keefe JD, Damen WGM, Prpic NM, McGregor AP, Extavour CG (2014). A comprehensive reference transcriptome resource for the common house spider *Parasteatoda tepidariorum*. PLoS One.

[CR53] Price MN, Dehal PS, Arkin AP (2009). FastTree: computing large minimum evolution trees with profiles instead of a distance matrix. Mol Biol Evol.

[CR54] Prpic NM, Damen WGM (2008). Arthropod appendages: a prime example for the evolution of morphological diversity and innovation. Evolving pathways: key themes in evolutionary developmental biology.

[CR55] Prpic NM, Schoppmeier M, Damen WGM (2008) Collection and fixation of spider embryos. Cold Spring Harb Protoc 3. 10.1101/pdb.prot5067 10.1101/pdb.prot5067 21356698

[CR56] Ronquist F, Huelsenbeck JP (2003). MrBayes 3: Bayesian phylogenetic inference under mixed models. Bioinformatics.

[CR57] Ronquist F, Teslenko M, Van Der Mark P, Ayres DL, Darling A, Höhna S, Larget B, Liu L, Suchard MA, Huelsenbeck JP (2012). Mrbayes 3.2: efficient bayesian phylogenetic inference and model choice across a large model space. Syst Biol.

[CR58] Rudolph KM, Liaw G-J, Daniel A, Green P, Courey AJ, Hartenstein V, Lengyel JA (1997). Complex regulatory region mediating *tailless* expression in early embryonic patterning and brain development. Development.

[CR59] Rushlow CA, Han K, Manley JL, Levine M (1989). The graded distribution of the *dorsal* morphogen is initiated by selective nuclear transport in *Drosophila*. Cell.

[CR60] Schaeper ND, Pechmann M, Damen WGM, Prpic NM, Wimmer EA (2010). Evolutionary plasticity of *collier* function in head development of diverse arthropods. Dev Biol.

[CR61] Schöck F, Reischl J, Wimmer E, Taubert H, Purnell BA, Jäckle H (2000). Phenotypic suppression of *empty spiracles* is prevented by *buttonhead*. Nature.

[CR62] Schomburg C, Turetzek N, Schacht MI, Schneider J, Kirfel P, Prpic NM, Posnien N (2015). Molecular characterization and embryonic origin of the eyes in the common house spider *Parasteatoda tepidariorum*. Evodevo.

[CR63] Schönauer A, Paese CLB, Hilbrant M, Leite DJ, Schwager EE, Feitosa NM, Eibner C, Damen WGM, McGregor AP (2016). The Wnt and Delta-Notch signalling pathways interact to direct pair-rule gene expression via *caudal* during segment addition in the spider *Parasteatoda tepidariorum*. Development.

[CR64] Schoppmeier M, Damen WGM (2005). Expression of Pax group III genes suggests a single-segmental periodicity for opisthosomal segment patterning in the spider *Cupiennius salei*. Evol Dev.

[CR65] Schuettengruber B, Chourrout D, Vervoort M, Leblanc B, Cavalli G (2007). Genome regulation by polycomb and trithorax proteins. Cell.

[CR66] Schwager EE, Pechmann M, Feitosa M, McGregor AP, Damen WGM (2009). *hunchback* functions as a segmentation gene in the spider *Achaearanea tepidariorum*. Curr Biol.

[CR67] Schwager EE, Sharma PP, Clarke T, Leite DJ, Wierschin T, Pechmann M, Akiyama-Oda Y, Esposito L, Bechsgaard J, Bilde T, Buffry AD, Chao H, Dinh H, Doddapanei HV, Dugan S, Eibner C, Extavour CG, Funch P, Garb J, Gonzalez LB, Gonzalez VL, Griffiths-Jones S, Han Y, Hayashi C, Hilbrant M, Hughes DST, Janssen R, Lee SL, Maeso I, Murali SC, Muzny DM, Nunes da Fonseca R, Paese CLB, Qu J, Ronshaugen M, Schomburg C, Schönauer A, Stollewerk A, Torres-Oliva M, Turetzek N, Vanthournout B, Werren JH, Wolff C, Worley KC, Bucher G, Gibbs RA, Coddington J, Oda H, Stanke M, Ayoub NA, Prpic NM, Flot JF, Posnien N, Richards S, McGregor AP (2017). The house spider genome reveals an ancient whole-genome duplication during arachnid evolution. BMC Biol.

[CR68] Sharma PP, Gupta T, Schwager EE, Wheeler WC, Extavour CG (2014). Subdivision of arthropod *cap-n-collar* expression domains is restricted to Mandibulata. EvoDevo.

[CR69] Sievers F, Higgins DG (2014). Clustal Omega. Curr Protoc Bioinformatics.

[CR70] Skeath JB, Thor S (2003). Genetic control of *Drosophila* nerve cord development. Curr Opin Neurobiol.

[CR71] Stollewerk A (2002). Recruitment of cell groups through Delta/Notch signalling during spider neurogenesis. Development.

[CR72] Stollewerk A, Tautz D, Weller M (2003). Neurogenesis in the spider: new insights from comparative analysis of morphological processes and gene expression patterns. Arthropod Struct Dev.

[CR73] Thurmond J, Goodman JL, Strelets VB, Attrill H, Gramates LS, Marygold SJ, Matthews BB, Millburn G, Antonazzo G, Trovisco V, Kaufman TC, Calvi BR, FlyBase Consortium (2019). FlyBase 2.0: the next generation. Nucleic Acids Res.

[CR74] Tomancak P, Beaton A, Weiszmann R, Kwan E, Shu SQ, Lewis SE, Richards S, Ashburner M, Hartenstein V, Celniker SE, Rubin GM (2002). Systematic determination of patterns of gene expression during *Drosophila* embryogenesis. Genome Biol.

[CR75] Tomancak P, Berman BP, Beaton A, Weiszmann R, Kwan E, Hartenstein V, Celniker SE, Rubin GM (2007). Global analysis of patterns of gene expression during *Drosophila* embryogenesis. Genome Biol.

[CR76] Turetzek N, Pechmann M, Schomburg C, Schneider J, Prpic NM (2015). Neofunctionalization of a duplicate *dachshund* gene underlies the evolution of a novel leg segment in arachnids. Mol Biol Evol.

[CR77] Turetzek N, Khadjeh S, Schomburg C, Prpic NM (2017). Rapid diversification of *homothorax* expression patterns after gene duplication in spiders. BMC Evol Biol.

[CR78] UniProt Consortium T (2019). UniProt: a worldwide hub of protein knowledge. Nucleic Acids Res.

[CR79] Untergasser A, Cutcutache I, Koressaar T, Ye J, Faircloth BC, Remm M, Rozen SG (2012). Primer3-new capabilities and interfaces. Nucleic Acids Res.

[CR80] Urbach R, Technau GM (2003). Molecular markers for identified neuroblasts in the developing brain of *Drosophila*. Development.

[CR81] Veraksa A, McGinnis N, Li X, Mohler J, McGinnis W (2000). Cap ‘n’ collar B cooperates with a small Maf subunit to specify pharyngeal development and suppress deformed homeotic function in the *Drosophila* head. Development.

[CR82] Weihe U, Dorfman R, Wernet MF, Cohen SM, Milán M (2004). Proximodistal subdivision of *Drosophila* legs and wings: the *elbow-no ocelli* gene complex. Development.

[CR83] Williams TA, Nagy LM (2001) Developmental modularity and the evolutionary diversification of arthropod limbs. J Exp Zool 291:241–257. 10.1002/jez.1101 10.1002/jez.1101 11598913

