# Supplementary material for: Candidate gene screen for potential interaction partners and regulatory targets of the Hox gene labial in the spider Parasteatoda tepidariorum
Source: Dev Genes Evol. 2020 Feb 8;230(2):105–20. doi: 10.1007/s00427-020-00656-7 (PMC7128011; doi:10.1007/s00427-020-00656-7)
Supplement: Supplementary file 3 — (PDF 9591 kb). [file 427_2020_656_MOESM3_ESM.pdf]

ESM 3: SUPPLEMENTARY FIGURES AND LEGENDS

Expression data:

S1 – Ubiquitous expression/no expression p.02
S2 – Ubiquitous expression/no expression p.03
S3 – Expression exd-1, whole mount p.04
S4 – Expression exd-2, whole mount p.05

Alignments:

S5 – caudal p.06
S6 – exd-1 p.07
S7 – exd-2 p.08
S8 – Fs(2)Ket p.09
S9 – sog p.10

Phylogenetic trees:

S10 – Art8 p.11
S11 – btd p.12
S12 – CalpA p.13
S13 – cas p.14
S14 – chinmo p.15
S15 – cnc p.16
S16 – cpo p.17
S17 – CtBP p.18
S18 – CTCF p.19
S19 – da p.20
S20 – dl p.21
S21 – Dr / Msh p.22
S22 – Dsp1 p.23
S23 – elB p.24
S24 – ems p.25
S25 – en p.26
S26 – exd p.27
S27 – E(z) p.28
S28 – Fas2 p.29
S29 – FOX (croc, slp) p.30
S30 – Fs(2)Ket p.31
S31 – gsb / prd p.32
S32 – h p.33
S33 – hb p.34
S34 – hkb p.35
S35 – ind p.36
S36 – klu p.37
S37 – kn /col p.38
S38 – Kr p.39
S39 – lbe p.40
S40 – l(1)sc p.41
S41 – maf-S p.42
S42 – Med p.43
S43 – MED19 p.44
S44 – noc p.45
S45 – nub p.46
S46 – otu p.47
S47 – ovo p.48
S48 – Pc p.49
S49 – pho p.50
S50 – php p.51
S51 – PsGEF p.52
S52 – repo p.53
S53 – rept p.54
S54 – run p.55
S55 – sens p.56
S56 – Ser p.57
S57 – Sfmbt p.58
S58 – sog p.59
S59 – Su(var)3-3 p.60
S60 – tll p.61
S61 – trx p.62
S62 – twi p.63
S63 – vnd p.64
S64 – vri p.65
S65 – vvl p.66
S66 – wg p.67

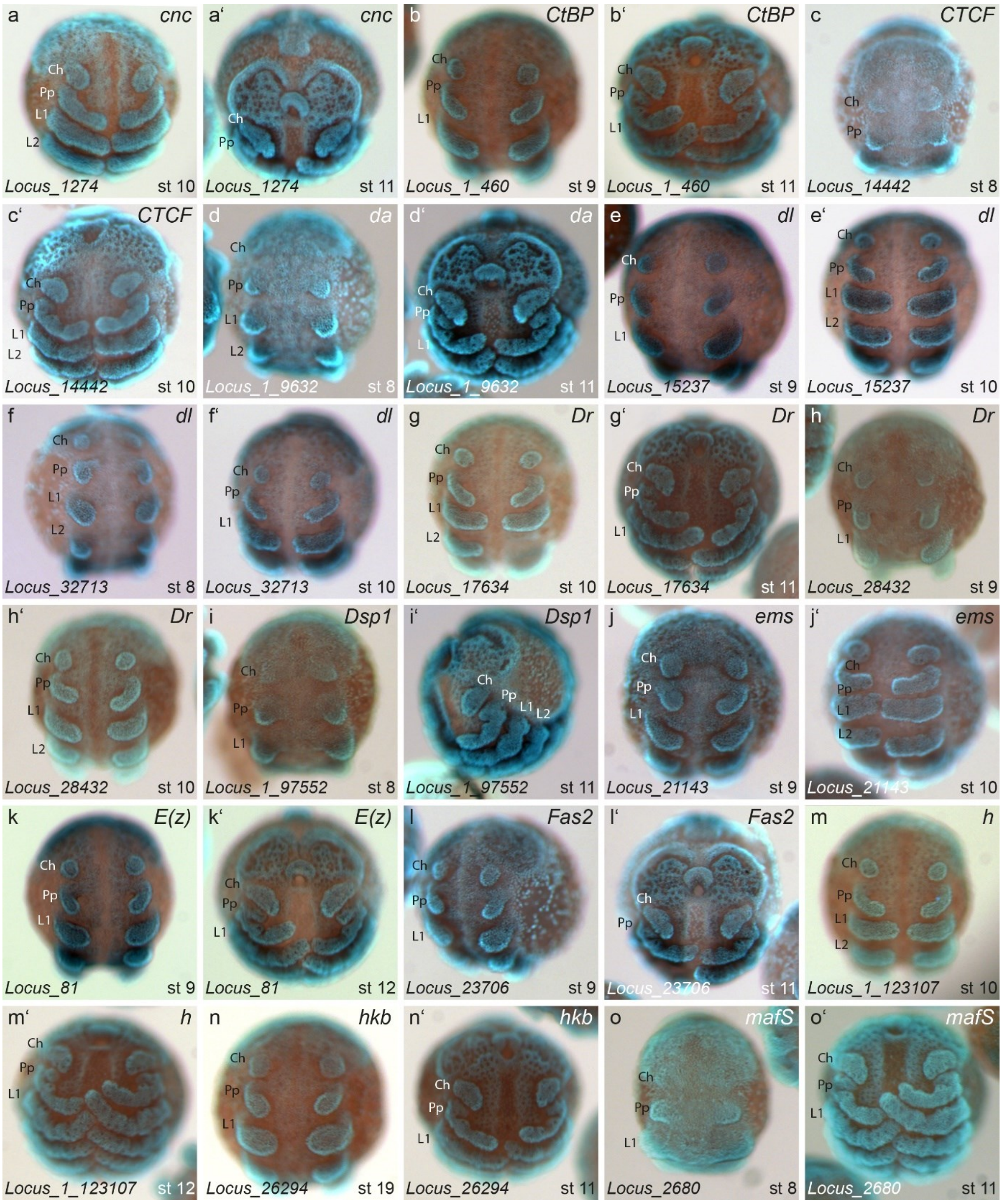

Figure S1. Ubiquitous expression / no expression I. Gene names are shown in upper right-hand corner, corresponding transcriptomic locus shown in lower left-hand corner, stage indicated in lower right-hand corner. All embryos are shown with anterior to the top, except for i’, which is a half-lateral view with focus on the pedipalp. Abbreviations: Ch = chelicera, Pp = pedipalp, L = walking leg, O = opisthosomal segment.

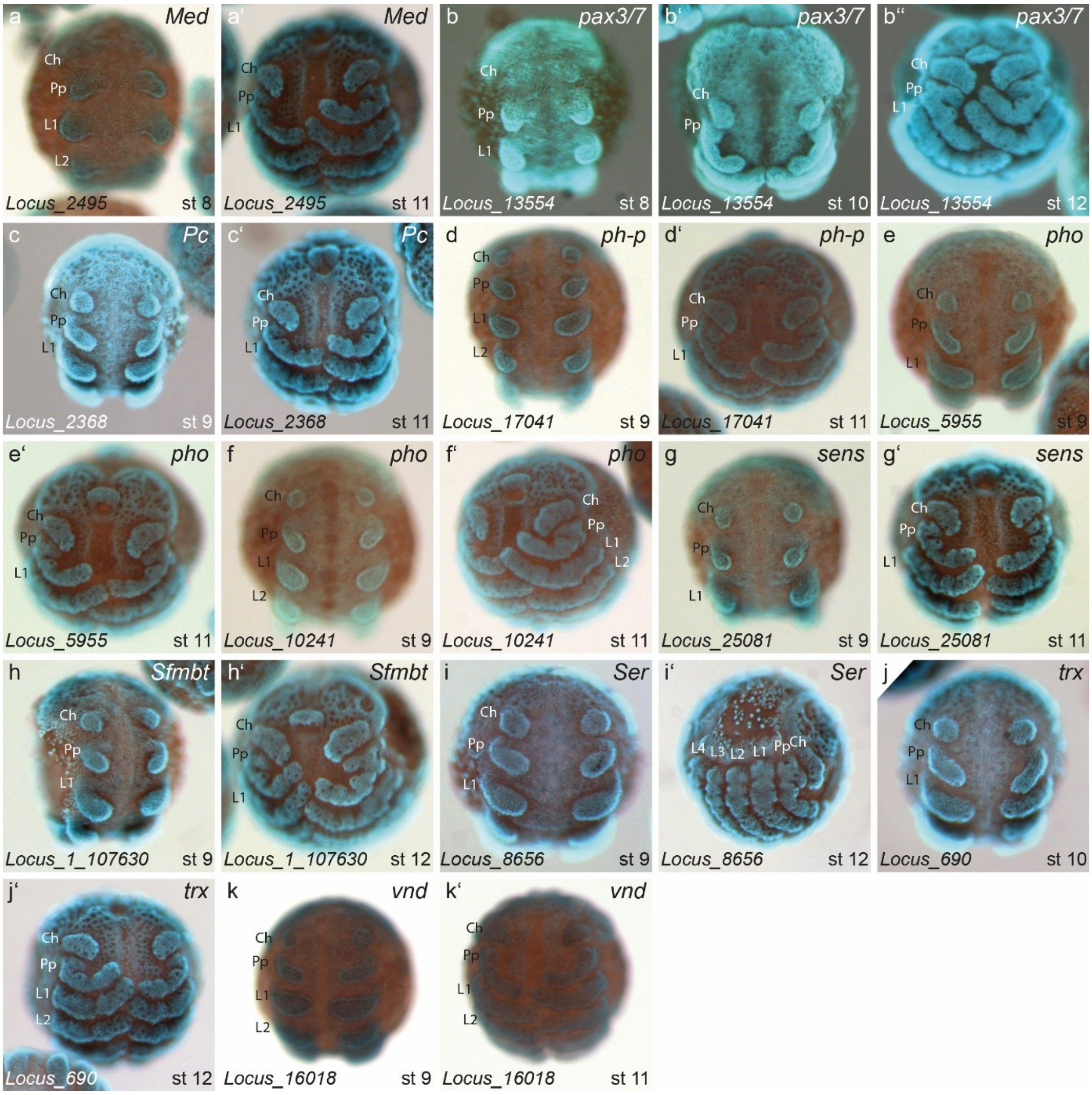

Figure S2. Ubiquitous expression / no expression II. Gene names are shown in upper right-hand corner, corresponding transcriptomic locus shown in lower left-hand corner, stage indicated in lower right-hand corner. All embryos are shown with anterior to the top, except for for i’, which is a lateral view with anterior to the right. Abbreviations: Ch = chelicera, Pp = pedipalp, L = walking leg, O = opisthosomal segment.

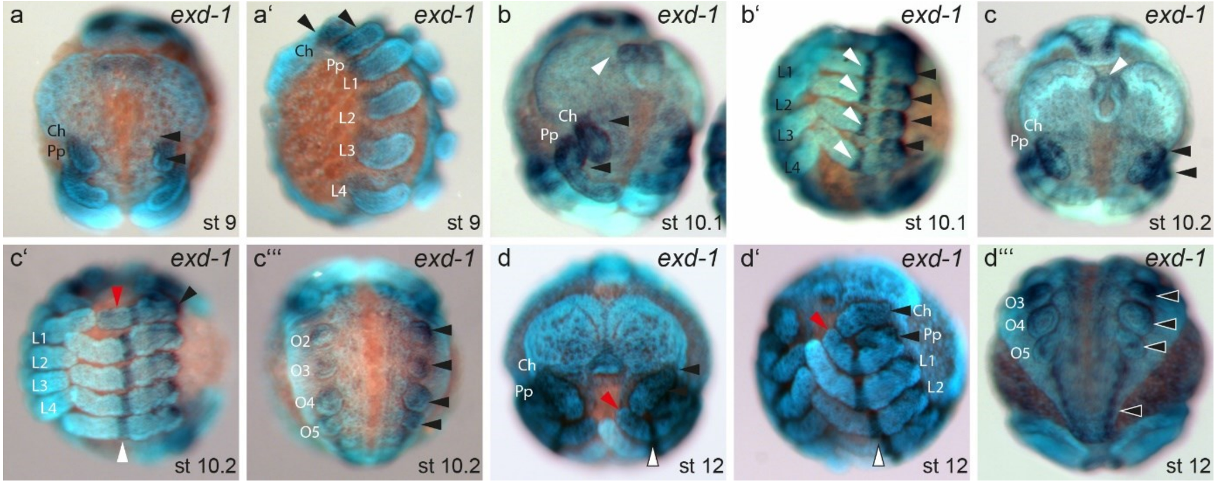

Figure S3. Expression of *exd-1*. Gene name is shown in upper right-hand corner, stage indicated in lower right-hand corner. All embryos are shown with anterior to the top, except for a', b, and d', which are half-lateral views with focus on the appendages. Black arrowheads point to expression at the base of the appendages, including the opisthosomal limb buds, and expression at a comparable (serially homologous?) location in the remaining opisthosomal segments. The white arrowheads in b, c point to expression in the labrum. The white arrowheads in b', c' denotes the medial expression ring in the legs and pedipalps. The red arrowhead points to expression in the distal portion of the pedipalp. Abbreviations: Ch = chelicera, Pp = pedipalp, L = walking leg, O = opisthosomal segment.

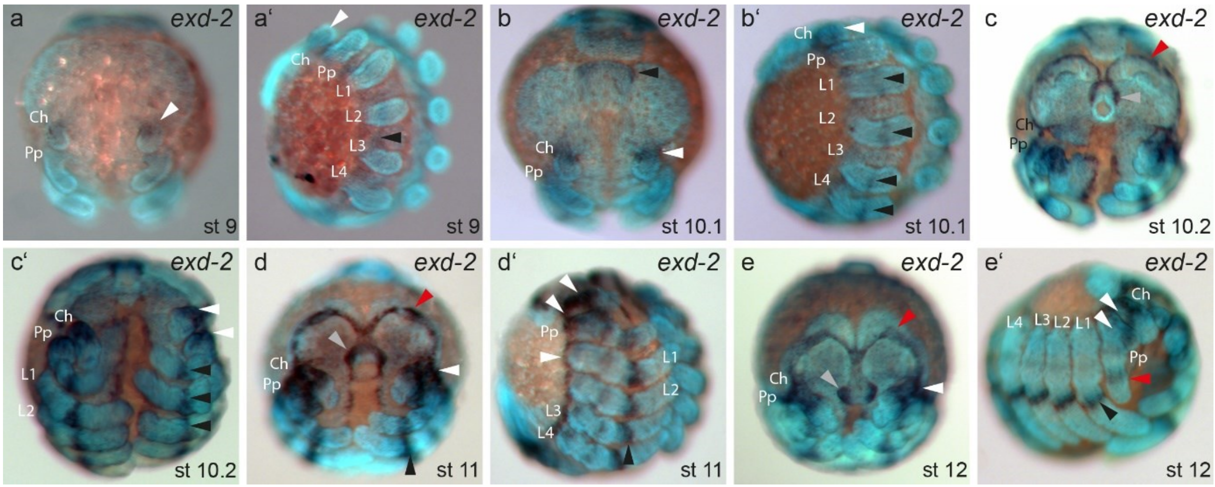

Figure S4. Expression of *exd-2*. Gene name is shown in upper right-hand corner, stage indicated in lower right-hand corner. All embryos are shown with anterior to the top, except for b’, d’, and e’, which are half-lateral views with focus on the appendages. The white arrowheads point to expression in the chelicera and/or the pedipalp base. The black arrowhead in a' denotes faint expression near the base of the appendages. Black arrowheads in b', c', d, d', e' point to the ring-shaped expression domain in the legs and pedipalps. The grey and red arrowheads in c, d, e denote expression at the anterior rim of the germ band anterior to the labrum (grey arrowheads) and the head lobes (red arrowheads). The red arrowhead in e' points to fainter expression ring in the pedipalps. Abbreviations: Ch = chelicera, Pp = pedipalp, L = walking leg, O = opisthosomal segment.

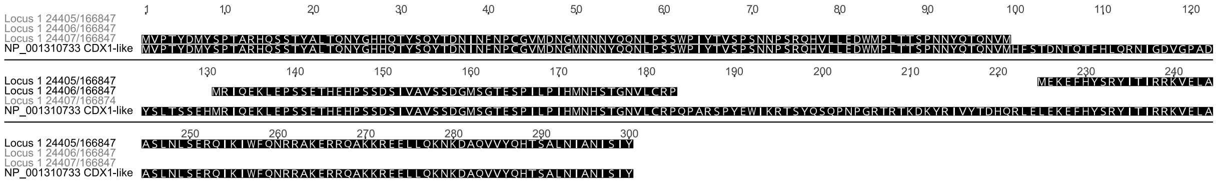

Figure S5. Alignment of the three short translated transcriptomic sequences found for *caudal* with the published Caudal sequence.

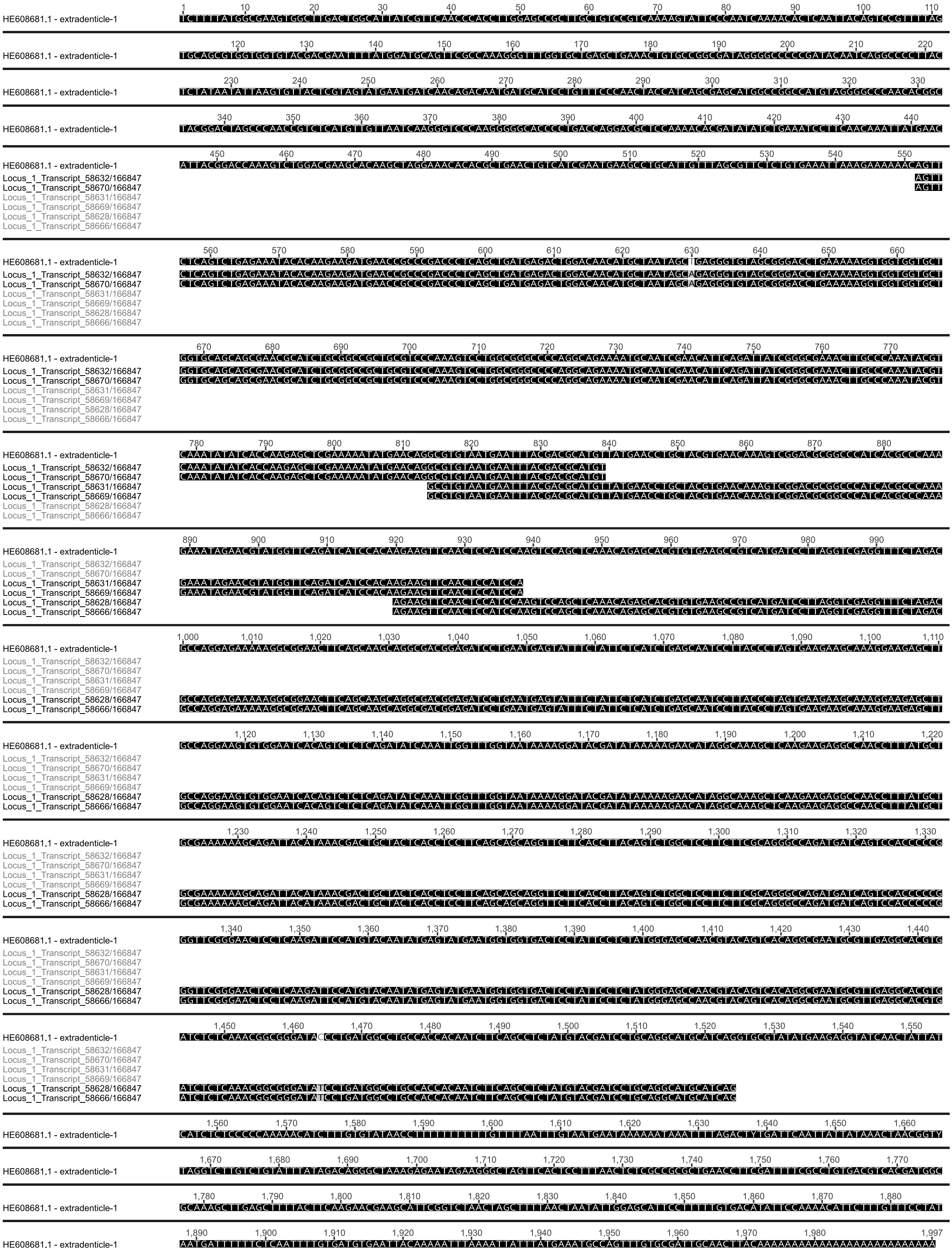

Figure S6. Alignment of short transcriptomic *exd* sequences with the predicted sequence for *exd-1*.

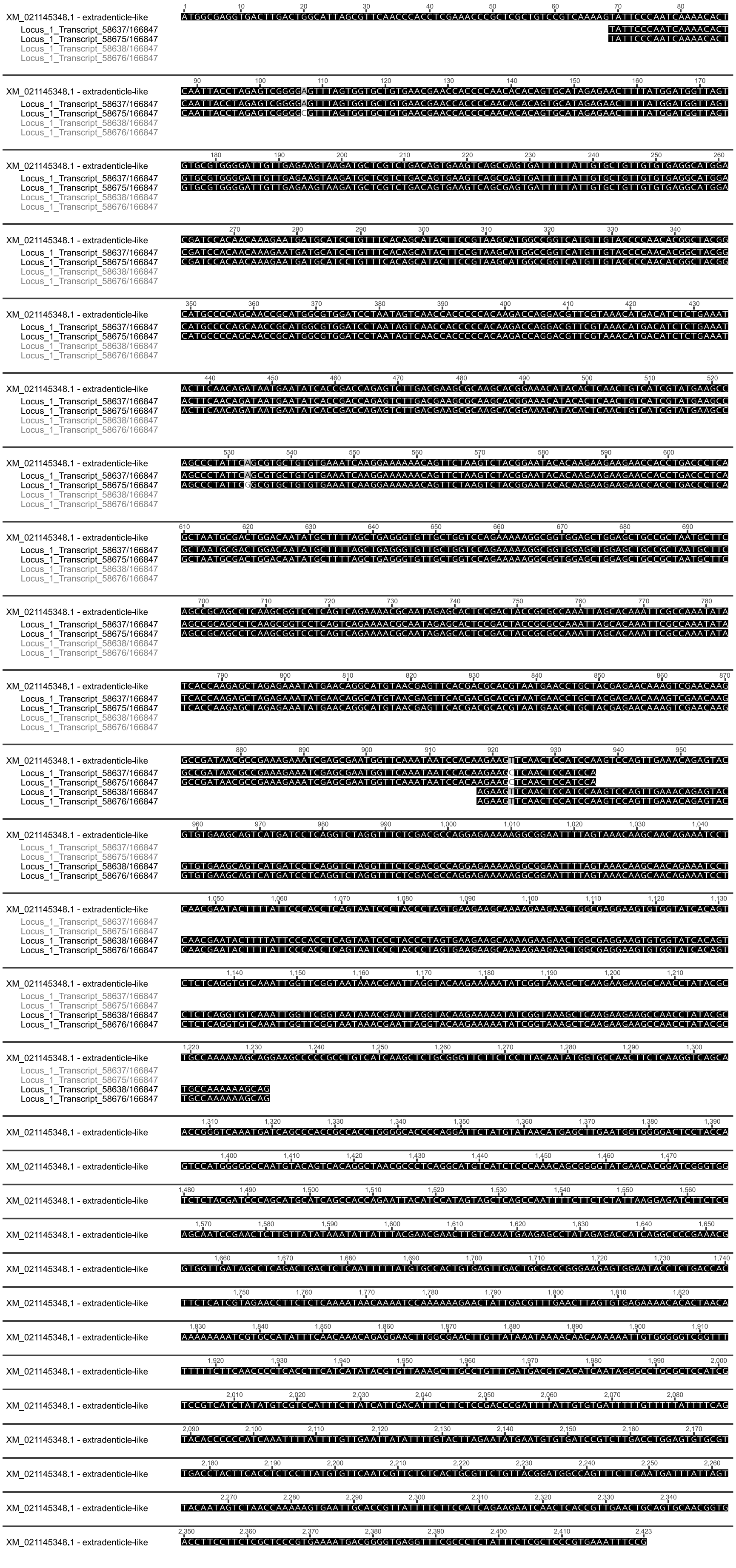

Figure S7. Alignment of short transcriptomic *exd* sequences with the predicted sequence of *exd-2*.

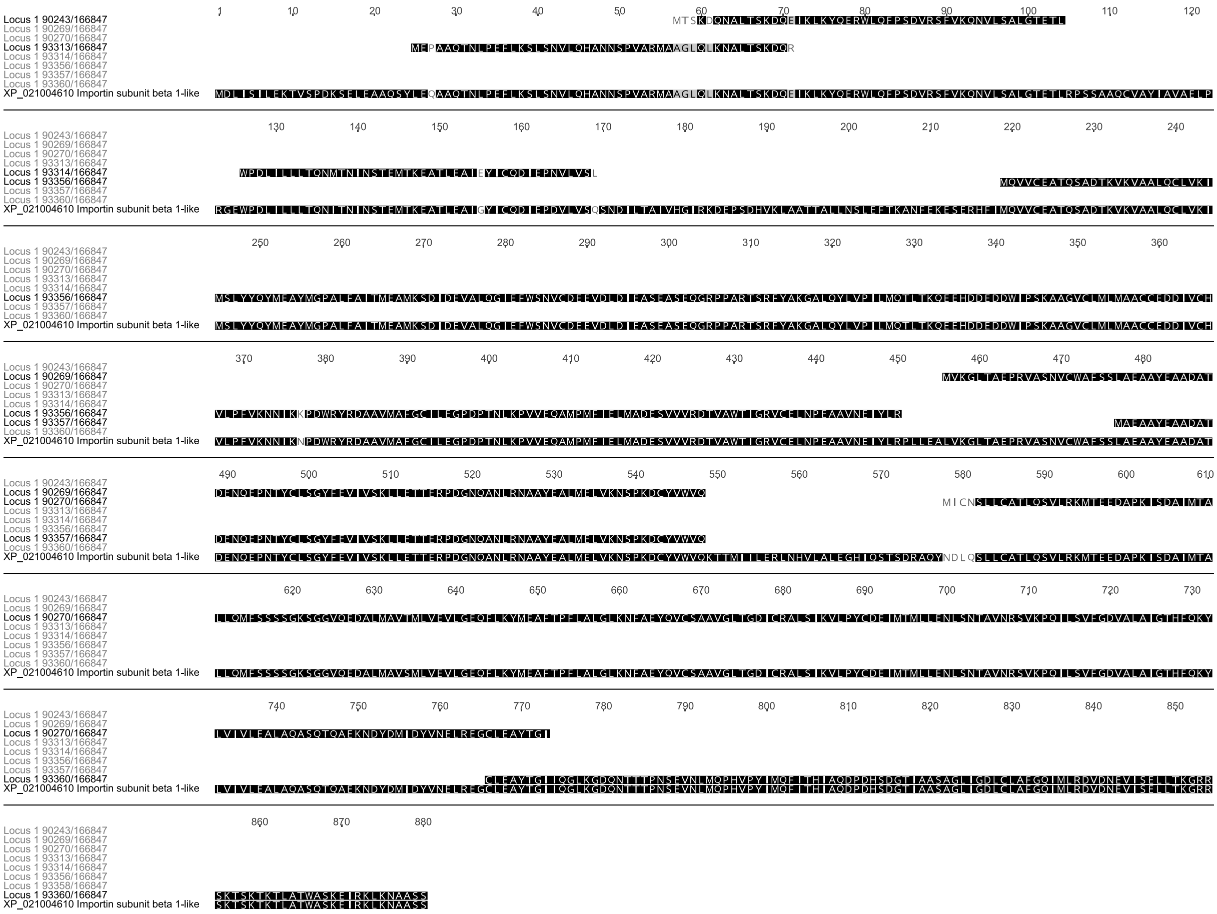

Figure S8. Alignment of short translated Fs(2)Ket sequences with the predicted Fs(2)Ket sequence.

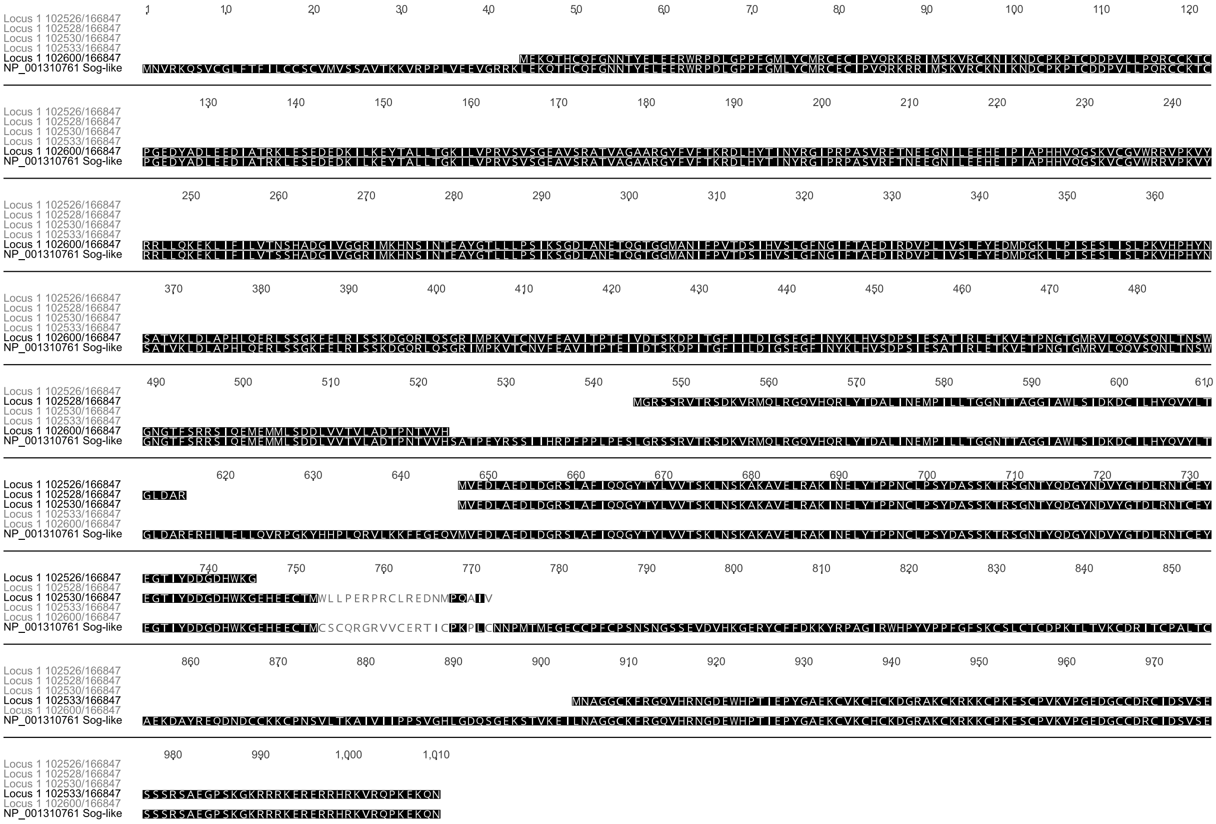

Figure S9. Alignment of short translated transcriptomic Sog sequences with the published Sog sequence.

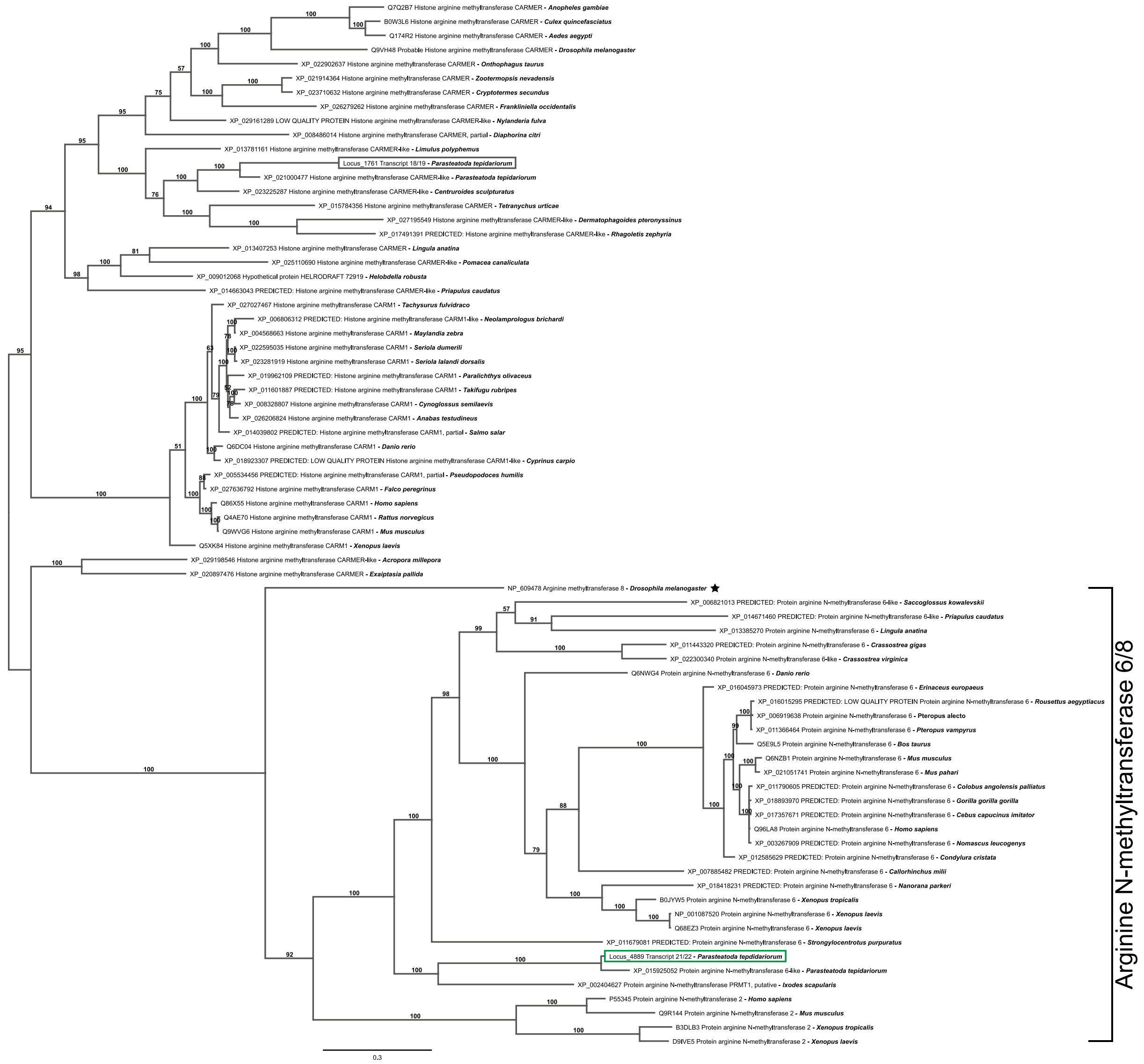

Figure S10. Bayesian phylogenetic tree of Art8. Sequence used for the initial BLAST search is marked with a star, Homologous *Parasteatoda* sequences marked with green box, other, non-homologous *Parasteatoda* sequences marked with grey box. Branch labels indicate posterior probability as determined by MrBayes.

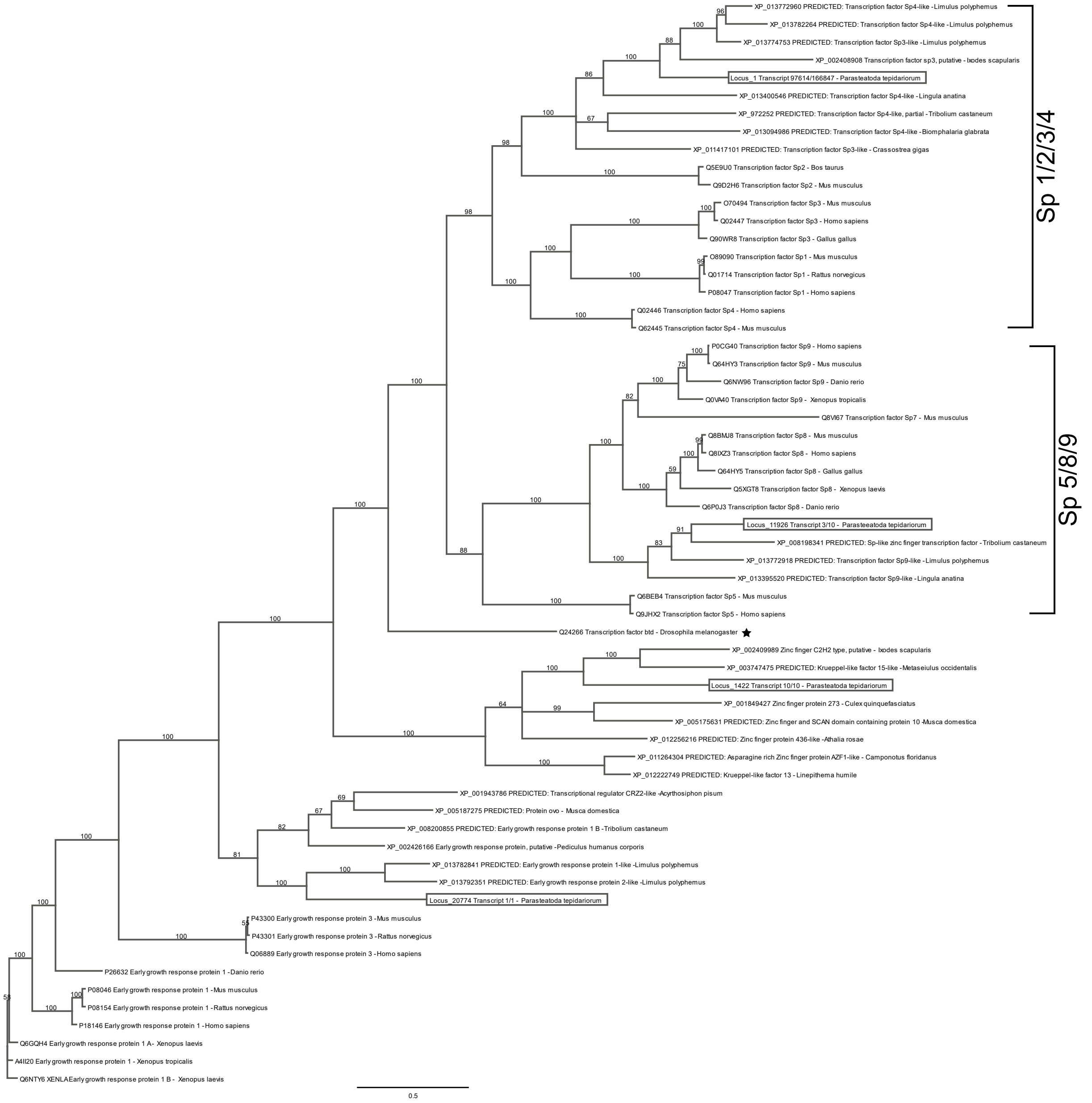

Figure S11. Bayesian phylogenetic tree of btd. Sequence used for the initial BLAST search is marked with a star, Homologous *Parasteatoda* sequences marked with green box, other, non-homologous *Parasteatoda* sequences marked with grey box. Branch labels indicate posterior probability as determined by MrBayes.

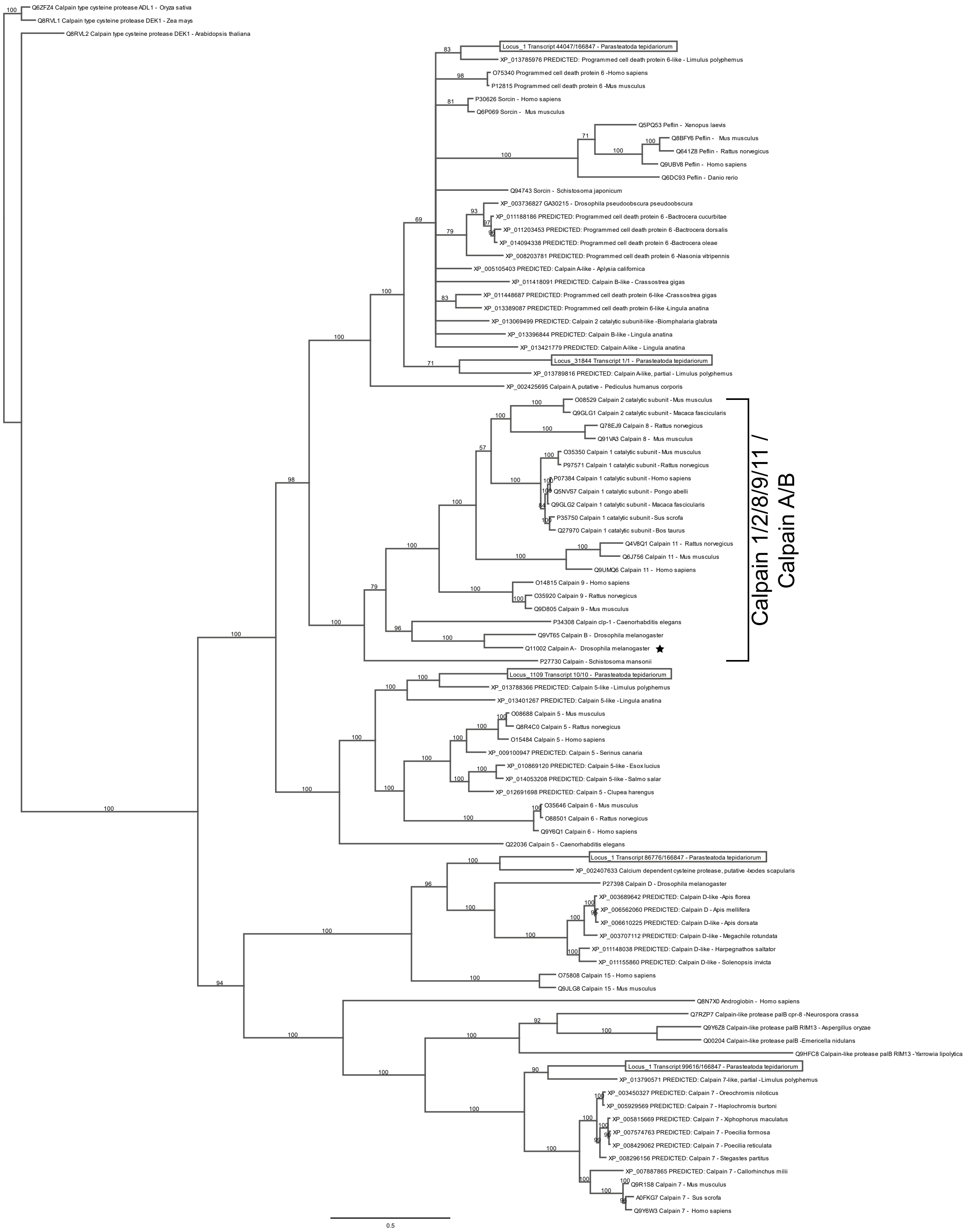

Figure S12. Bayesian phylogenetic tree of CalpA. Sequence used for the initial BLAST search is marked with a star, Homologous *Parasteatoda* sequences marked with green box, other, non-homologous *Parasteatoda* sequences marked with grey box. Branch labels indicate posterior probability as determined by MrBayes.

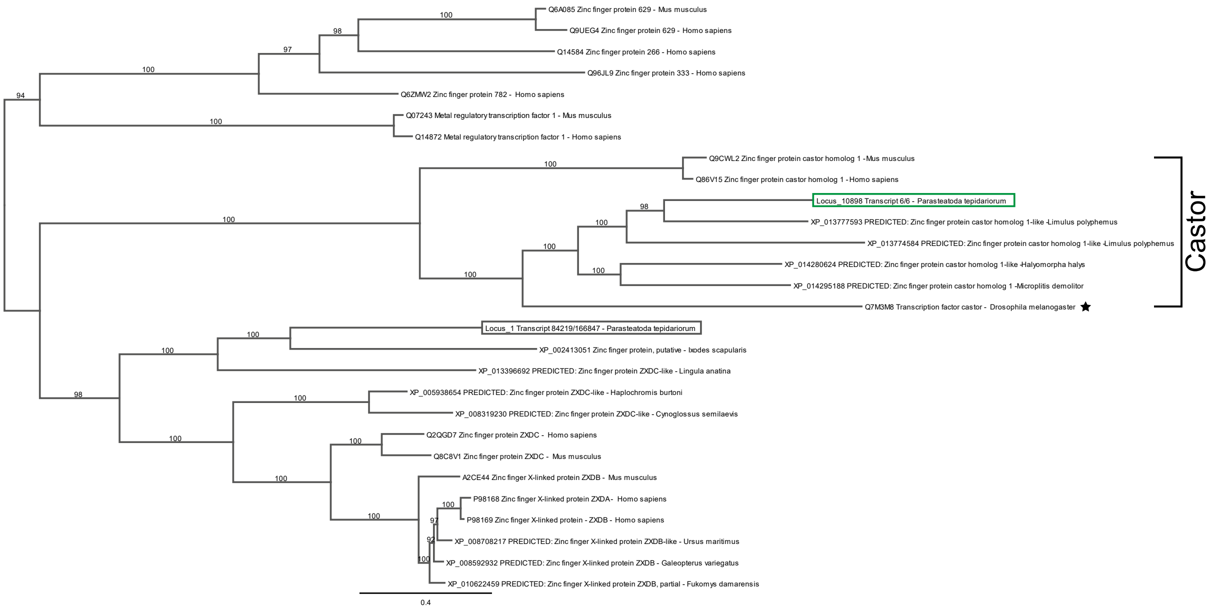

Figure S13. Bayesian phylogenetic tree of cas. Sequence used for the initial BLAST search is marked with a star, Homologous *Parasteatoda* sequences marked with green box, other, non-homologous *Parasteatoda* sequences marked with grey box. Branch labels indicate posterior probability as determined by MrBayes.

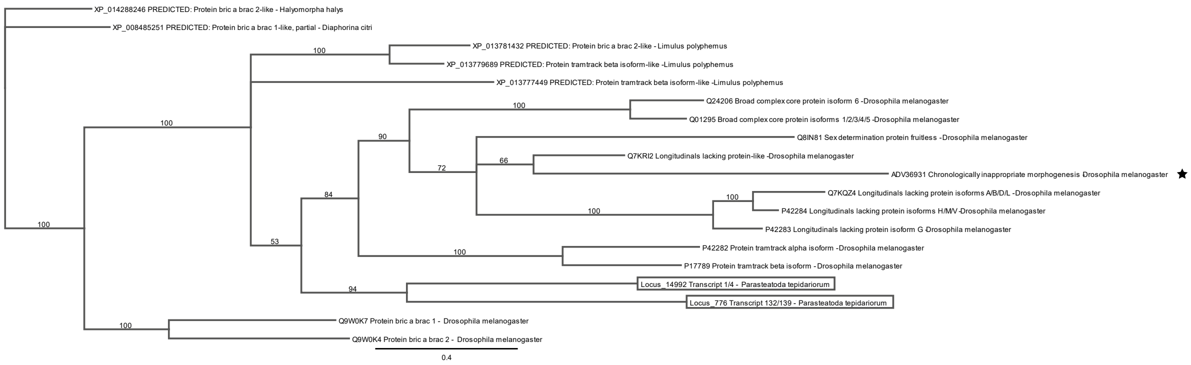

Figure S14. Bayesian phylogenetic tree of chinmo. Sequence used for the initial BLAST search is marked with a star, Homologous *Parasteatoda* sequences marked with green box, other, non-homologous *Parasteatoda* sequences marked with grey box. Branch labels indicate posterior probability as determined by MrBayes.

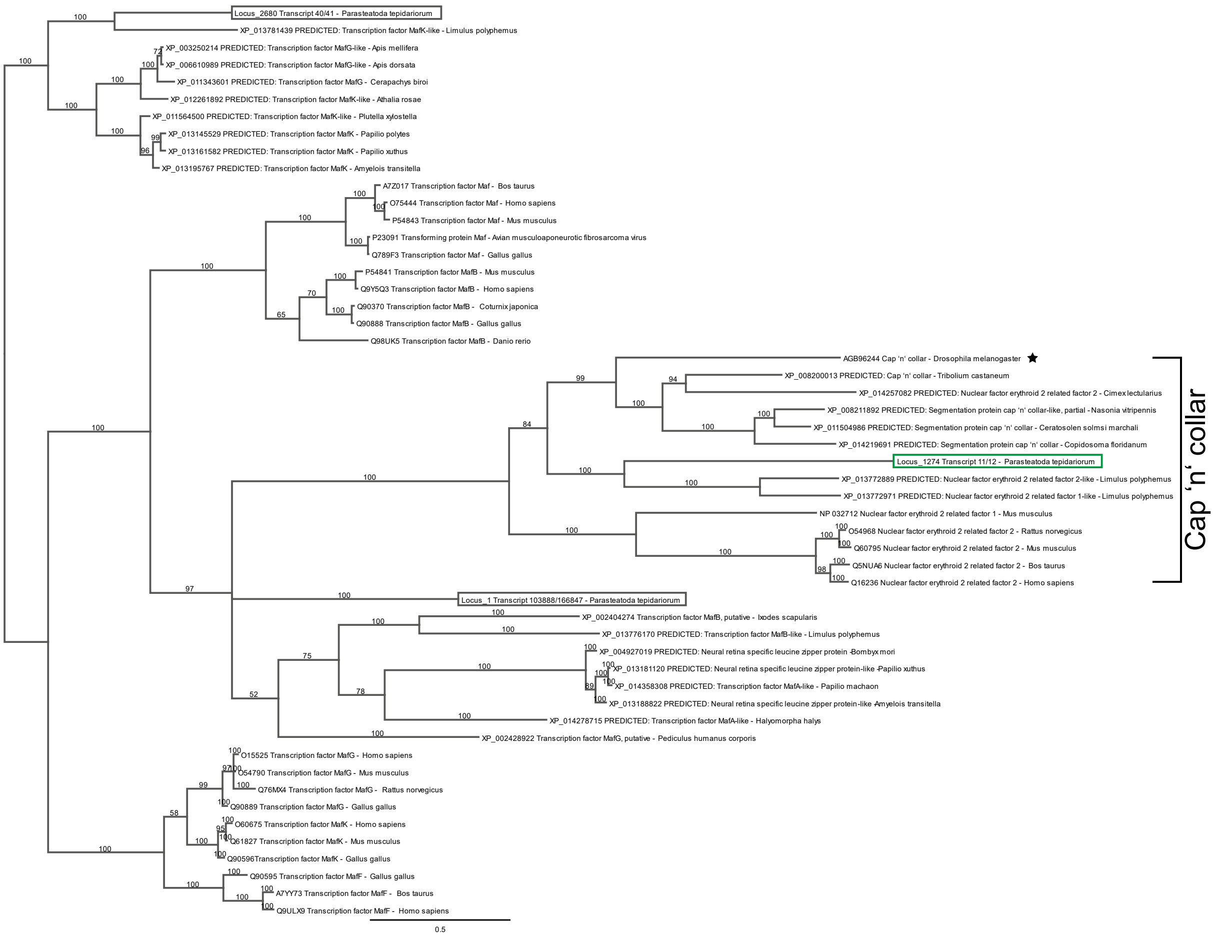

Figure S15. Bayesian phylogenetic tree of cnc. Sequence used for the initial BLAST search is marked with a star, Homologous *Parasteatoda* sequences marked with green box, other, non-homologous *Parasteatoda* sequences marked with grey box. Branch labels indicate posterior probability as determined by MrBayes.

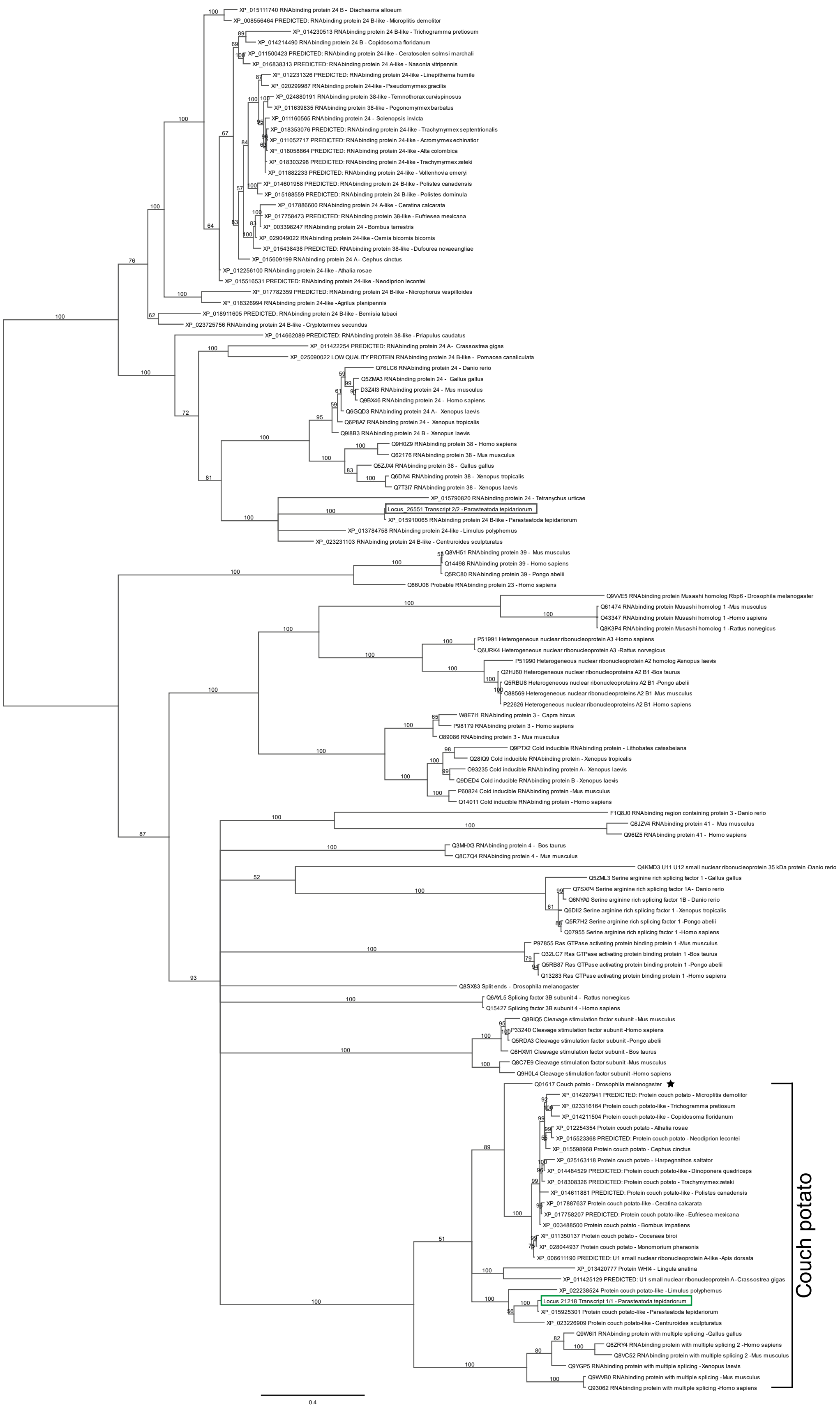

Figure S16. Bayesian phylogenetic tree of cpo. Sequence used for the initial BLAST search is marked with a star, Homologous *Parasteatoda* sequences marked with green box, other, non-homologous *Parasteatoda* sequences marked with grey box. Branch labels indicate posterior probability as determined by MrBayes.

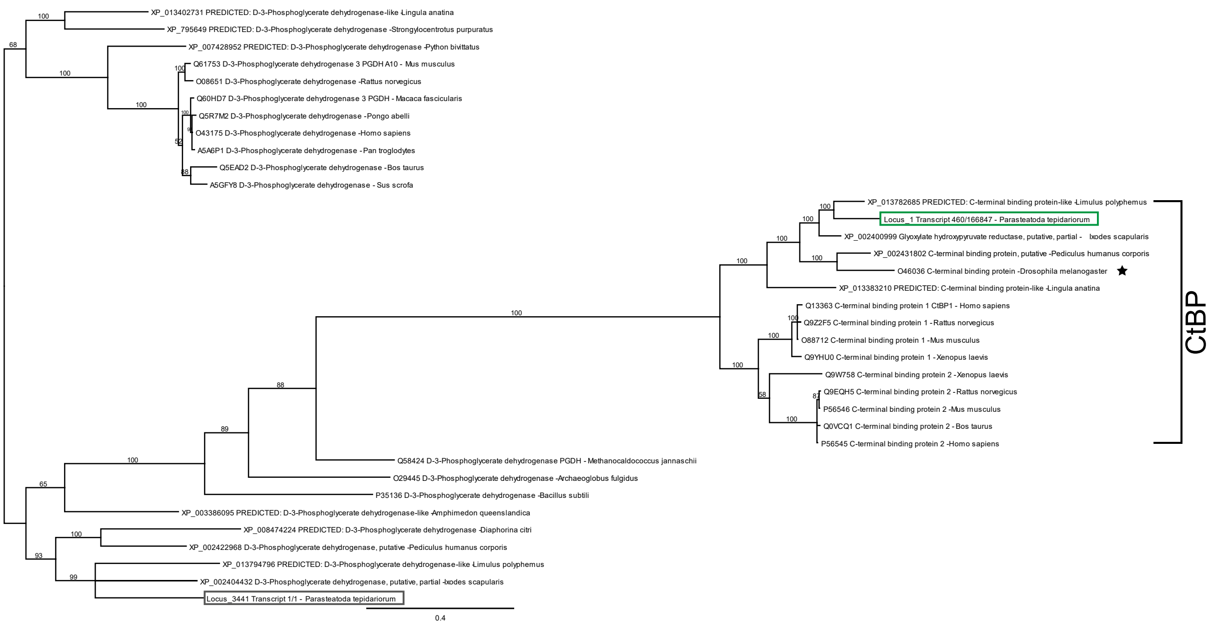

Figure S17. Bayesian phylogenetic tree of CtBP. Sequence used for the initial BLAST search is marked with a star, Homologous *Parasteatoda* sequences marked with green box, other, non-homologous *Parasteatoda* sequences marked with grey box. Branch labels indicate posterior probability as determined by MrBayes.

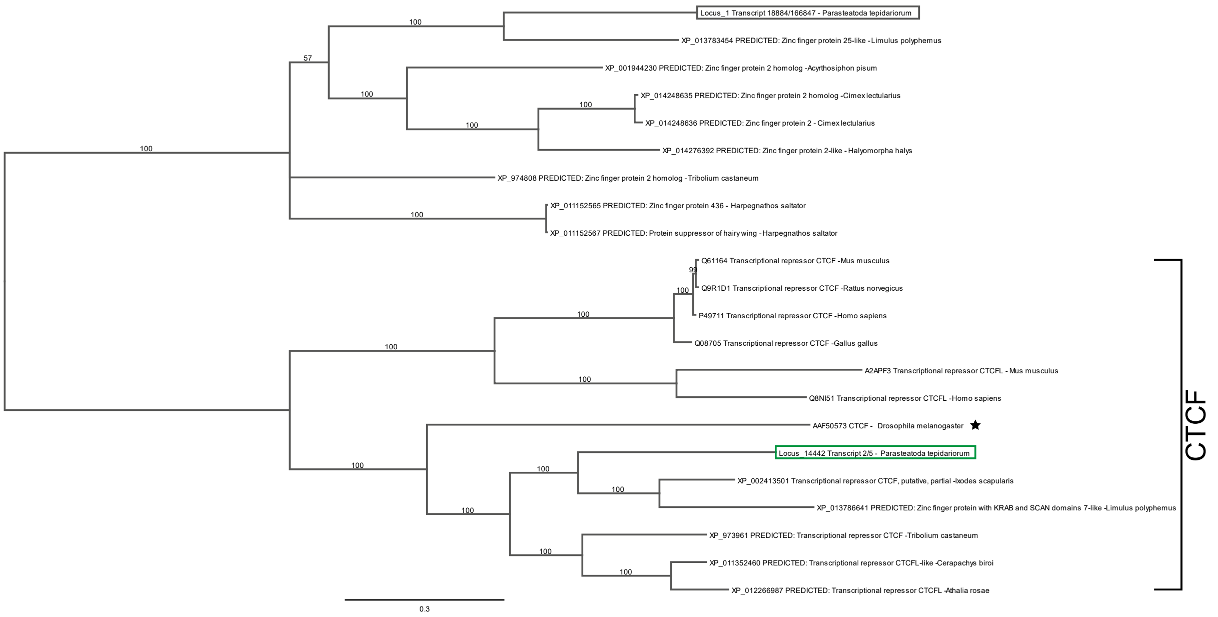

Figure S18. Bayesian phylogenetic tree of CTCF. Sequence used for the initial BLAST search is marked with a star, Homologous *Parasteatoda* sequences marked with green box, other, non-homologous *Parasteatoda* sequences marked with grey box. Branch labels indicate posterior probability as determined by MrBayes.

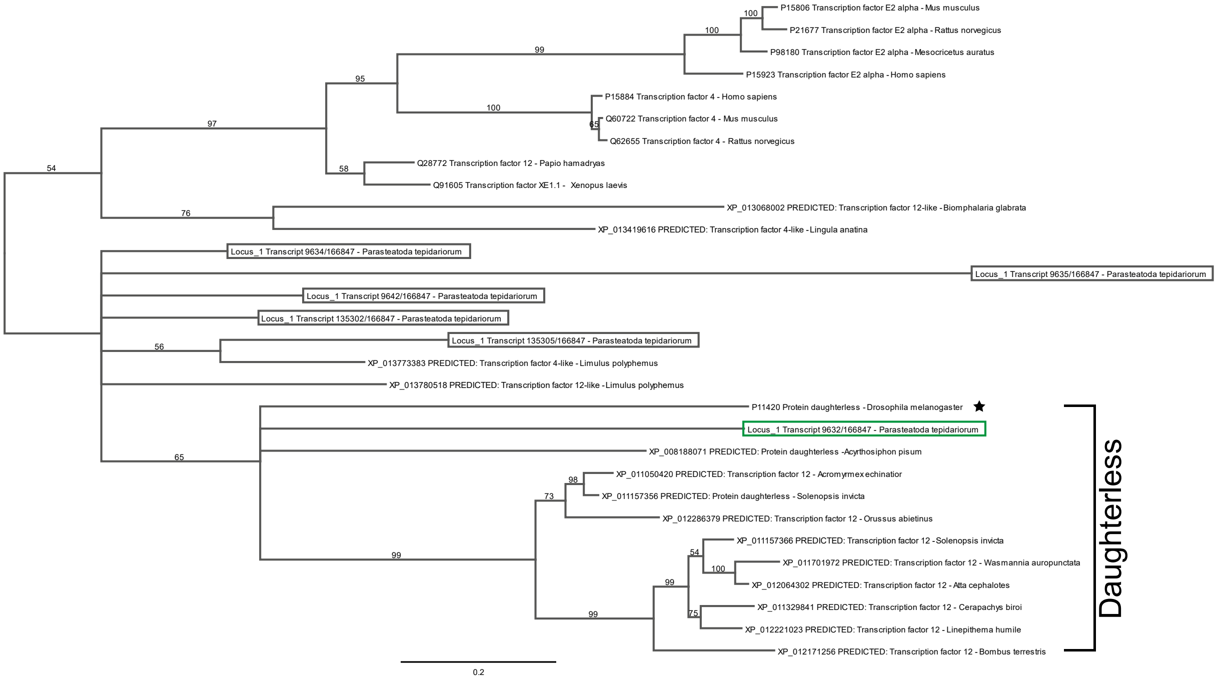

Figure S19. Bayesian phylogenetic tree of da. Sequence used for the initial BLAST search is marked with a star, Homologous *Parasteatoda* sequences marked with green box, other, non-homologous *Parasteatoda* sequences marked with grey box. Branch labels indicate posterior probability as determined by MrBayes.

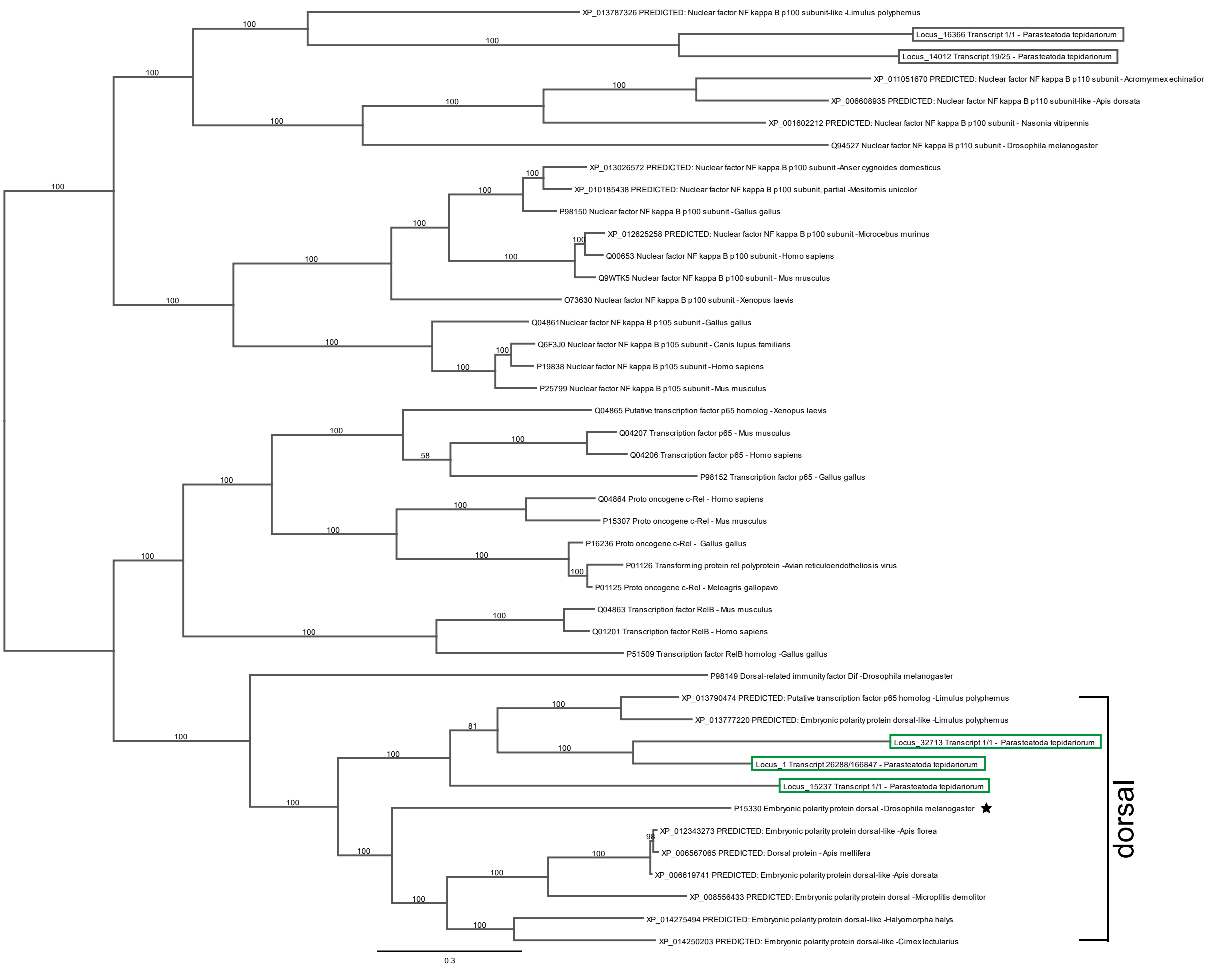

Figure S20. Bayesian phylogenetic tree of dl. Sequence used for the initial BLAST search is marked with a star, Homologous *Parasteatoda* sequences marked with green box, other, non-homologous *Parasteatoda* sequences marked with grey box. Branch labels indicate posterior probability as determined by MrBayes.

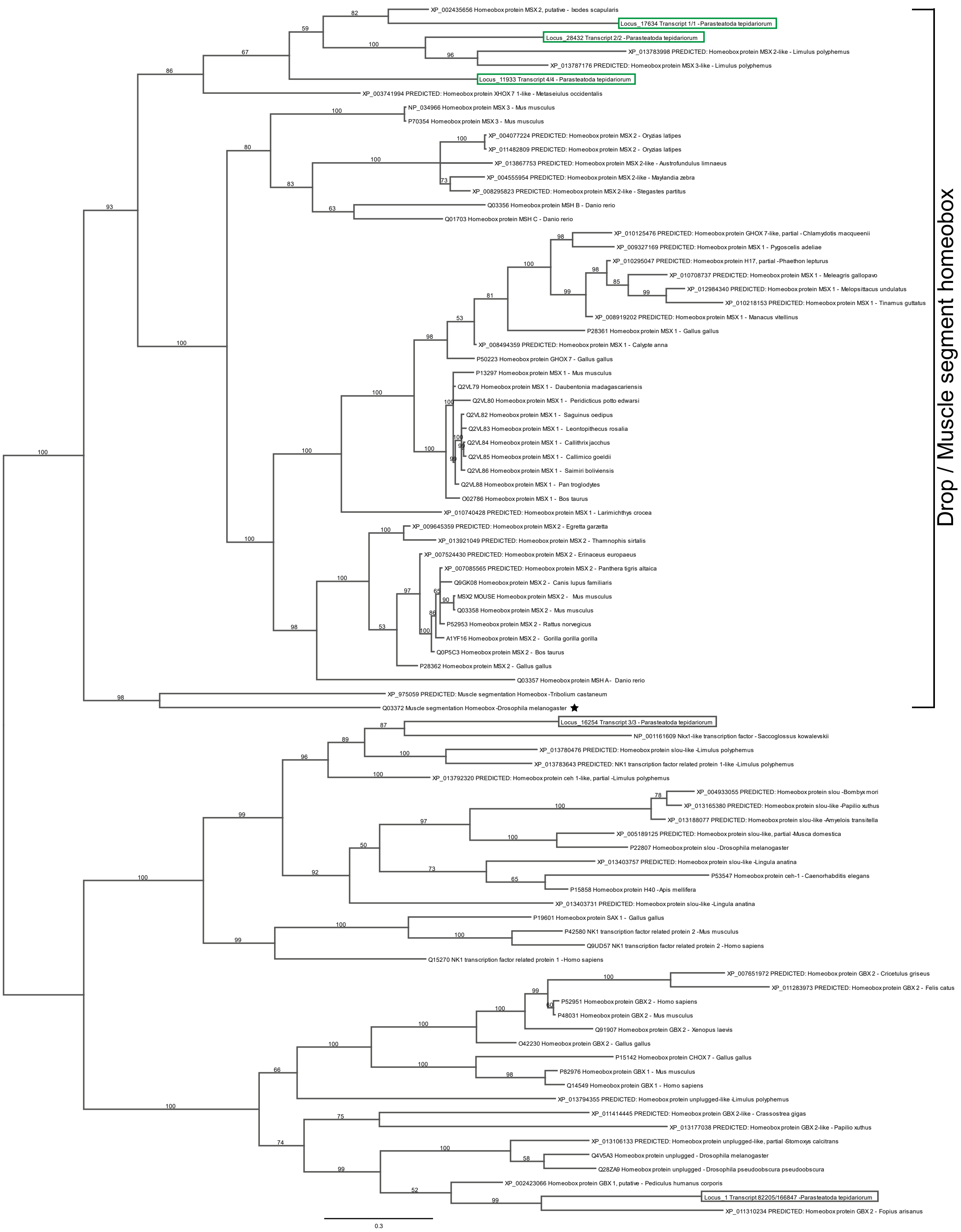

Figure S21. Bayesian phylogenetic tree of Dr / Msh. Sequence used for the initial BLAST search is marked with a star, Homologous *Parasteatoda* sequences marked with green box, other, non-homologous *Parasteatoda* sequences marked with grey box. Branch labels indicate posterior probability as determined by MrBayes.

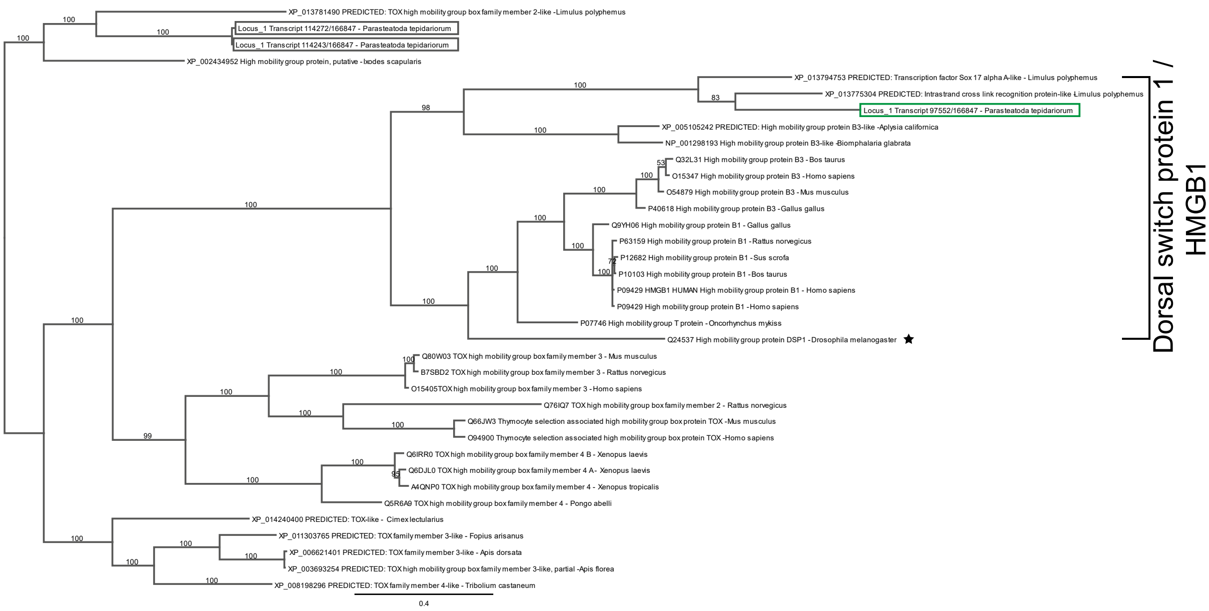

Figure S22. Bayesian phylogenetic tree of Dsp1. Sequence used for the initial BLAST search is marked with a star, Homologous *Parasteatoda* sequences marked with green box, other, non-homologous *Parasteatoda* sequences marked with grey box. Branch labels indicate posterior probability as determined by MrBayes.

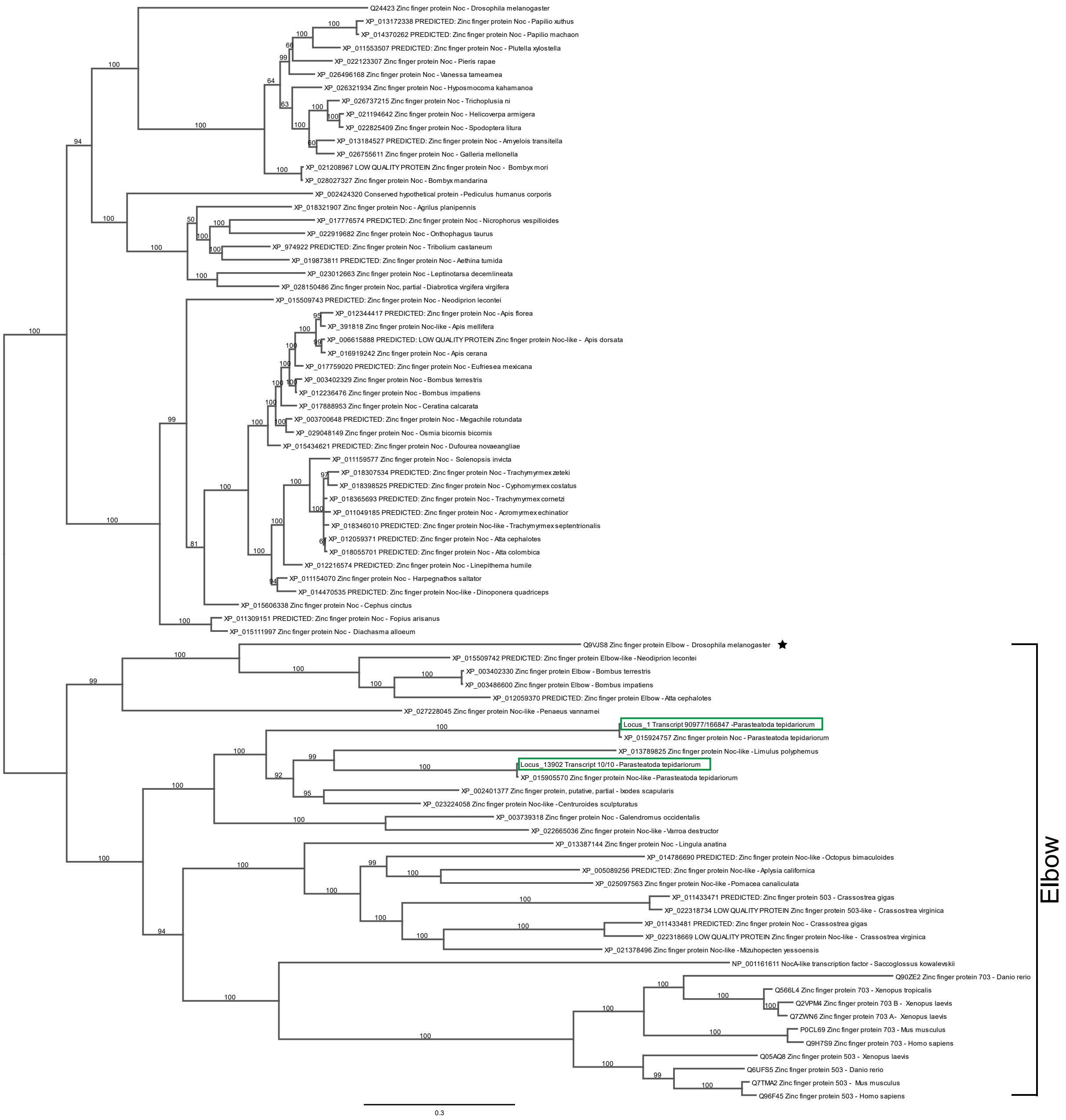

Figure S23. Bayesian phylogenetic tree of elB. Sequence used for the initial BLAST search is marked with a star, Homologous *Parasteatoda* sequences marked with green box, other, non-homologous *Parasteatoda* sequences marked with grey box. Branch labels indicate posterior probability as determined by MrBayes.

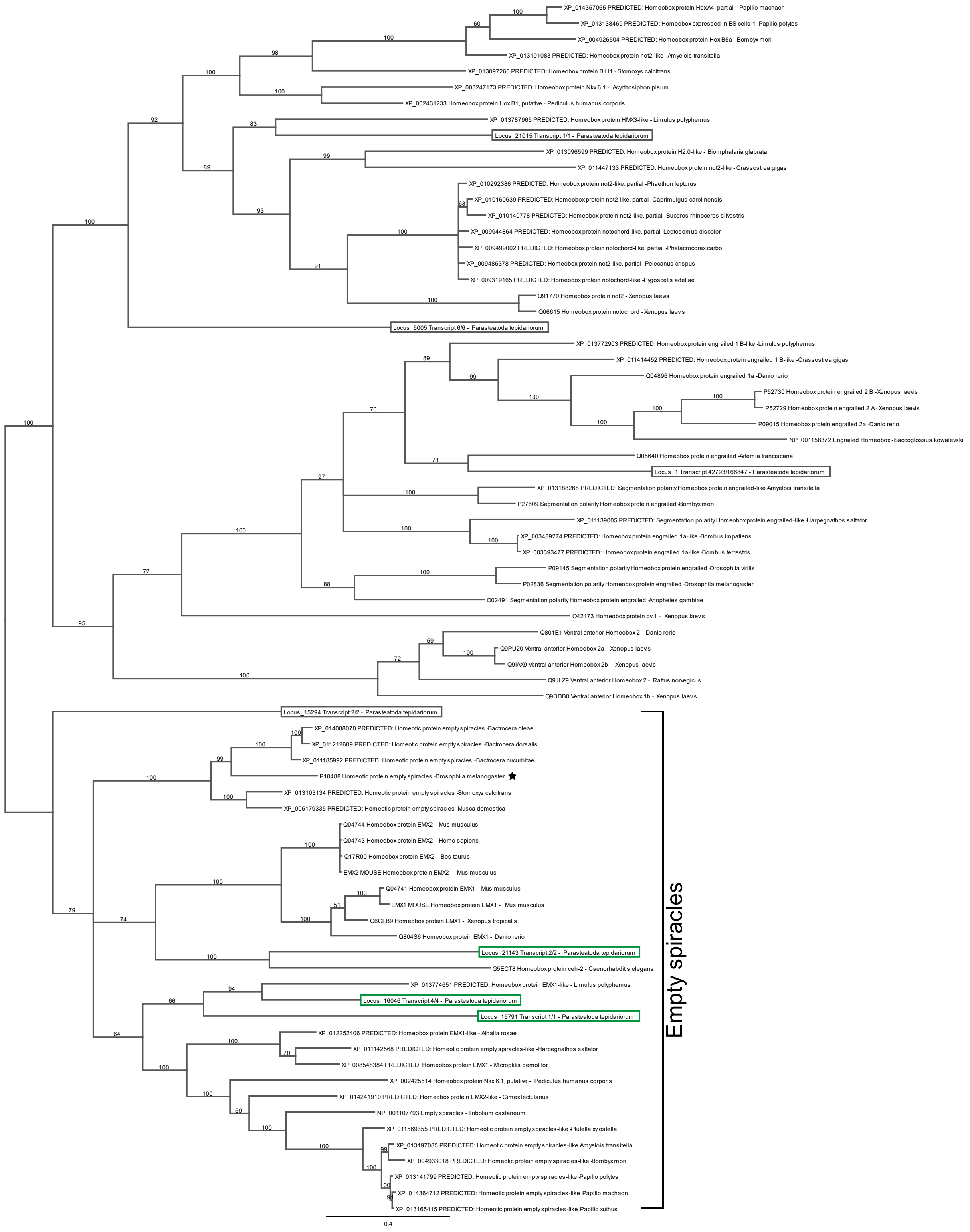

Figure S24. Bayesian phylogenetic tree of ems. Sequence used for the initial BLAST search is marked with a star, Homologous *Parasteatoda* sequences marked with green box, other, non-homologous *Parasteatoda* sequences marked with grey box. Branch labels indicate posterior probability as determined by MrBayes.

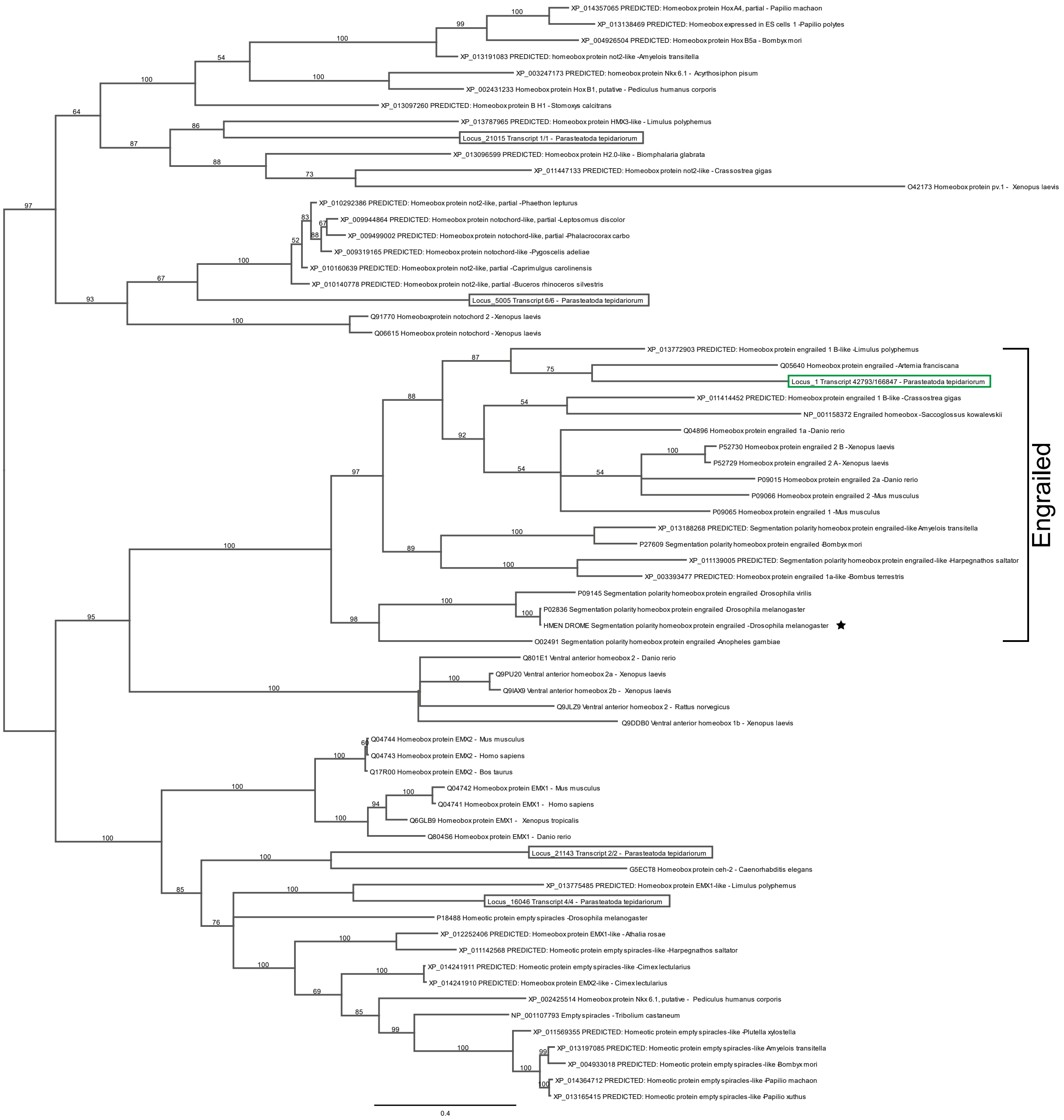

Figure S25. Bayesian phylogenetic tree of en. Sequence used for the initial BLAST search is marked with a star, Homologous *Parasteatoda* sequences marked with green box, other, non-homologous *Parasteatoda* sequences marked with grey box. Branch labels indicate posterior probability as determined by MrBayes.

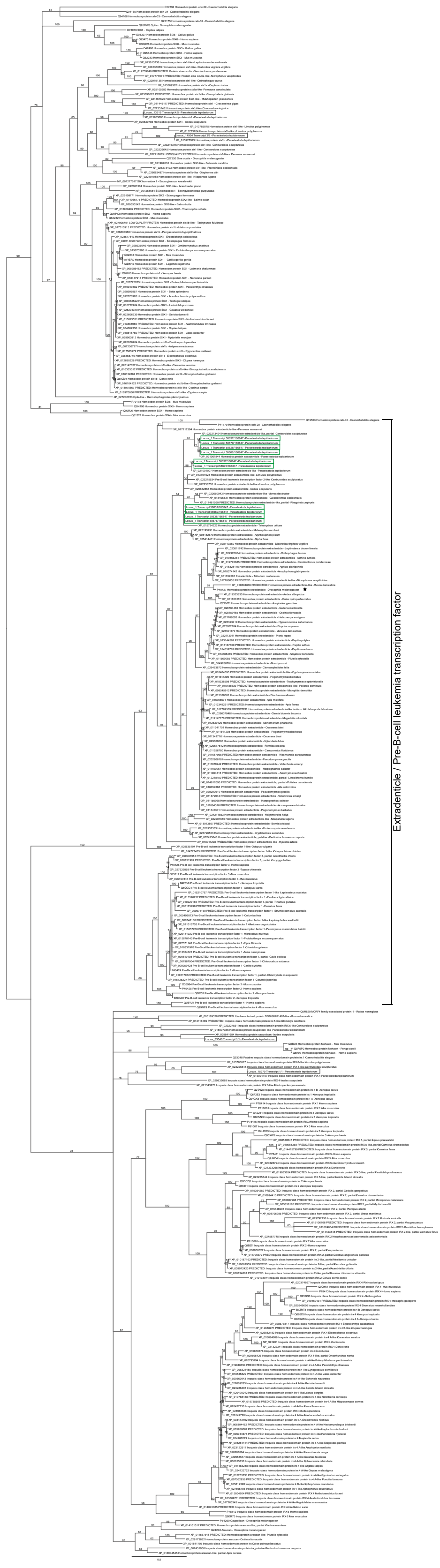

Figure S26. Bayesian phylogenetic tree of exd. Sequence used for the initial BLAST search is marked with a star, Homologous *Parasteatoda* sequences marked with green box, other, non-homologous *Parasteatoda* sequences marked with grey box. Branch labels indicate posterior probability as determined by MrBayes.

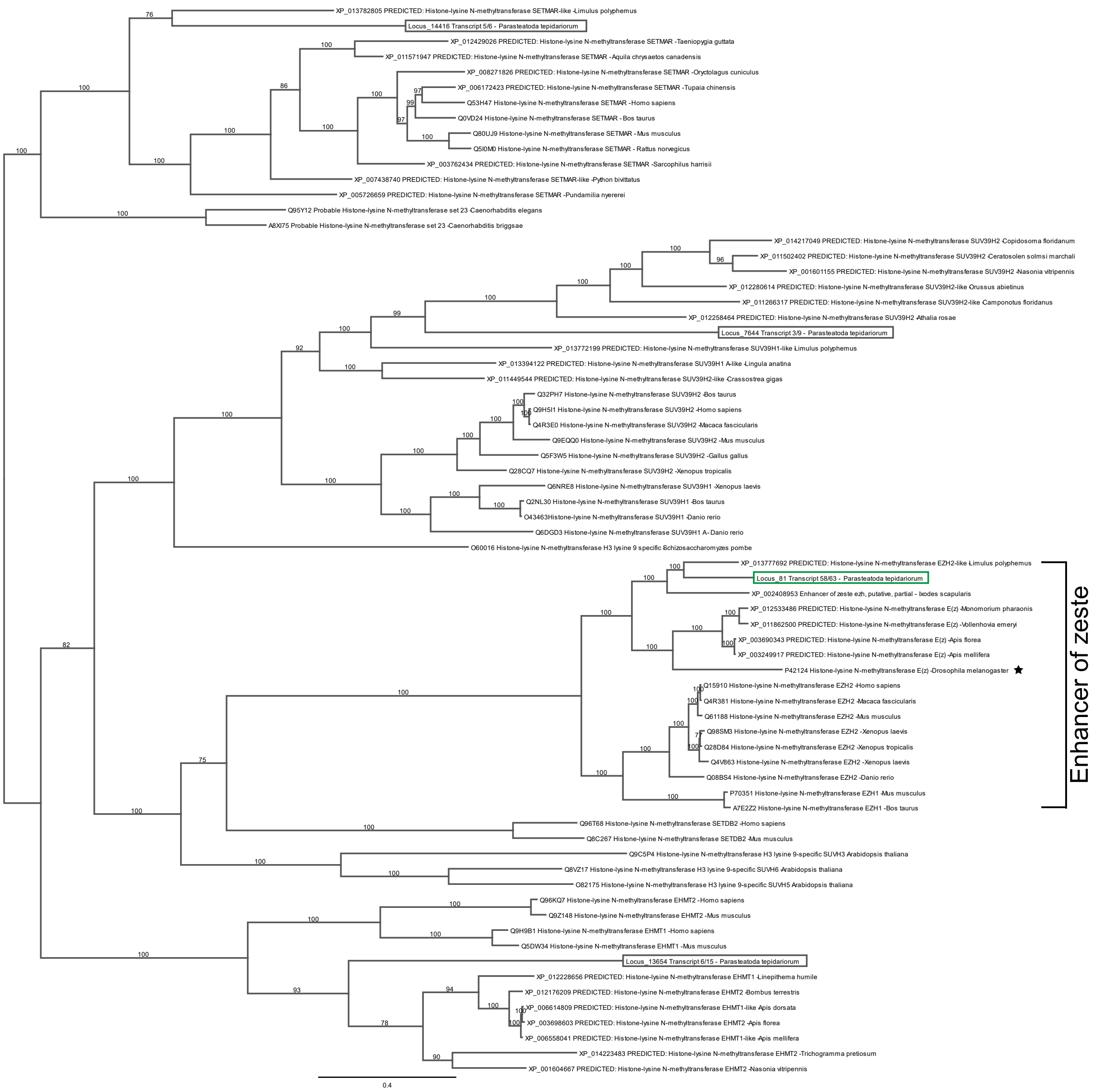

Figure S27. Bayesian phylogenetic tree of E(z). Sequence used for the initial BLAST search is marked with a star, Homologous *Parasteatoda* sequences marked with green box, other, non-homologous *Parasteatoda* sequences marked with grey box. Branch labels indicate posterior probability as determined by MrBayes.

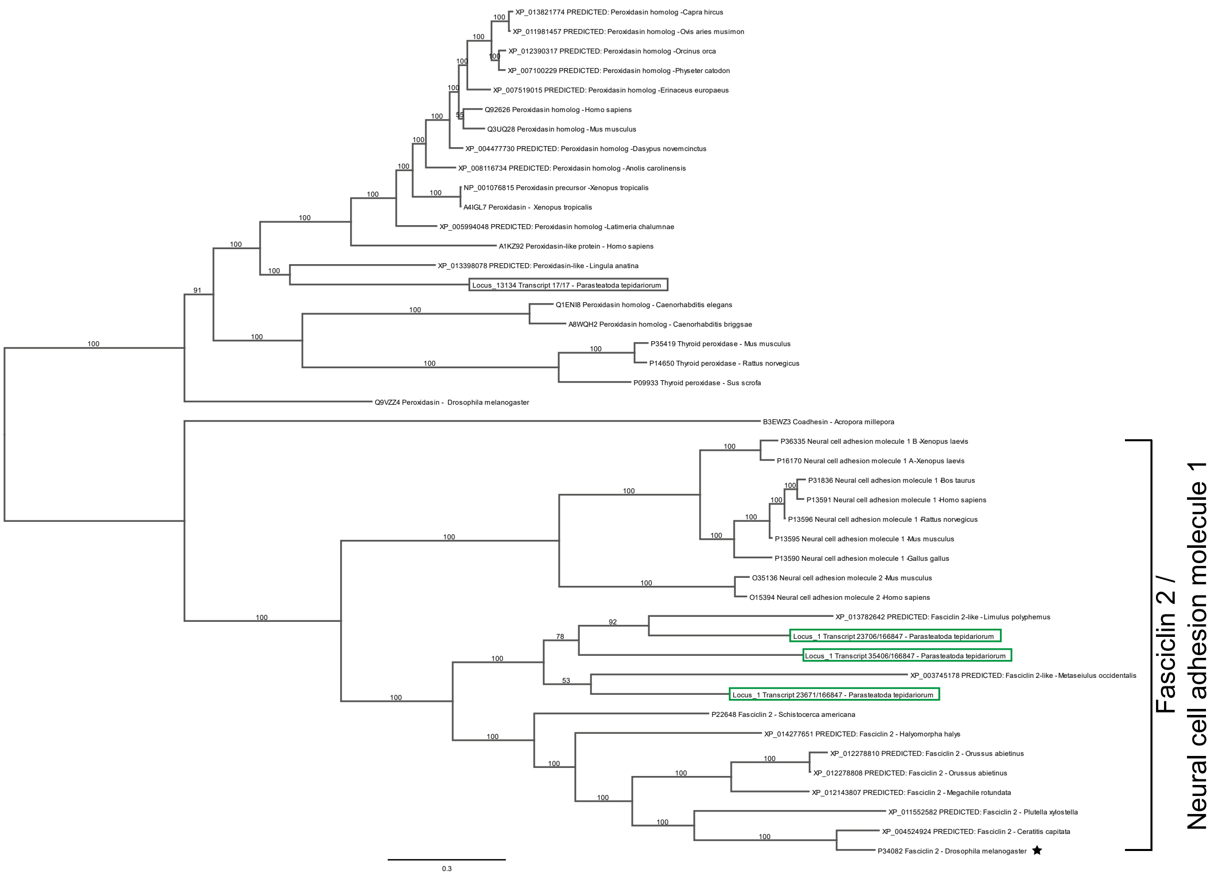

Figure S28. Bayesian phylogenetic tree of Fas2. Sequence used for the initial BLAST search is marked with a star, Homologous *Parasteatoda* sequences marked with green box, other, non-homologous *Parasteatoda* sequences marked with grey box. Branch labels indicate posterior probability as determined by MrBayes.

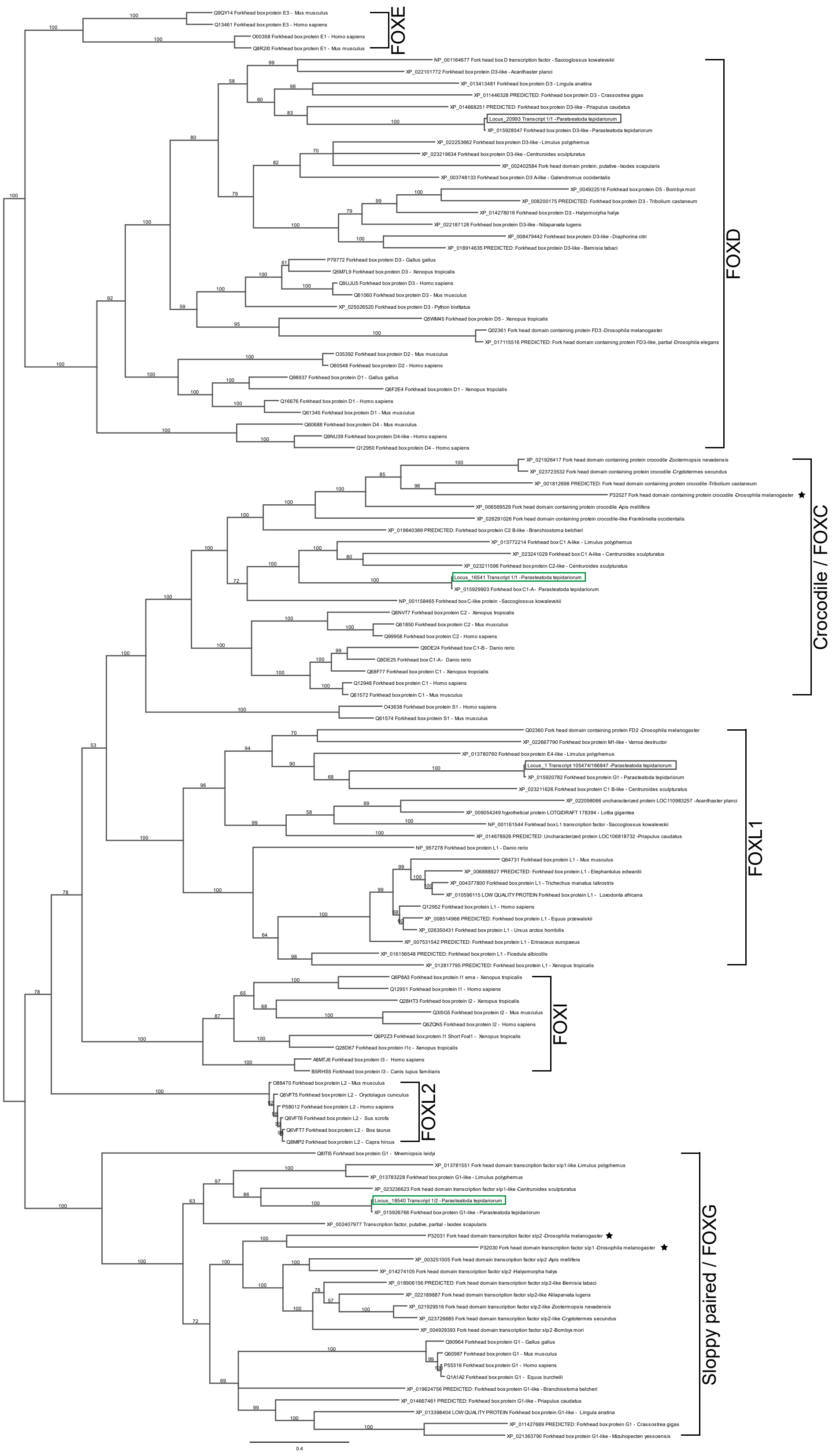

Figure S29. Bayesian phylogenetic tree of FOX genes. Sequence used for the initial BLAST search is marked with a star, Homologous *Parasteatoda* sequences marked with green box, other, non-homologous *Parasteatoda* sequences marked with grey box. Branch labels indicate posterior probability as determined by MrBayes.

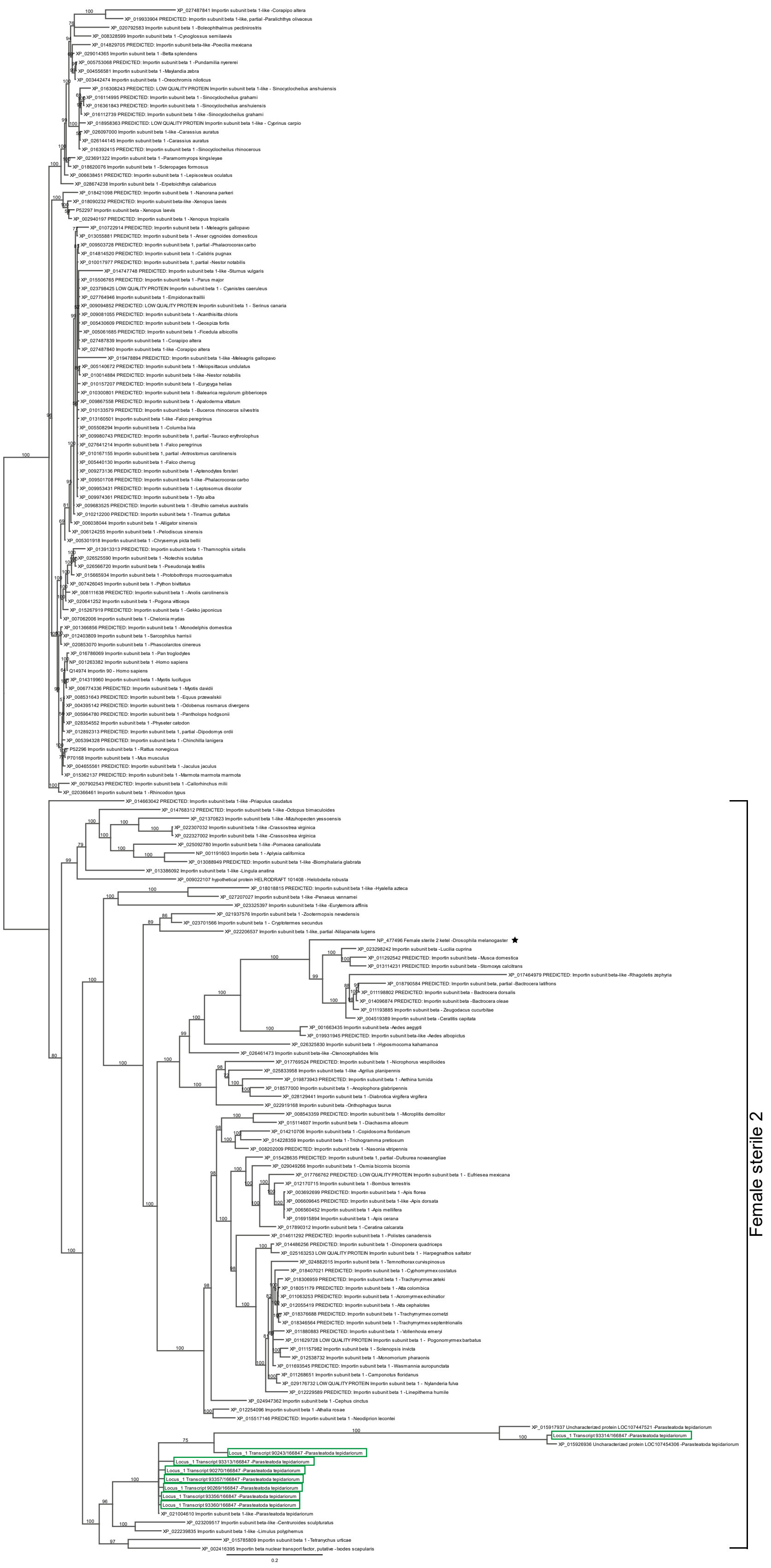

Figure S30. Bayesian phylogenetic tree of Fs(2)Ket. Sequence used for the initial BLAST search is marked with a star, Homologous *Parasteatoda* sequences marked with green box, other, non-homologous *Parasteatoda* sequences marked with grey box. Branch labels indicate posterior probability as determined by MrBayes.

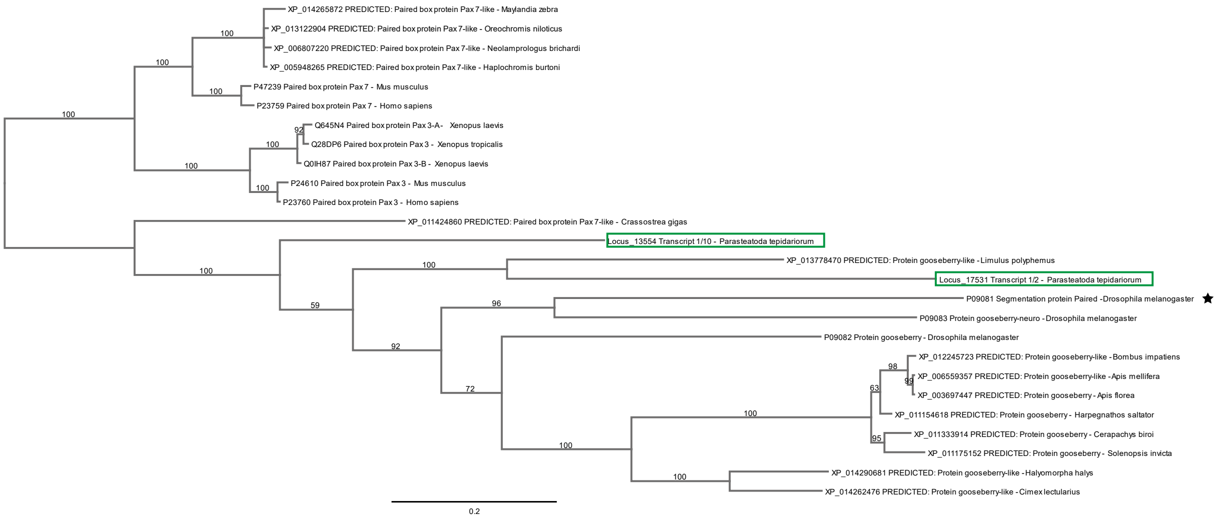

Figure S31. Bayesian phylogenetic tree of gsb / prd. Sequence used for the initial BLAST search is marked with a star, Homologous *Parasteatoda* sequences marked with green box, other, non-homologous *Parasteatoda* sequences marked with grey box. Branch labels indicate posterior probability as determined by MrBayes.

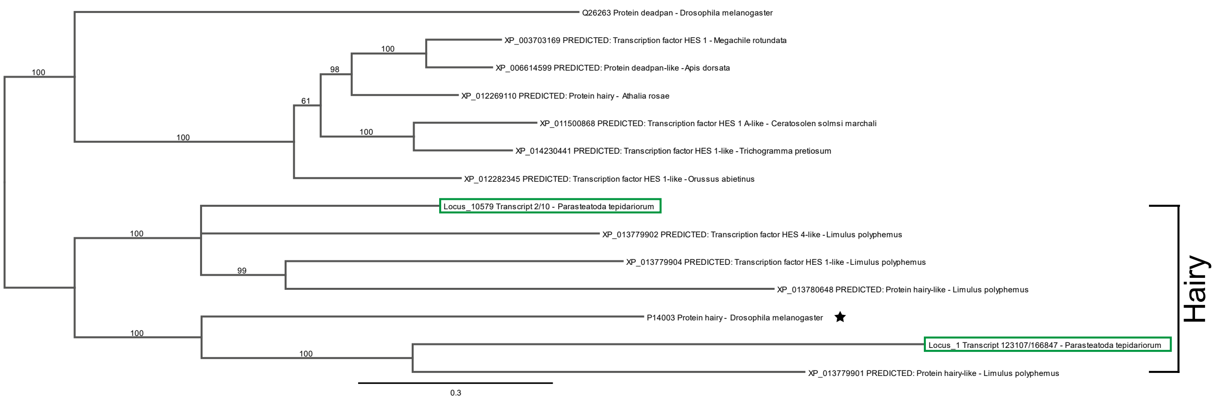

Figure S32. Bayesian phylogenetic tree of h. Sequence used for the initial BLAST search is marked with a star, Homologous *Parasteatoda* sequences marked with green box, other, non-homologous *Parasteatoda* sequences marked with grey box. Branch labels indicate posterior probability as determined by MrBayes.

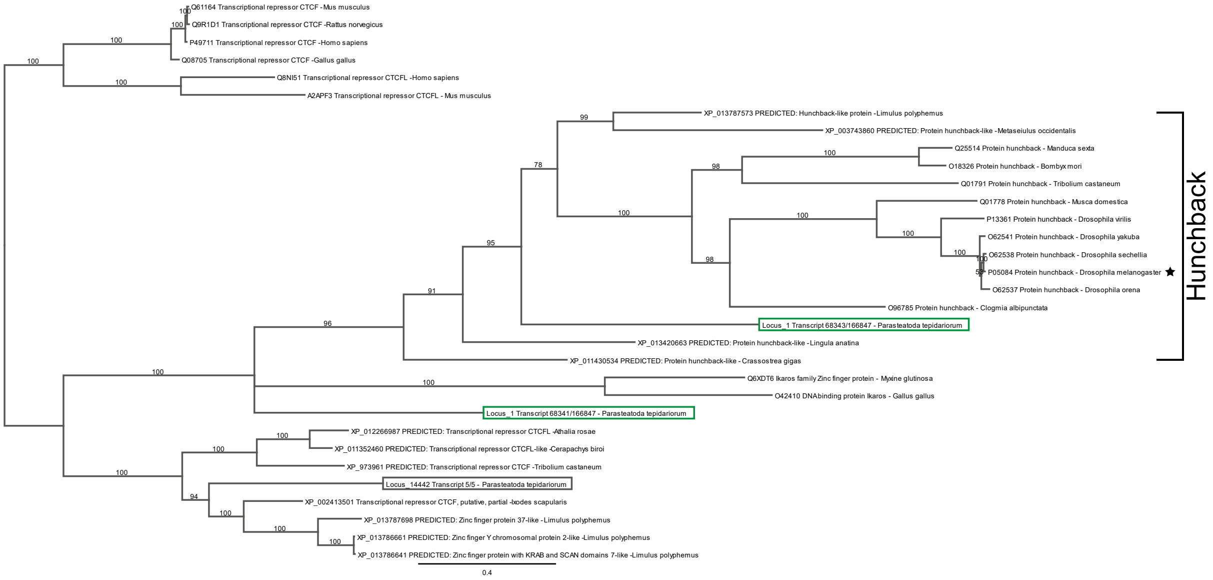

Figure S33. Bayesian phylogenetic tree of hb. Sequence used for the initial BLAST search is marked with a star, Homologous *Parasteatoda* sequences marked with green box, other, non-homologous *Parasteatoda* sequences marked with grey box. Branch labels indicate posterior probability as determined by MrBayes.

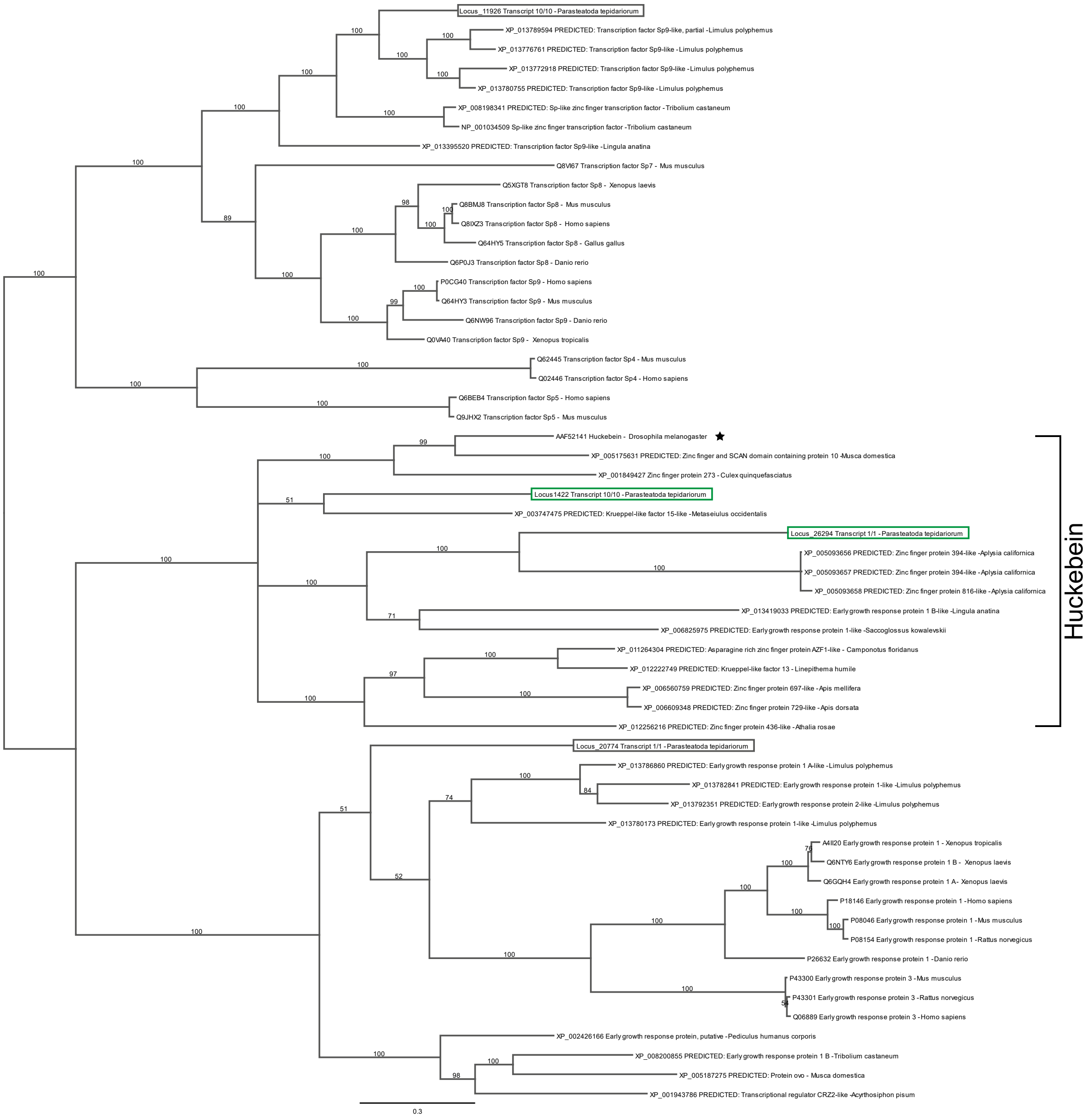

Figure S34. Bayesian phylogenetic tree of hkb. Sequence used for the initial BLAST search is marked with a star, Homologous *Parasteatoda* sequences marked with green box, other, non-homologous *Parasteatoda* sequences marked with grey box. Branch labels indicate posterior probability as determined by MrBayes.

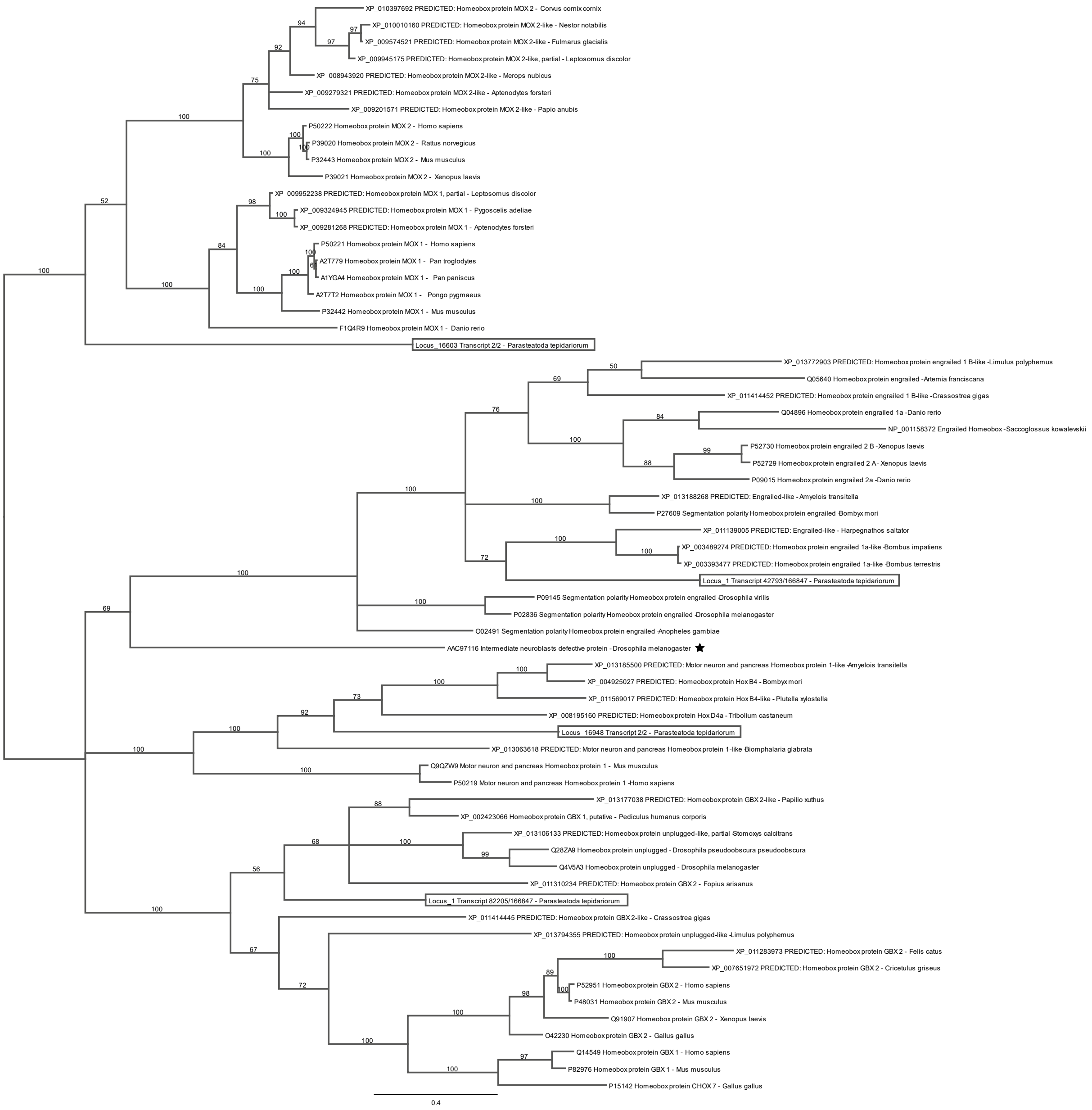

Figure S35. Bayesian phylogenetic tree of ind. Sequence used for the initial BLAST search is marked with a star, Homologous *Parasteatoda* sequences marked with green box, other, non-homologous *Parasteatoda* sequences marked with grey box. Branch labels indicate posterior probability as determined by MrBayes.

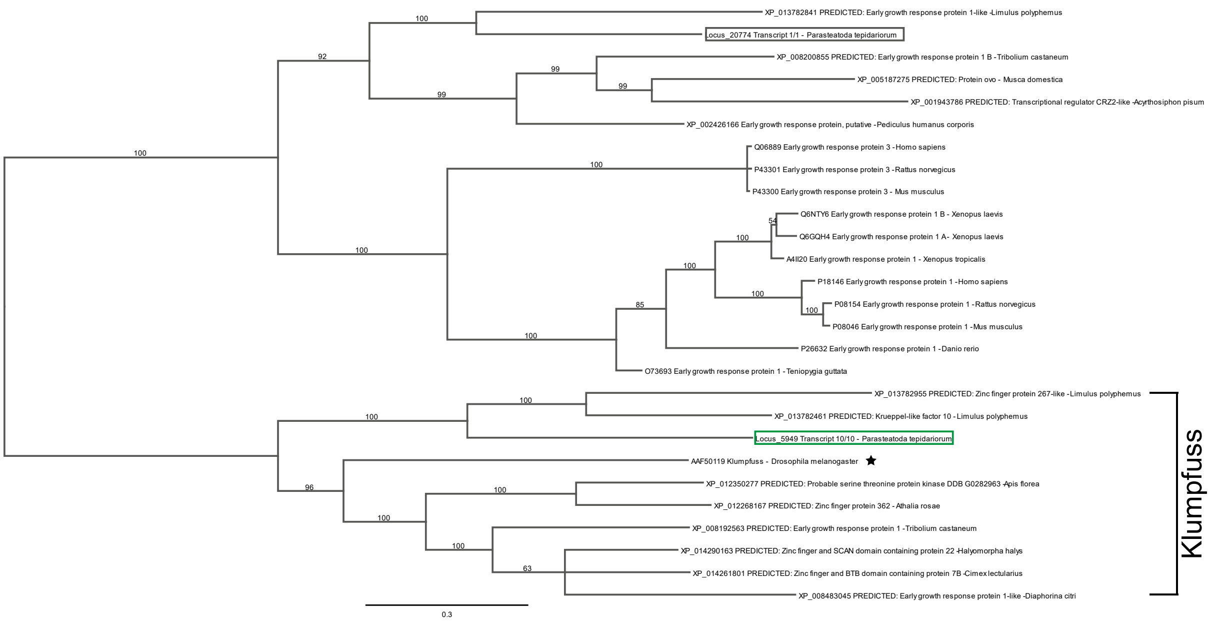

Figure S36. Bayesian phylogenetic tree of klu. Sequence used for the initial BLAST search is marked with a star, Homologous *Parasteatoda* sequences marked with green box, other, non-homologous *Parasteatoda* sequences marked with grey box. Branch labels indicate posterior probability as determined by MrBayes.

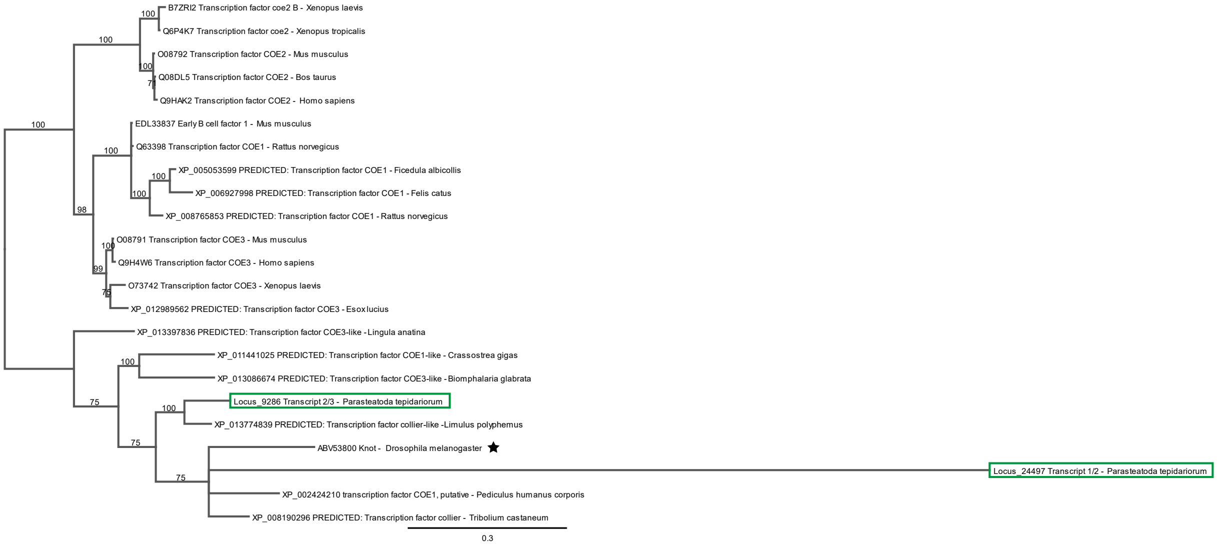

Figure S37. Bayesian phylogenetic tree of kn / col. Sequence used for the initial BLAST search is marked with a star, Homologous *Parasteatoda* sequences marked with green box, other, non-homologous *Parasteatoda* sequences marked with grey box. Branch labels indicate posterior probability as determined by MrBayes.

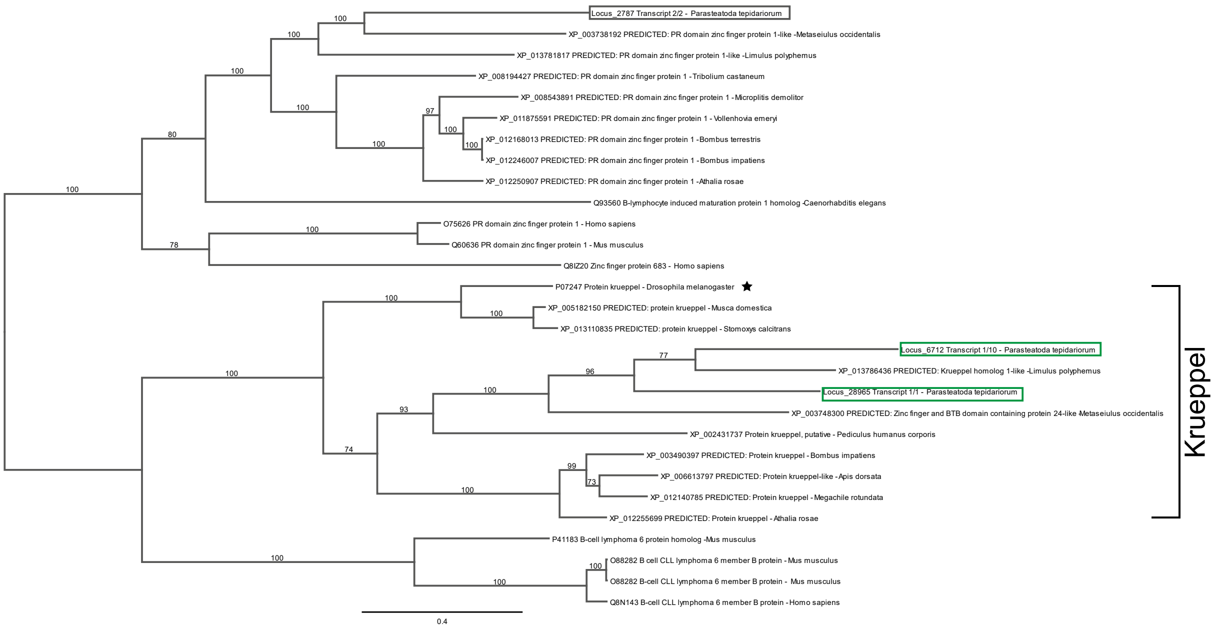

Figure S38. Bayesian phylogenetic tree of Kr. Sequence used for the initial BLAST search is marked with a star, Homologous *Parasteatoda* sequences marked with green box, other, non-homologous *Parasteatoda* sequences marked with grey box. Branch labels indicate posterior probability as determined by MrBayes.

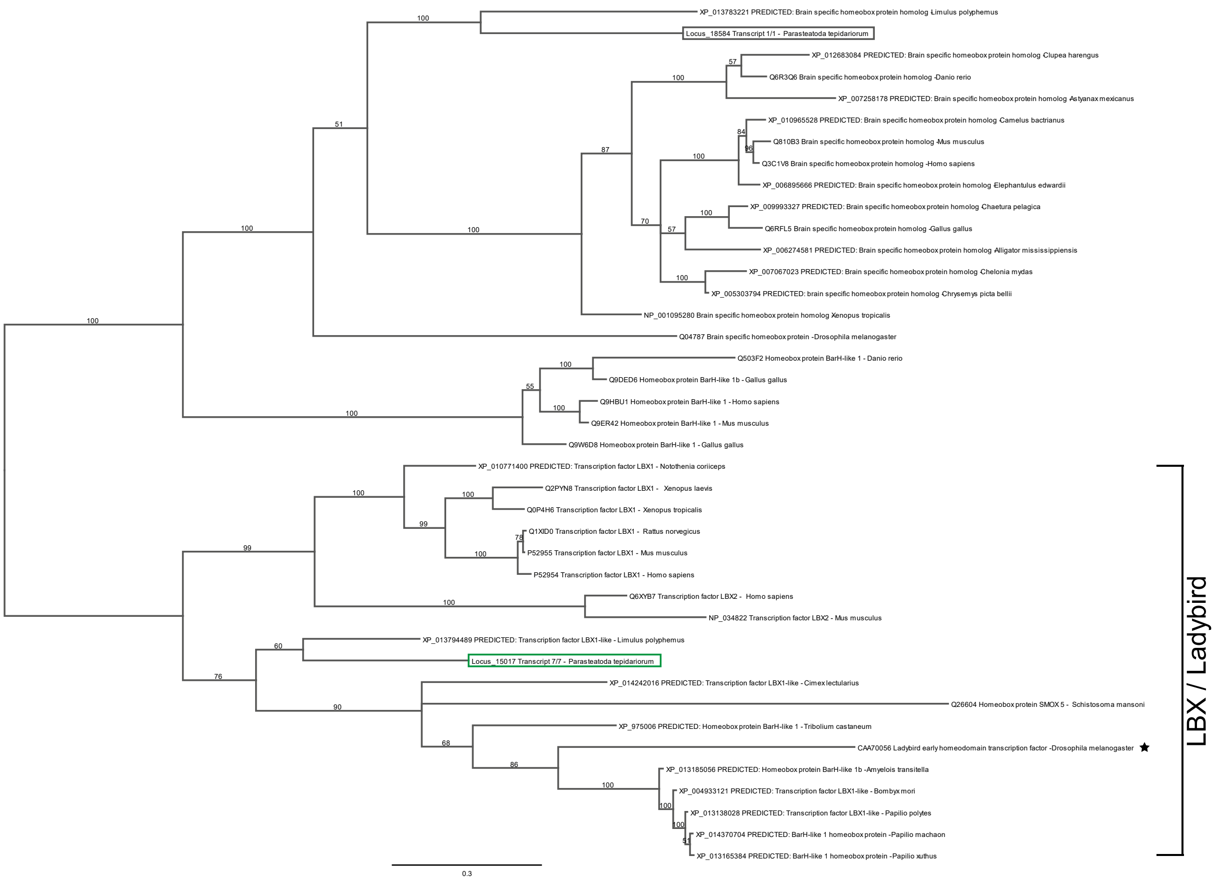

Figure S39. Bayesian phylogenetic tree of lbe. Sequence used for the initial BLAST search is marked with a star, Homologous *Parasteatoda* sequences marked with green box, other, non-homologous *Parasteatoda* sequences marked with grey box. Branch labels indicate posterior probability as determined by MrBayes.

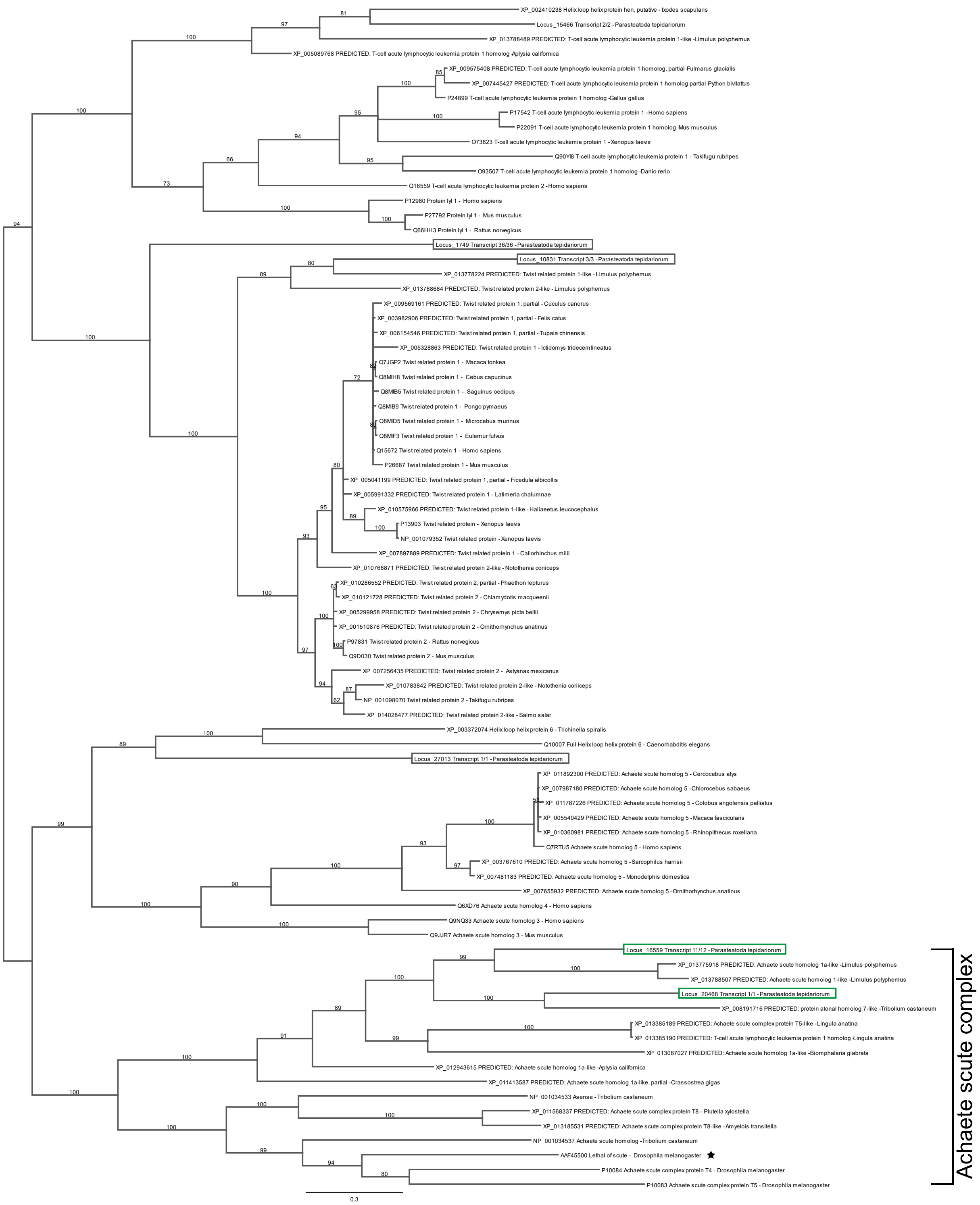

Figure S40. Bayesian phylogenetic tree of l(1)sc. Sequence used for the initial BLAST search is marked with a star, Homologous *Parasteatoda* sequences marked with green box, other, non-homologous *Parasteatoda* sequences marked with grey box. Branch labels indicate posterior probability as determined by MrBayes.

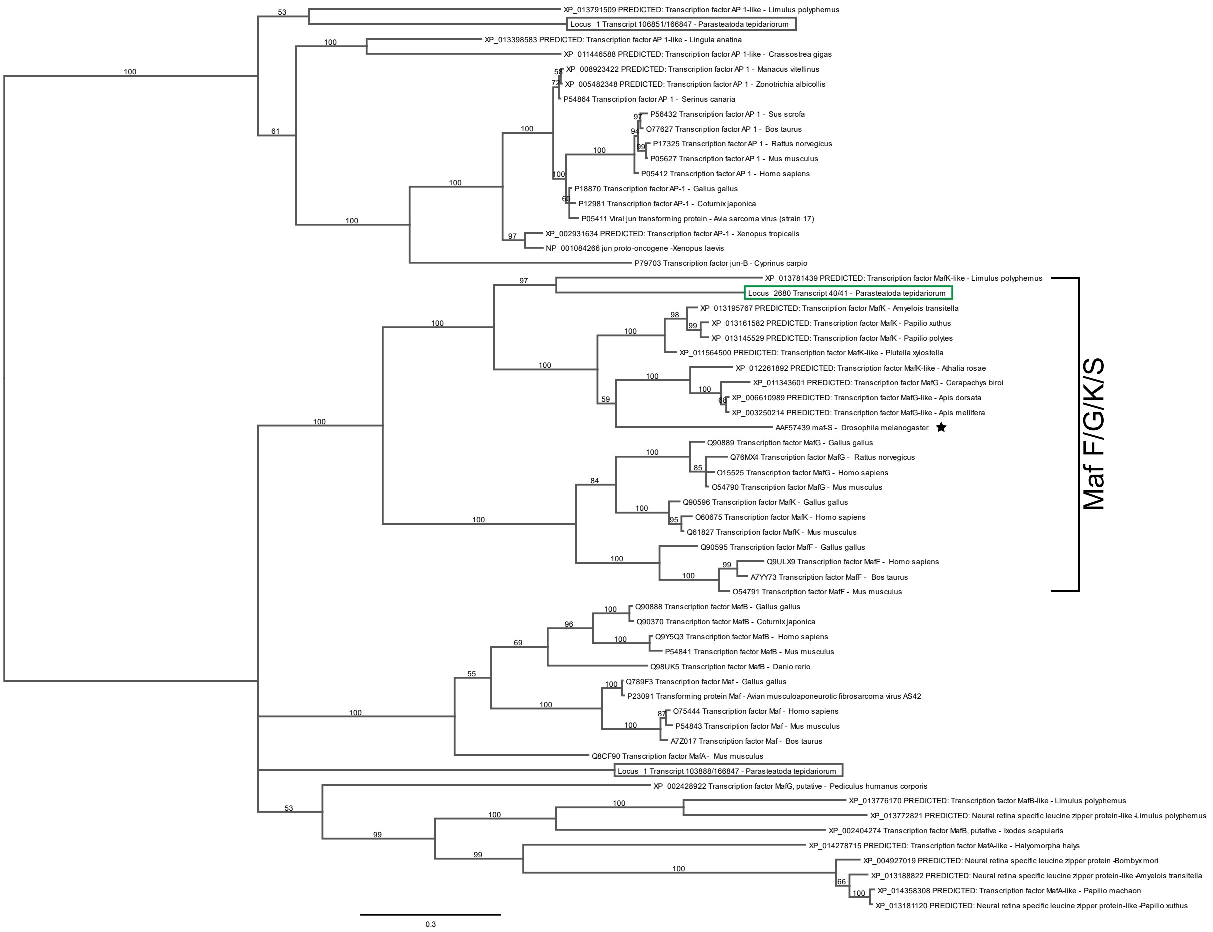

Figure S41. Bayesian phylogenetic tree of maf-S. Sequence used for the initial BLAST search is marked with a star, Homologous *Parasteatoda* sequences marked with green box, other, non-homologous *Parasteatoda* sequences marked with grey box. Branch labels indicate posterior probability as determined by MrBayes.

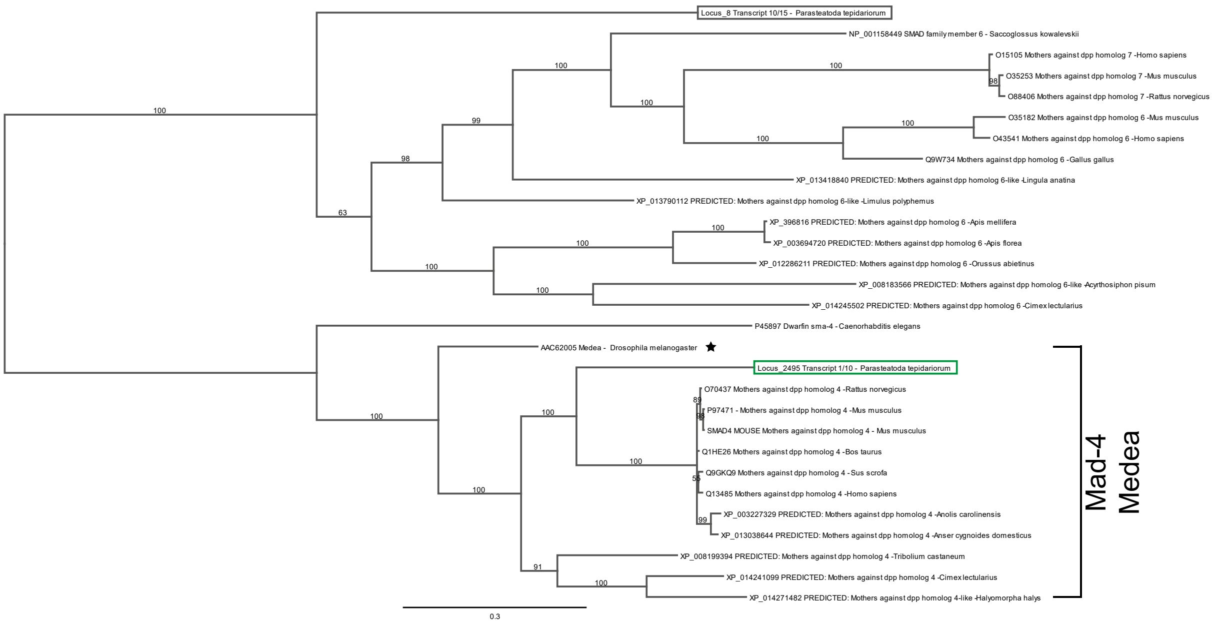

Figure S42. Bayesian phylogenetic tree of Med. Sequence used for the initial BLAST search is marked with a star, Homologous *Parasteatoda* sequences marked with green box, other, non-homologous *Parasteatoda* sequences marked with grey box. Branch labels indicate posterior probability as determined by MrBayes.

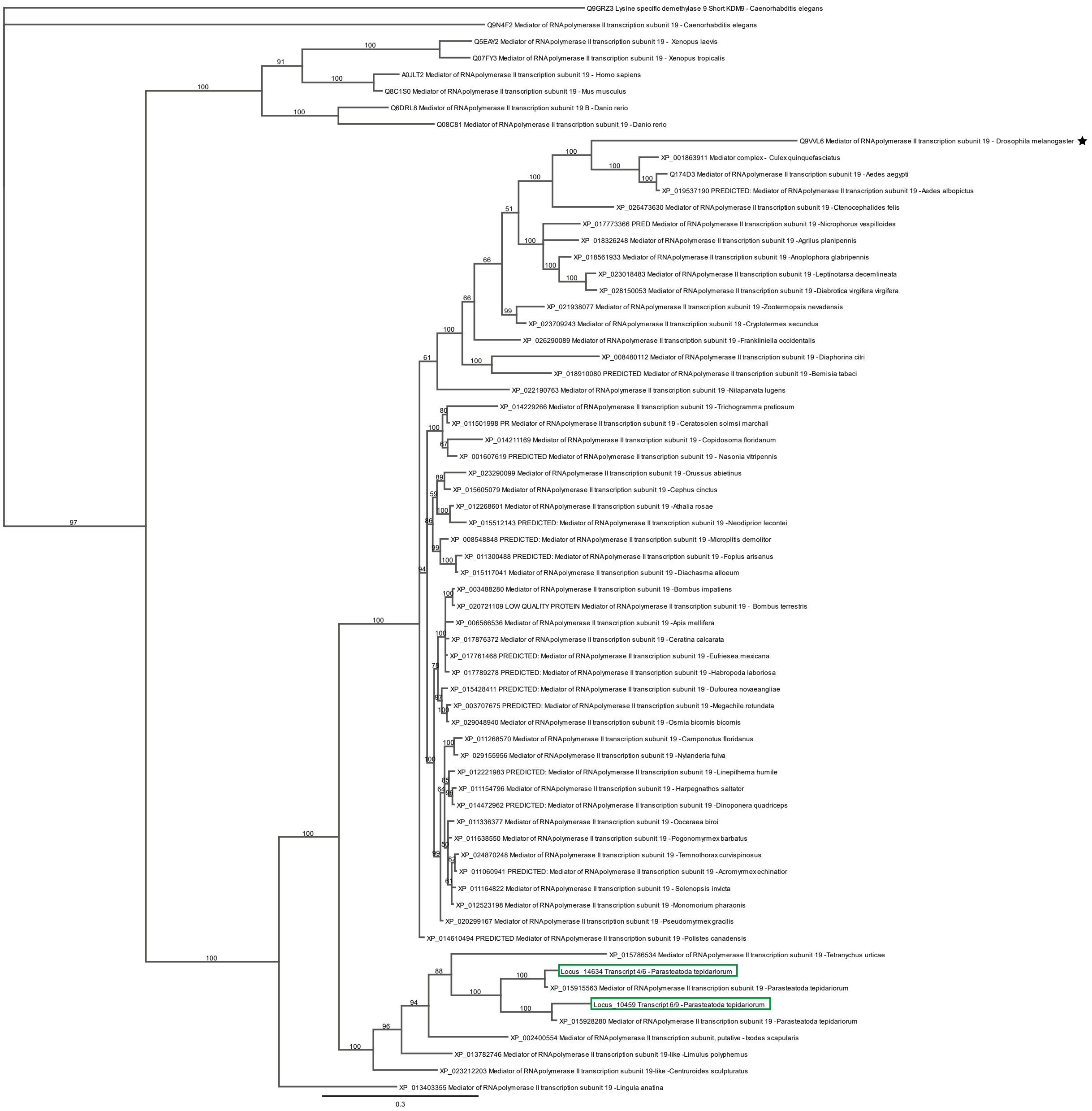

Figure S43. Bayesian phylogenetic tree of MED19. Sequence used for the initial BLAST search is marked with a star, Homologous *Parasteatoda* sequences marked with green box, other, non-homologous *Parasteatoda* sequences marked with grey box. Branch labels indicate posterior probability as determined by MrBayes.

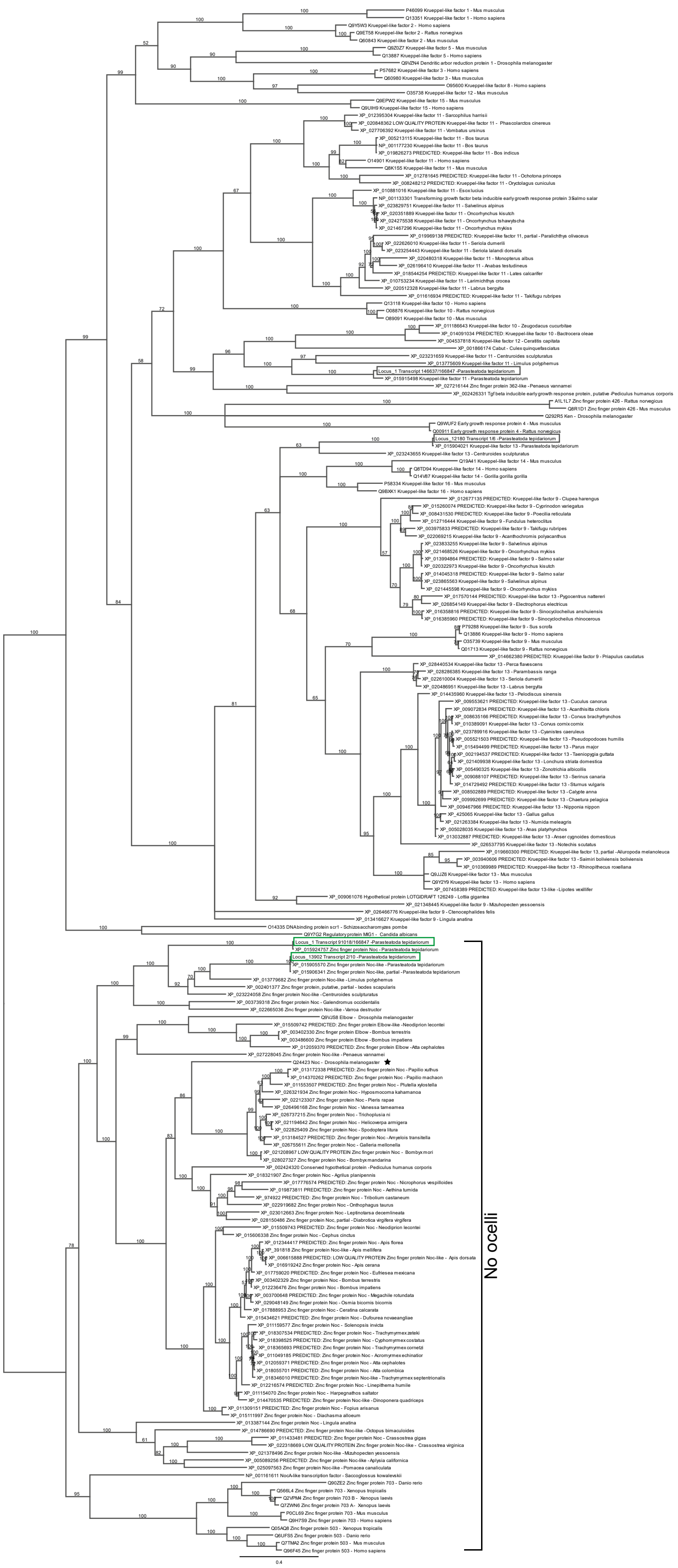

Figure S44. Bayesian phylogenetic tree of noc. Sequence used for the initial BLAST search is marked with a star, Homologous *Parasteatoda* sequences marked with green box, other, non-homologous *Parasteatoda* sequences marked with grey box. Branch labels indicate posterior probability as determined by MrBayes.

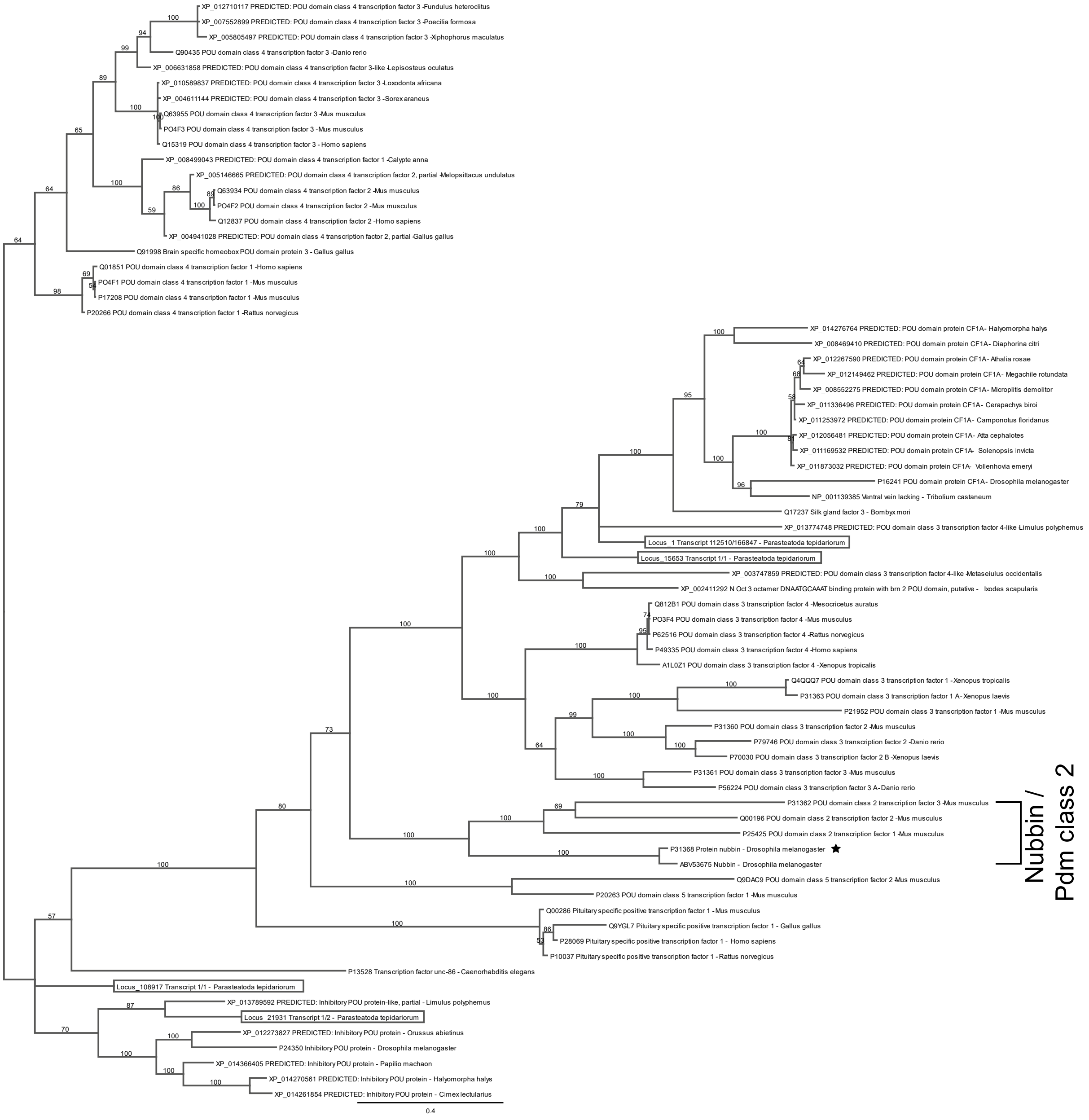

Figure S45. Bayesian phylogenetic tree of nub. Sequence used for the initial BLAST search is marked with a star, Homologous *Parasteatoda* sequences marked with green box, other, non-homologous *Parasteatoda* sequences marked with grey box. Branch labels indicate posterior probability as determined by MrBayes.

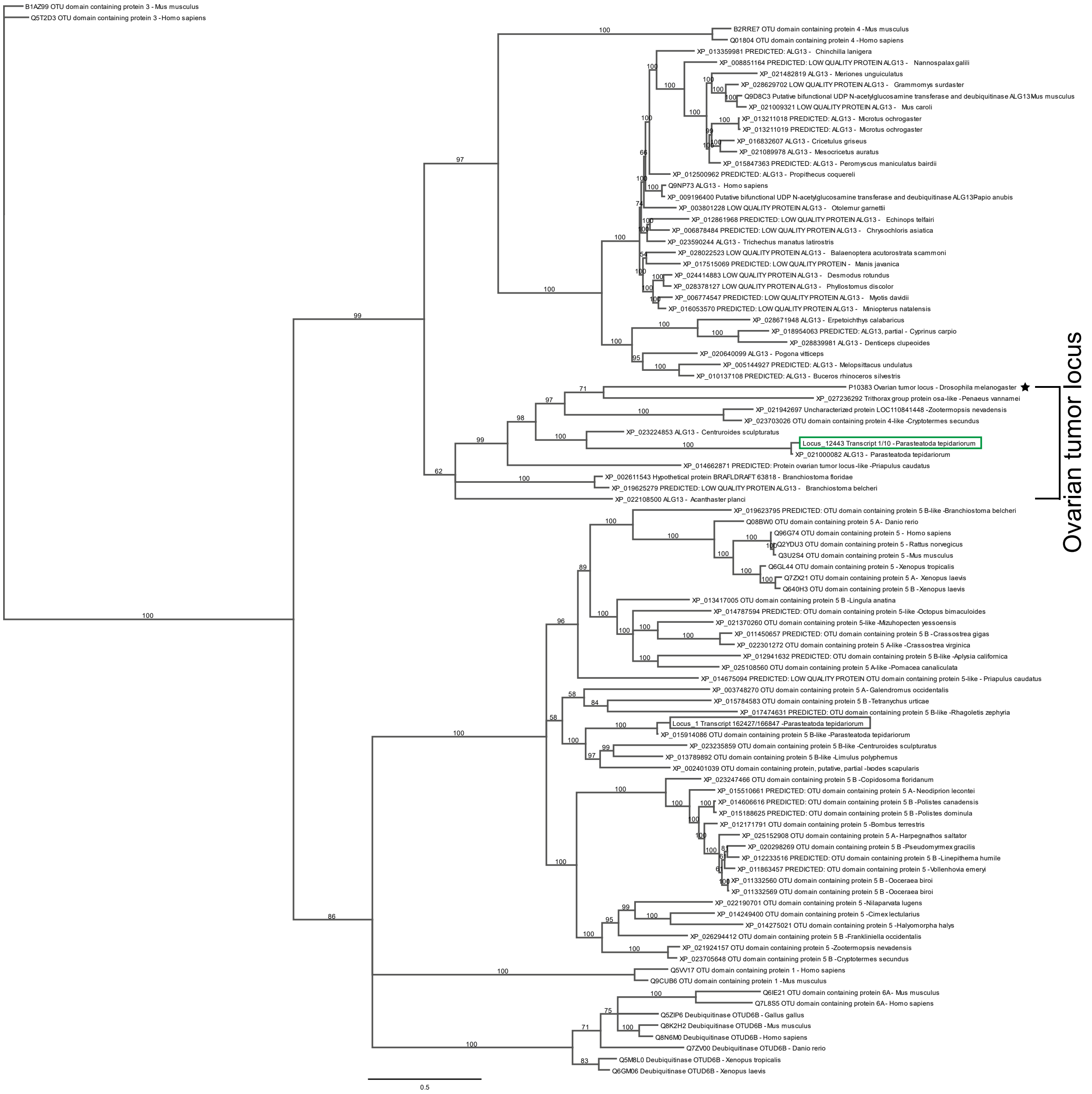

Figure S46. Bayesian phylogenetic tree of otu. Sequence used for the initial BLAST search is marked with a star, Homologous *Parasteatoda* sequences marked with green box, other, non-homologous *Parasteatoda* sequences marked with grey box. Branch labels indicate posterior probability as determined by MrBayes.

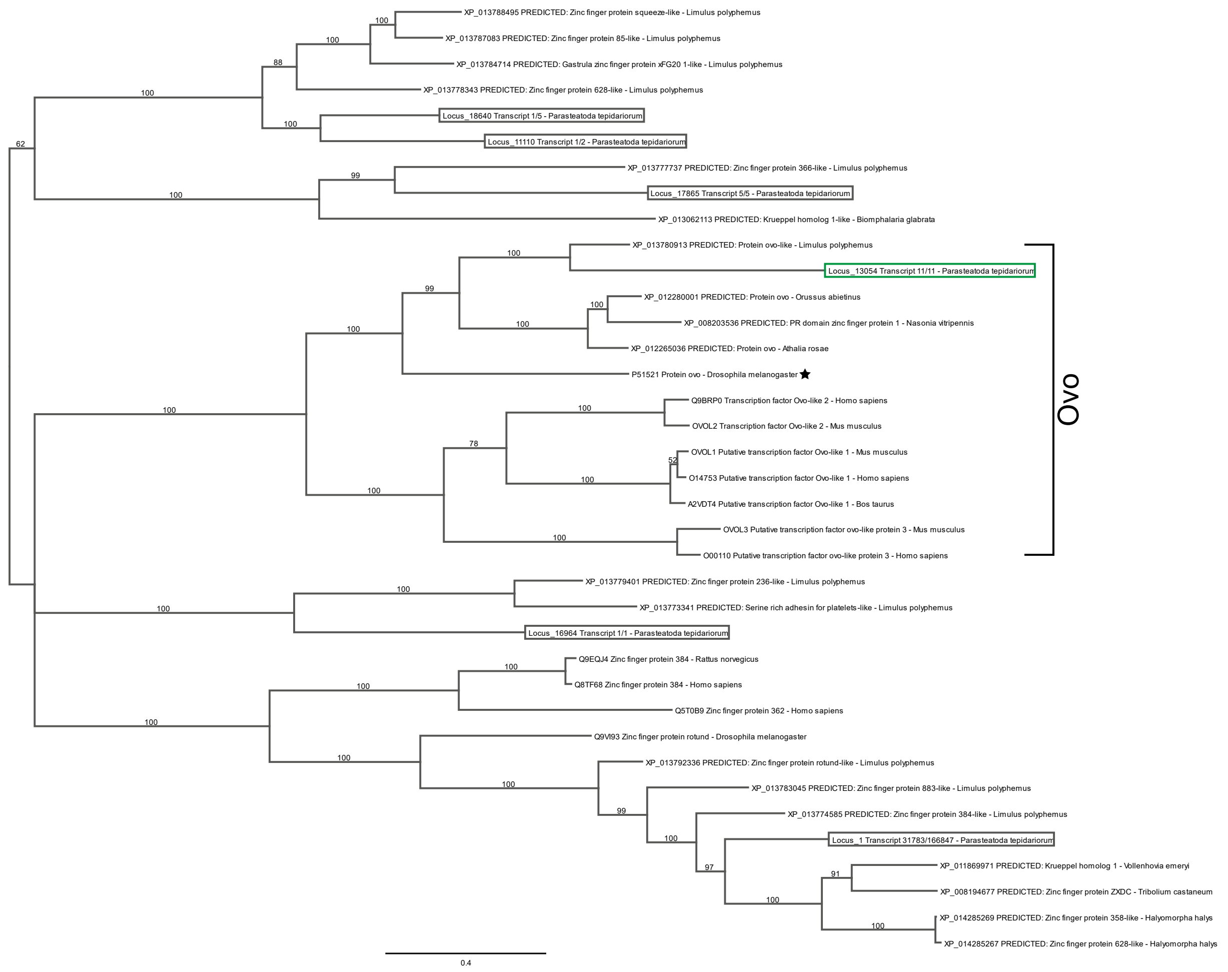

Figure S47. Bayesian phylogenetic tree of ovo. Sequence used for the initial BLAST search is marked with a star, Homologous *Parasteatoda* sequences marked with green box, other, non-homologous *Parasteatoda* sequences marked with grey box. Branch labels indicate posterior probability as determined by MrBayes.

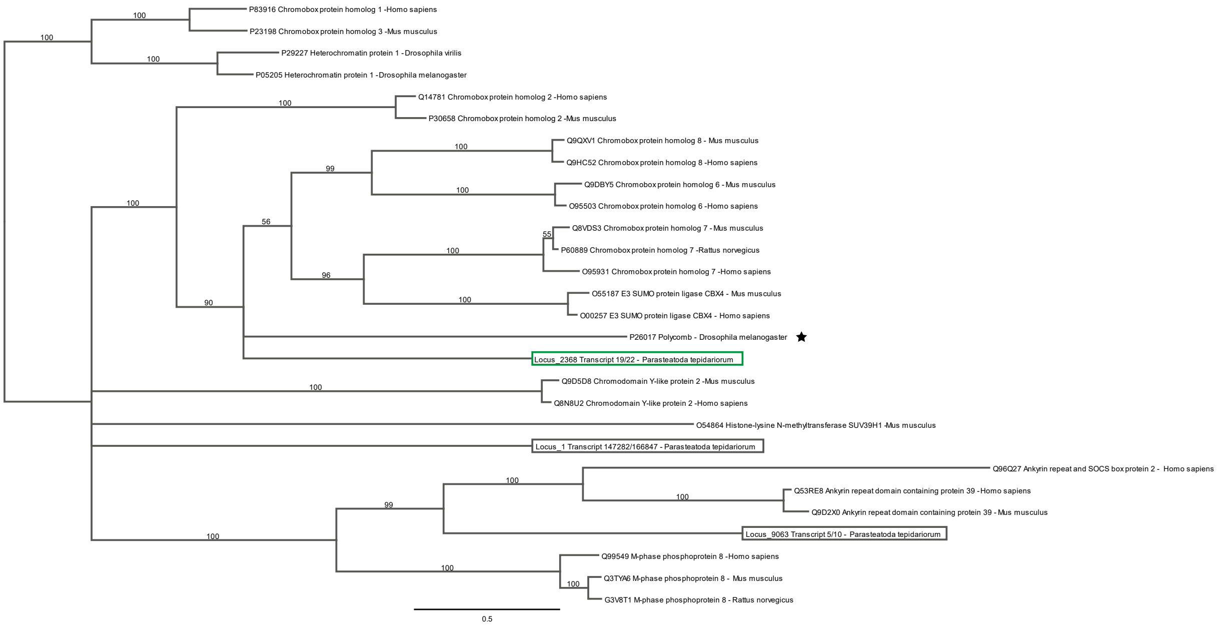

Figure S48. Bayesian phylogenetic tree of Pc. Sequence used for the initial BLAST search is marked with a star, Homologous *Parasteatoda* sequences marked with green box, other, non-homologous *Parasteatoda* sequences marked with grey box. Branch labels indicate posterior probability as determined by MrBayes.

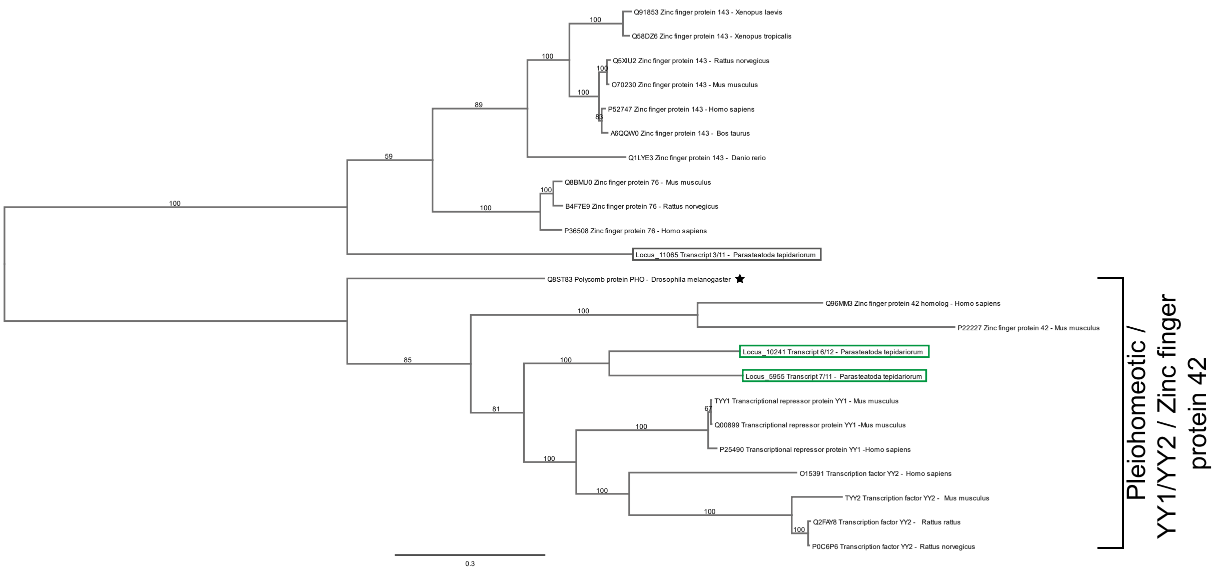

Figure S49. Bayesian phylogenetic tree of pho. Sequence used for the initial BLAST search is marked with a star, Homologous *Parasteatoda* sequences marked with green box, other, non-homologous *Parasteatoda* sequences marked with grey box. Branch labels indicate posterior probability as determined by MrBayes.

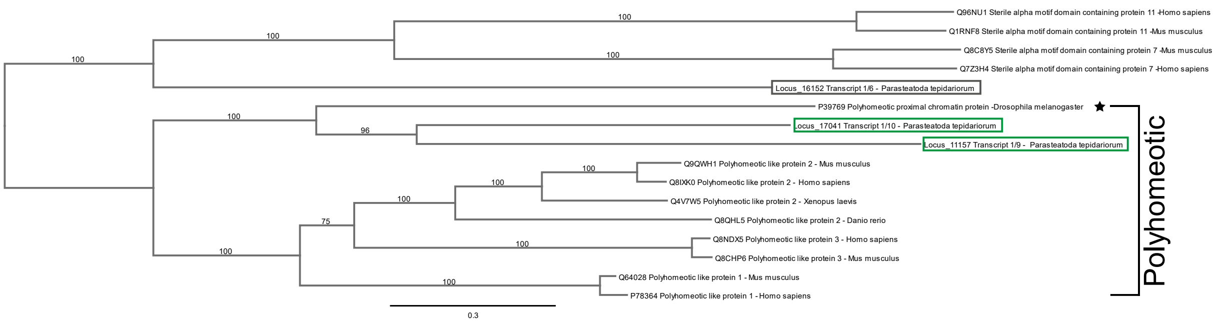

Figure S50. Bayesian phylogenetic tree of ph-p. Sequence used for the initial BLAST search is marked with a star, Homologous *Parasteatoda* sequences marked with green box, other, non-homologous *Parasteatoda* sequences marked with grey box. Branch labels indicate posterior probability as determined by MrBayes.

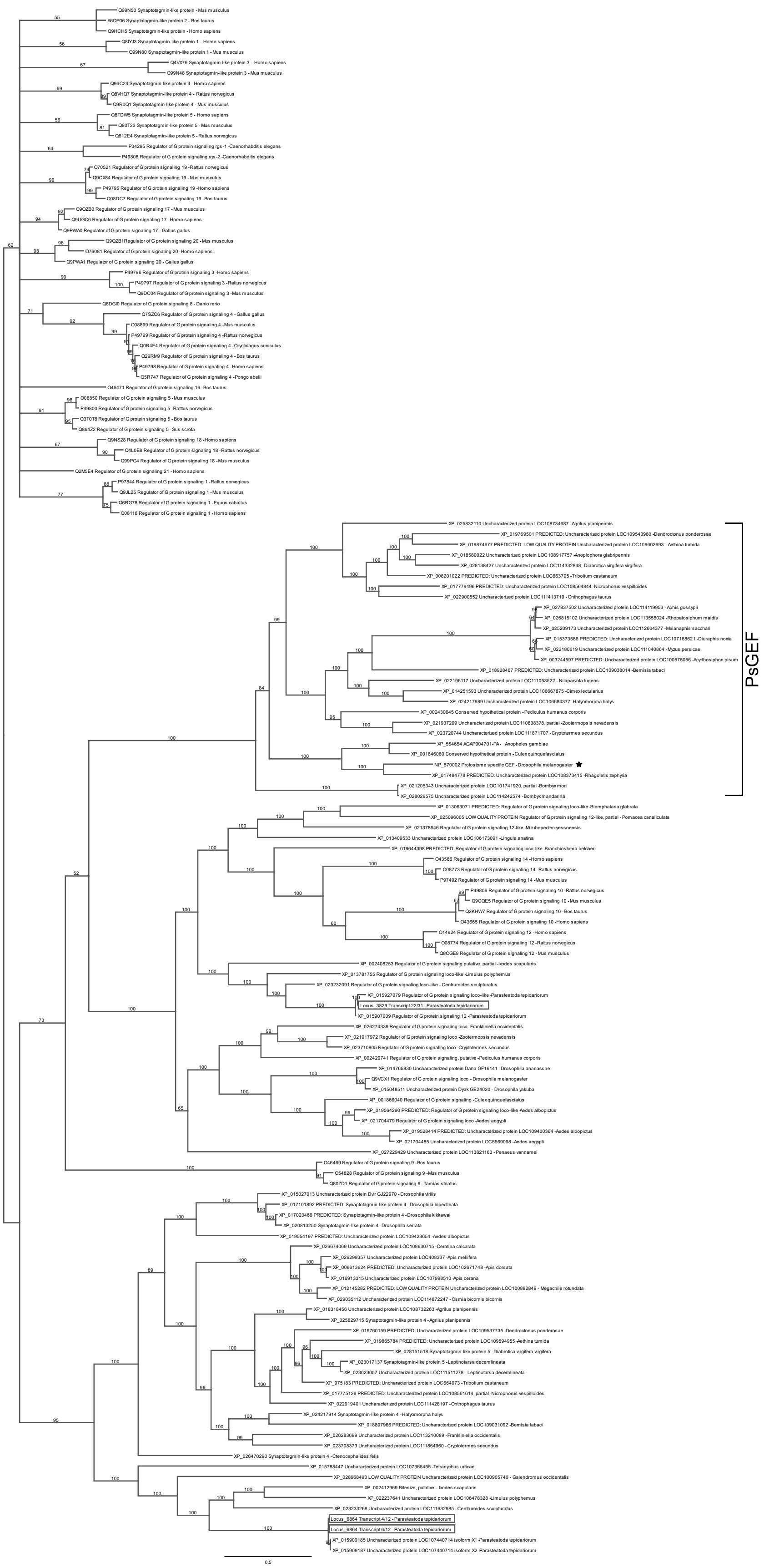

Figure S51. Bayesian phylogenetic tree of PsGEF. Sequence used for the initial BLAST search is marked with a star, Homologous *Parasteatoda* sequences marked with green box, other, non-homologous *Parasteatoda* sequences marked with grey box. Branch labels indicate posterior probability as determined by MrBayes.

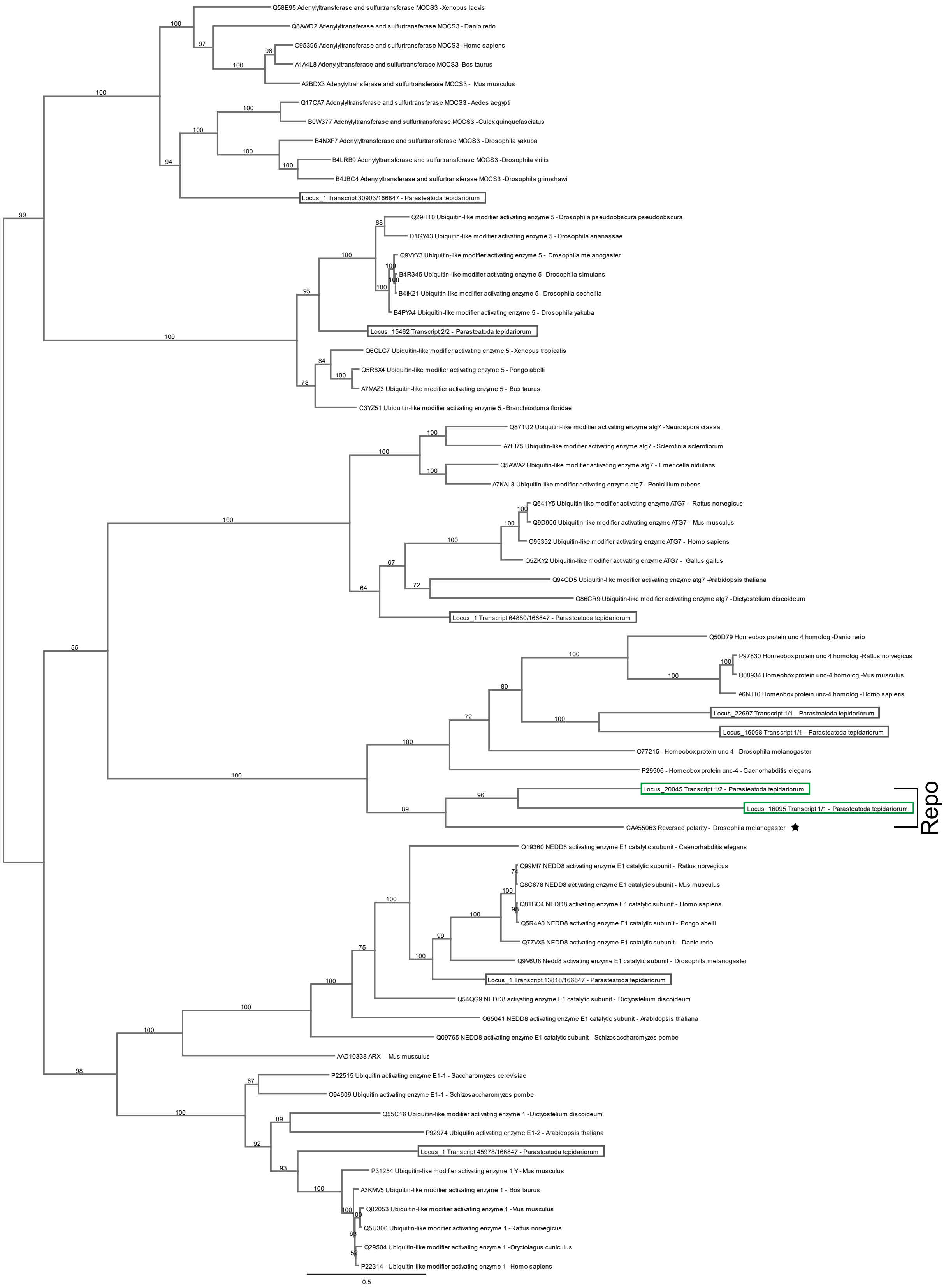

Figure S52. Bayesian phylogenetic tree of repo. Sequence used for the initial BLAST search is marked with a star, Homologous *Parasteatoda* sequences marked with green box, other, non-homologous *Parasteatoda* sequences marked with grey box. Branch labels indicate posterior probability as determined by MrBayes.

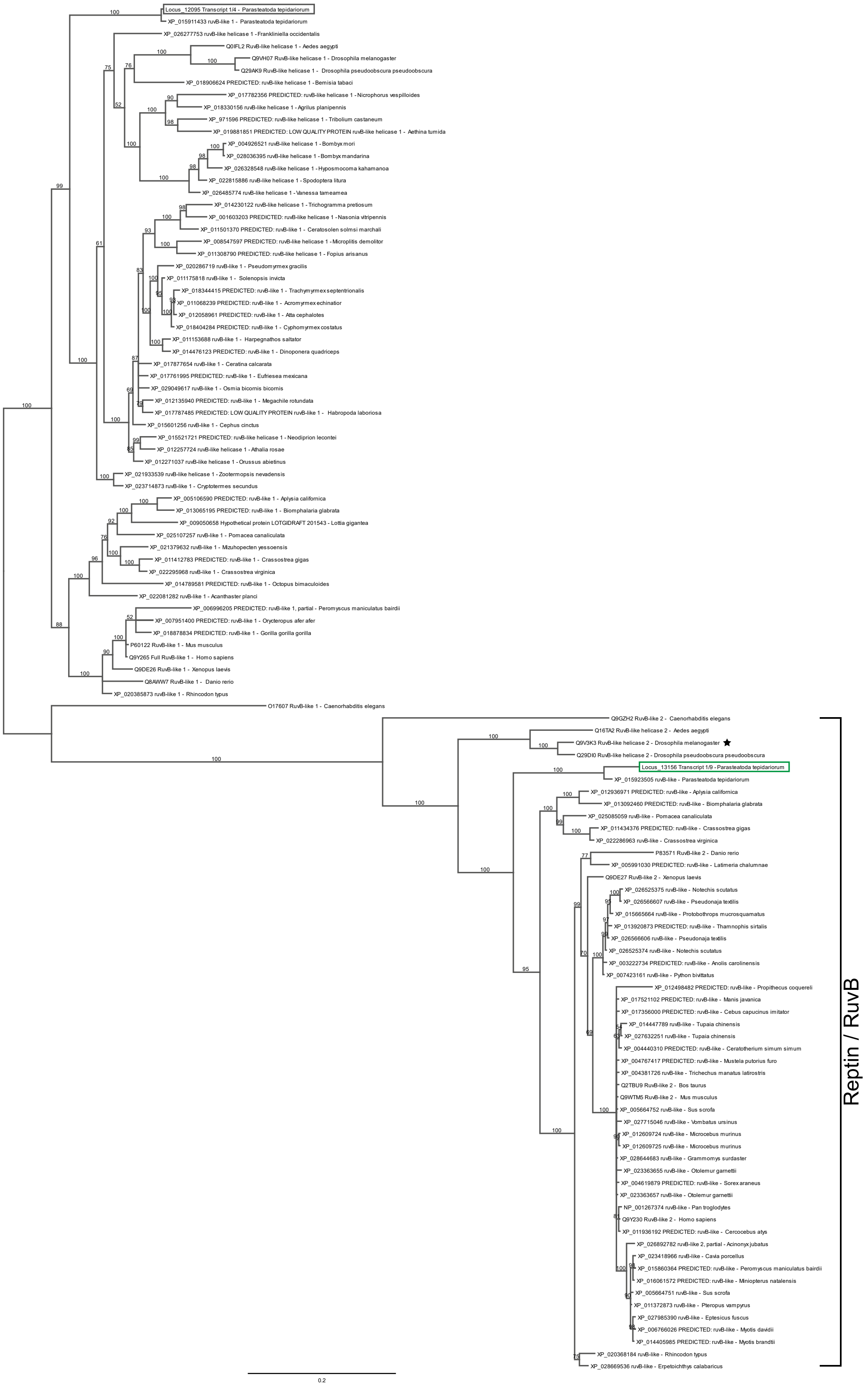

Figure S53. Bayesian phylogenetic tree of rept. Sequence used for the initial BLAST search is marked with a star, Homologous *Parasteatoda* sequences marked with green box, other, non-homologous *Parasteatoda* sequences marked with grey box. Branch labels indicate posterior probability as determined by MrBayes.

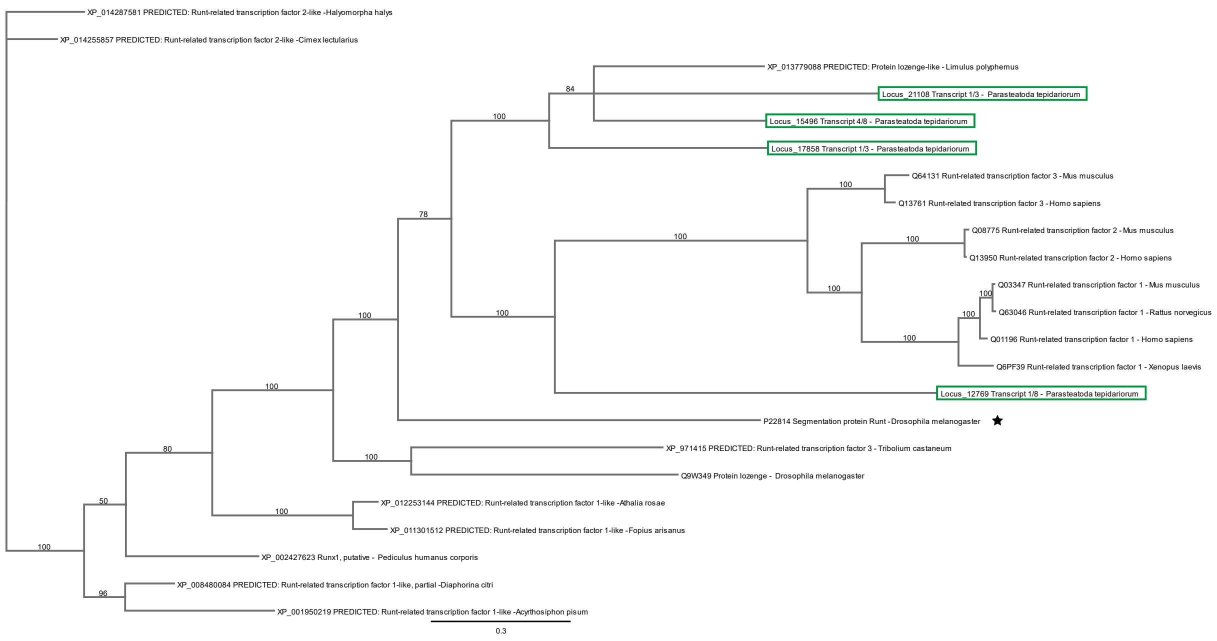

Figure S54. Bayesian phylogenetic tree of run. Sequence used for the initial BLAST search is marked with a star, Homologous *Parasteatoda* sequences marked with green box, other, non-homologous *Parasteatoda* sequences marked with grey box. Branch labels indicate posterior probability as determined by MrBayes.

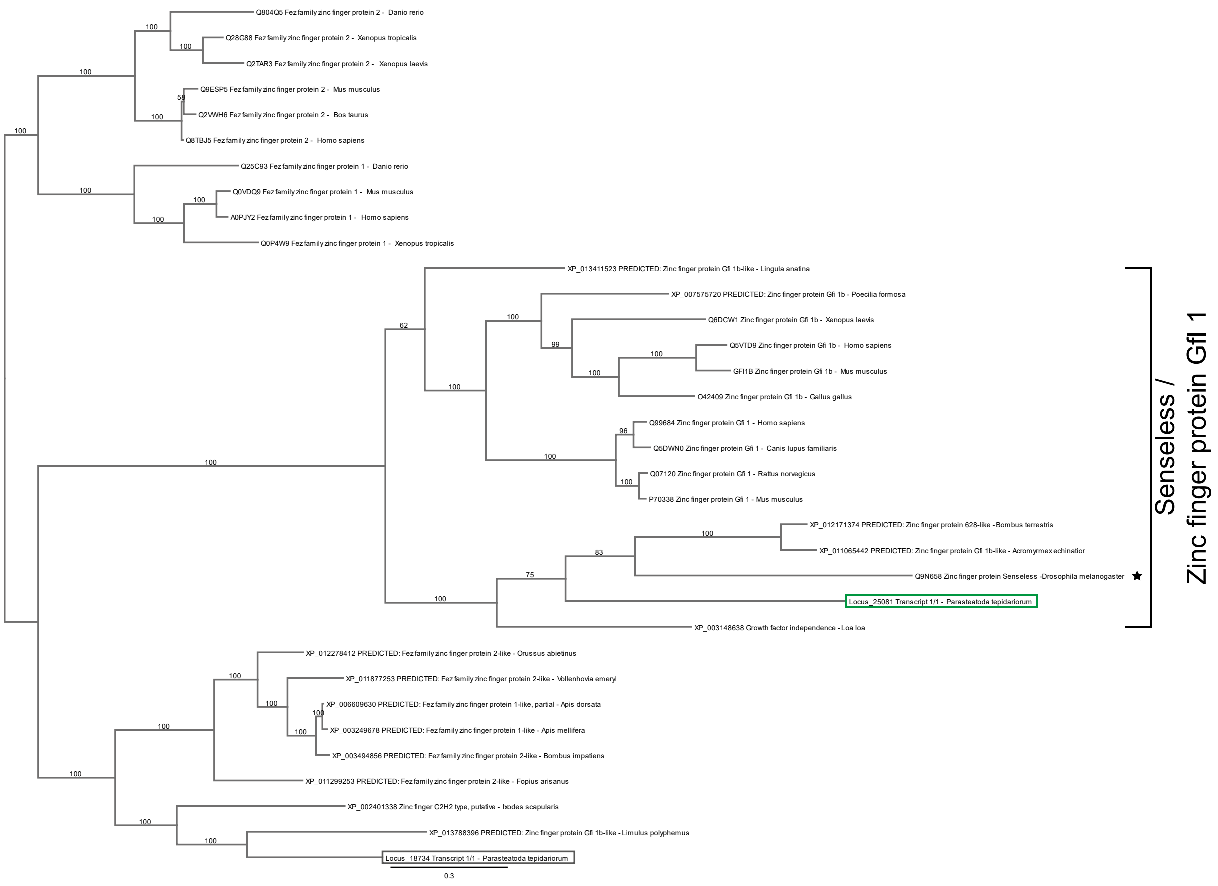

Figure S55. Bayesian phylogenetic tree of sens. Sequence used for the initial BLAST search is marked with a star, Homologous *Parasteatoda* sequences marked with green box, other, non-homologous *Parasteatoda* sequences marked with grey box. Branch labels indicate posterior probability as determined by MrBayes.

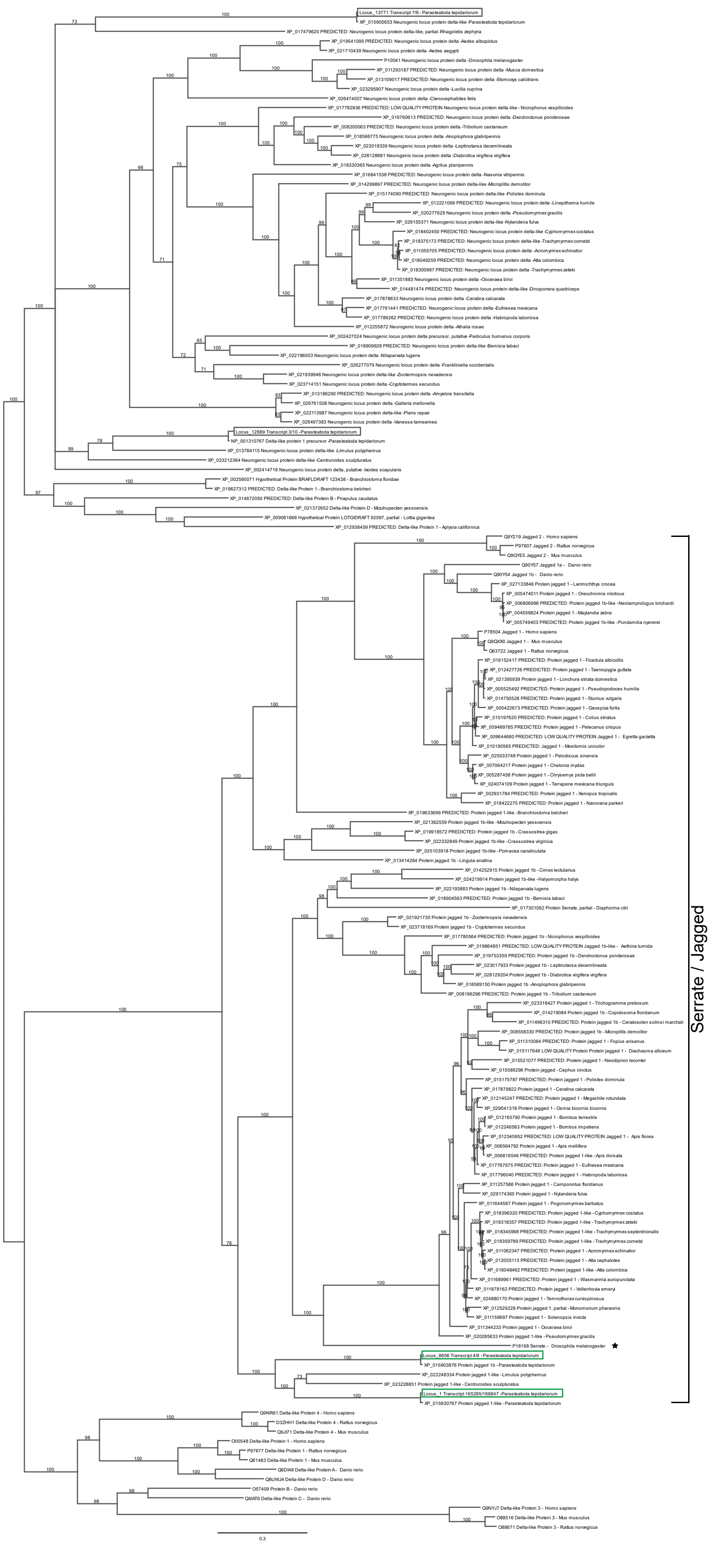

Figure S56. Bayesian phylogenetic tree of Ser. Sequence used for the initial BLAST search is marked with a star, Homologous *Parasteatoda* sequences marked with green box, other, non-homologous *Parasteatoda* sequences marked with grey box. Branch labels indicate posterior probability as determined by MrBayes.

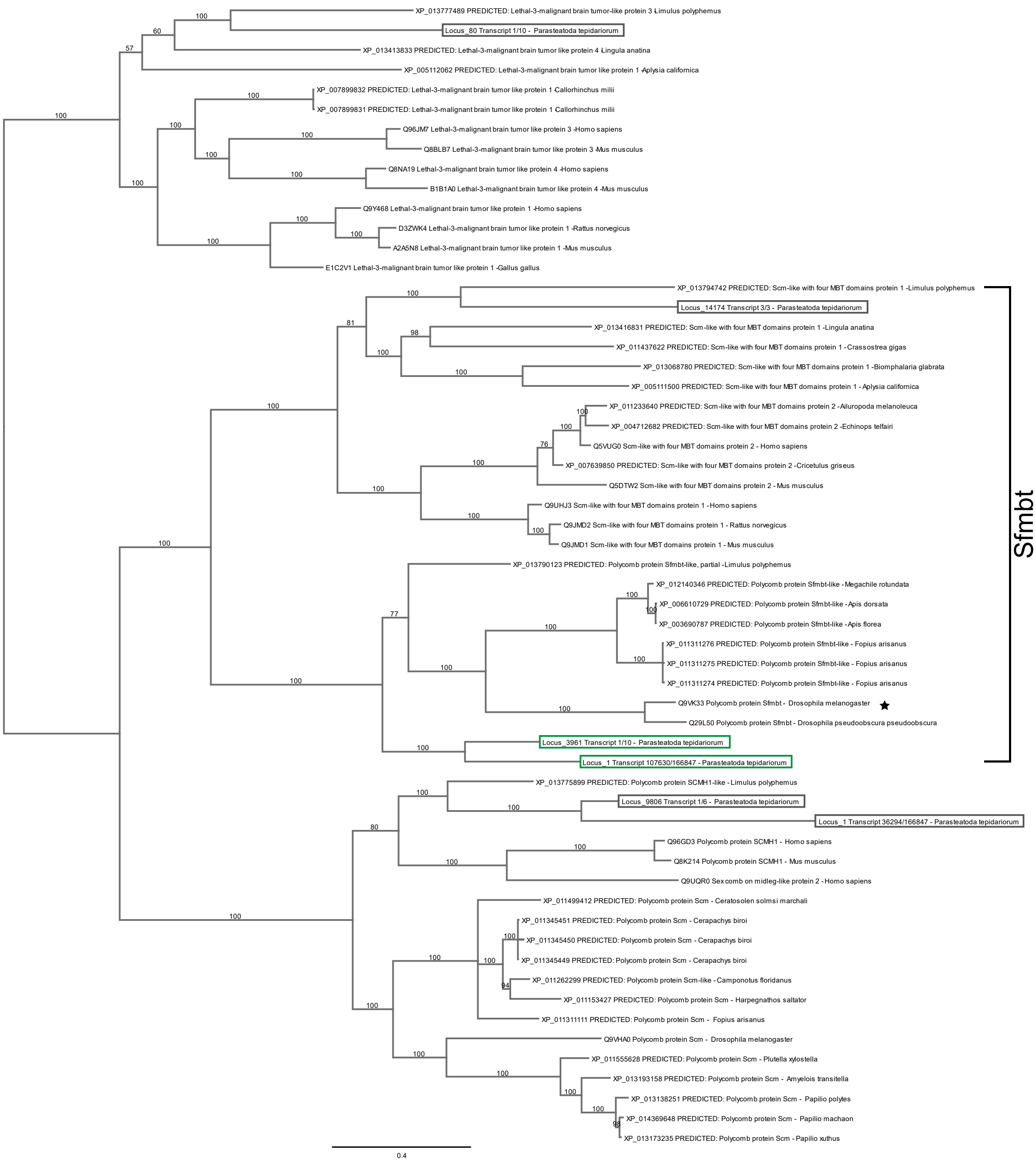

Figure S57. Bayesian phylogenetic tree of Sfmbt. Sequence used for the initial BLAST search is marked with a star, Homologous *Parasteatoda* sequences marked with green box, other, non-homologous *Parasteatoda* sequences marked with grey box. Branch labels indicate posterior probability as determined by MrBayes.

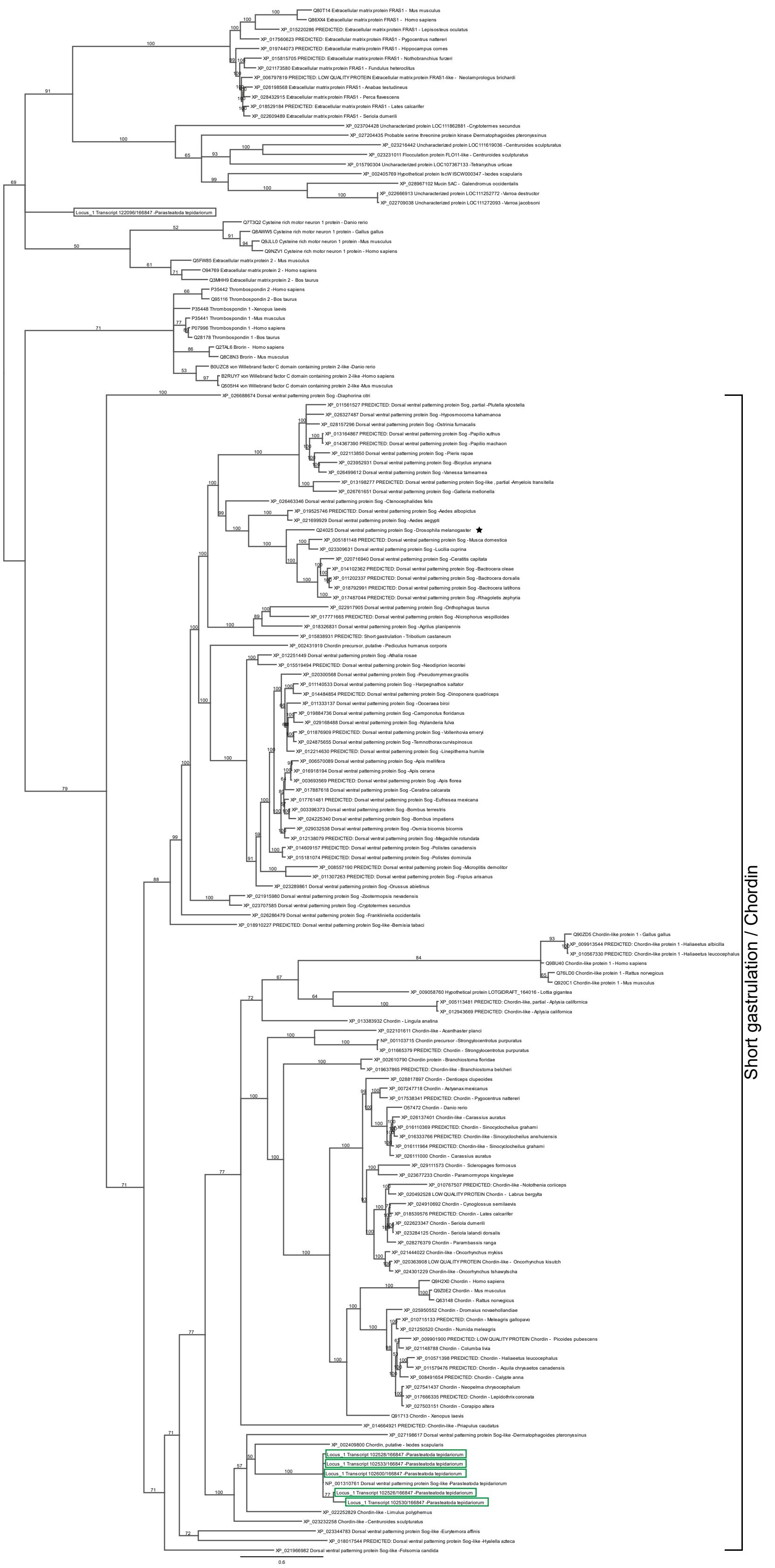

Figure S58. Bayesian phylogenetic tree of sog. Sequence used for the initial BLAST search is marked with a star, Homologous *Parasteatoda* sequences marked with green box, other, non-homologous *Parasteatoda* sequences marked with grey box. Branch labels indicate posterior probability as determined by MrBayes.

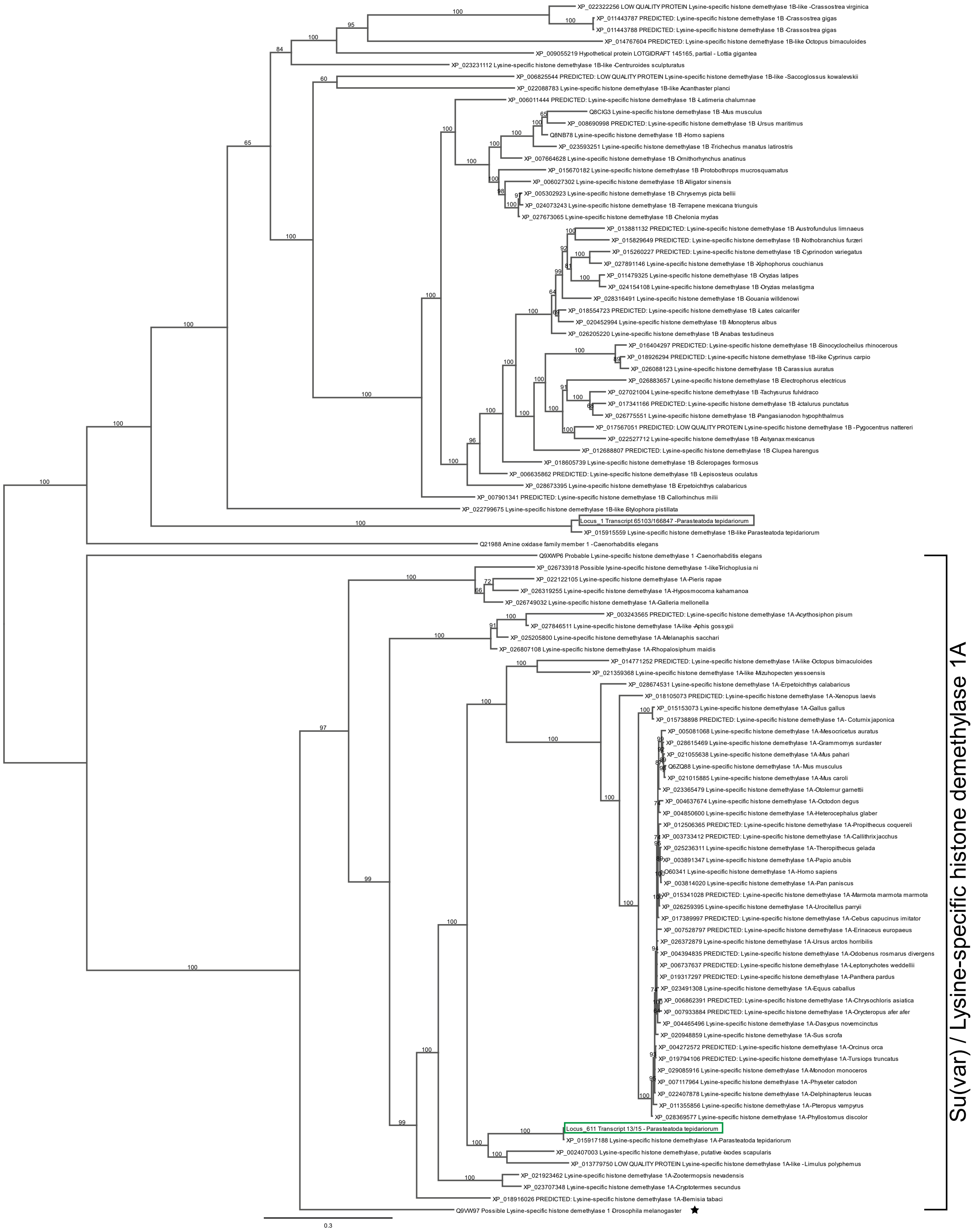

Figure S59. Bayesian phylogenetic tree of Su(var)3-3. Sequence used for the initial BLAST search is marked with a star, Homologous *Parasteatoda* sequences marked with green box, other, non-homologous *Parasteatoda* sequences marked with grey box. Branch labels indicate posterior probability as determined by MrBayes.

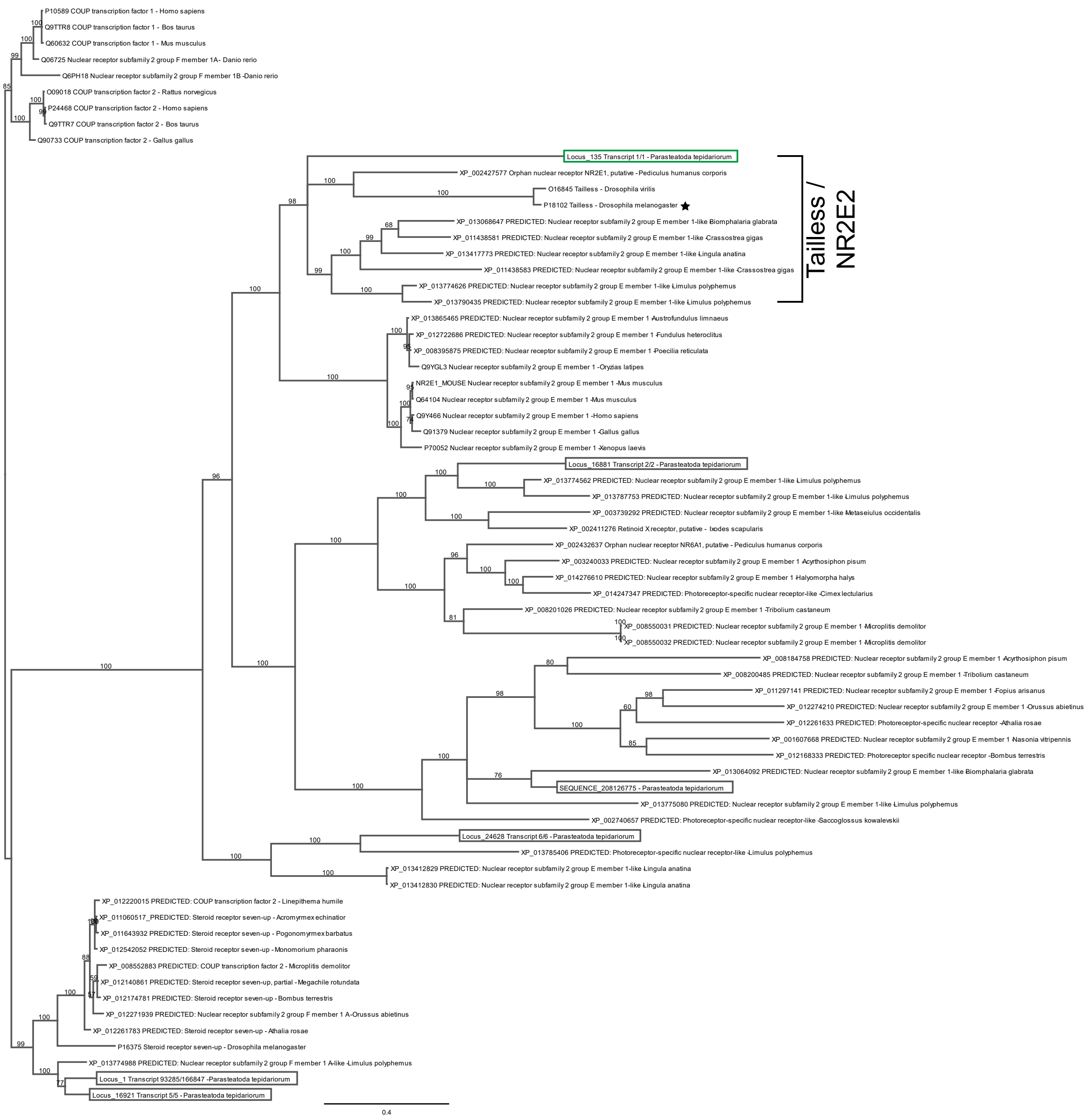

Figure S60. Bayesian phylogenetic tree of tll. Sequence used for the initial BLAST search is marked with a star, Homologous *Parasteatoda* sequences marked with green box, other, non-homologous *Parasteatoda* sequences marked with grey box. Branch labels indicate posterior probability as determined by MrBayes.

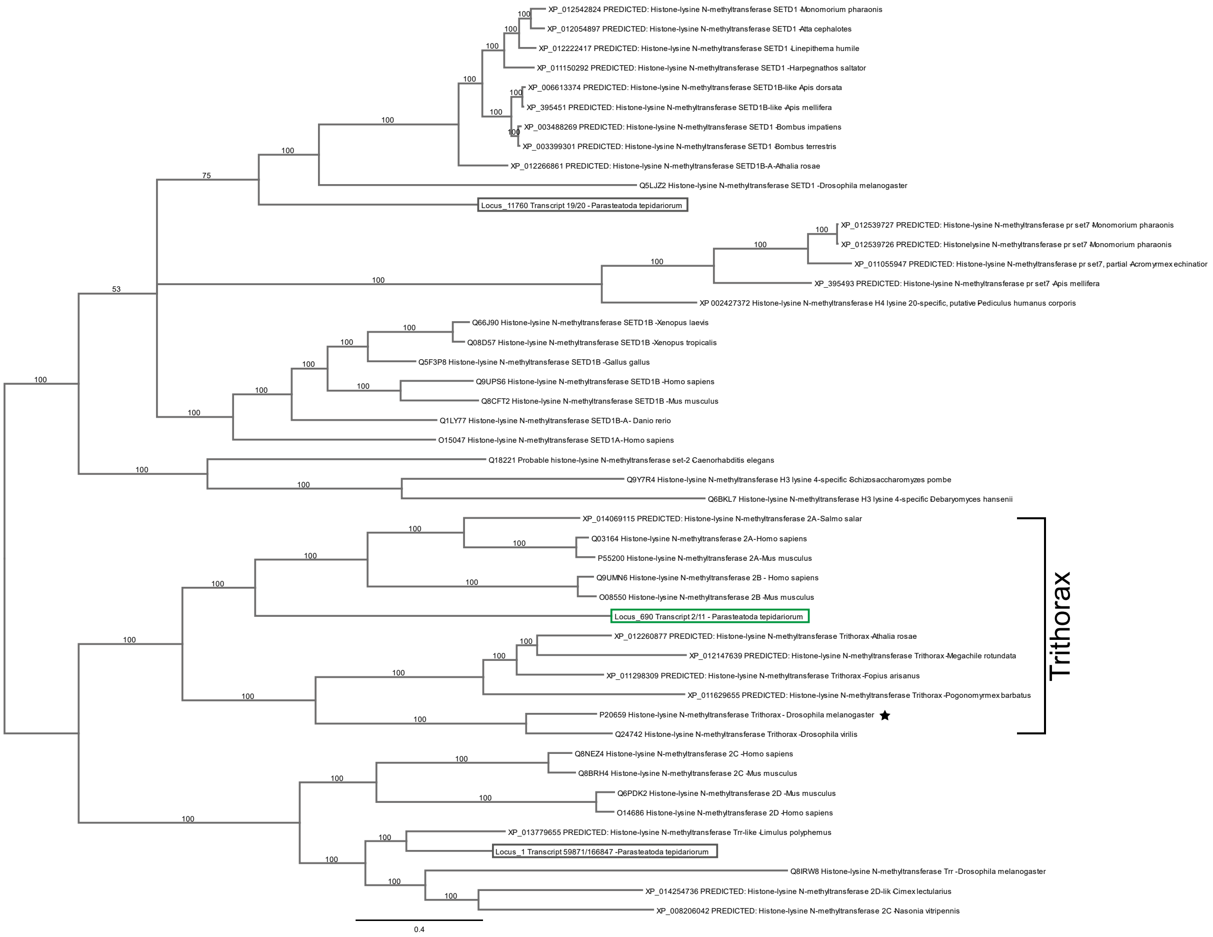

Figure S61. Bayesian phylogenetic tree of trx. Sequence used for the initial BLAST search is marked with a star, Homologous *Parasteatoda* sequences marked with green box, other, non-homologous *Parasteatoda* sequences marked with grey box. Branch labels indicate posterior probability as determined by MrBayes.

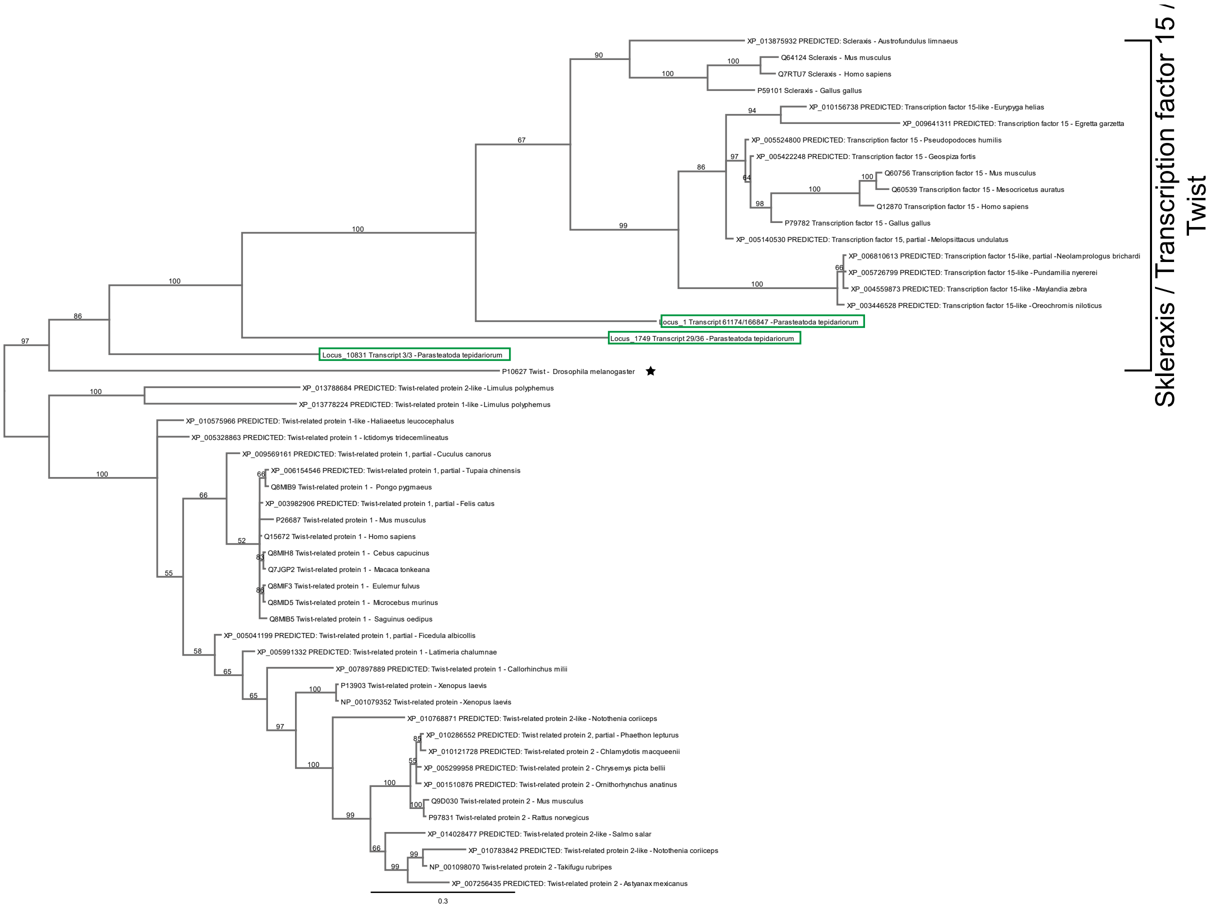

Figure S62. Bayesian phylogenetic tree of twi. Sequence used for the initial BLAST search is marked with a star, Homologous *Parasteatoda* sequences marked with green box, other, non-homologous *Parasteatoda* sequences marked with grey box. Branch labels indicate posterior probability as determined by MrBayes.

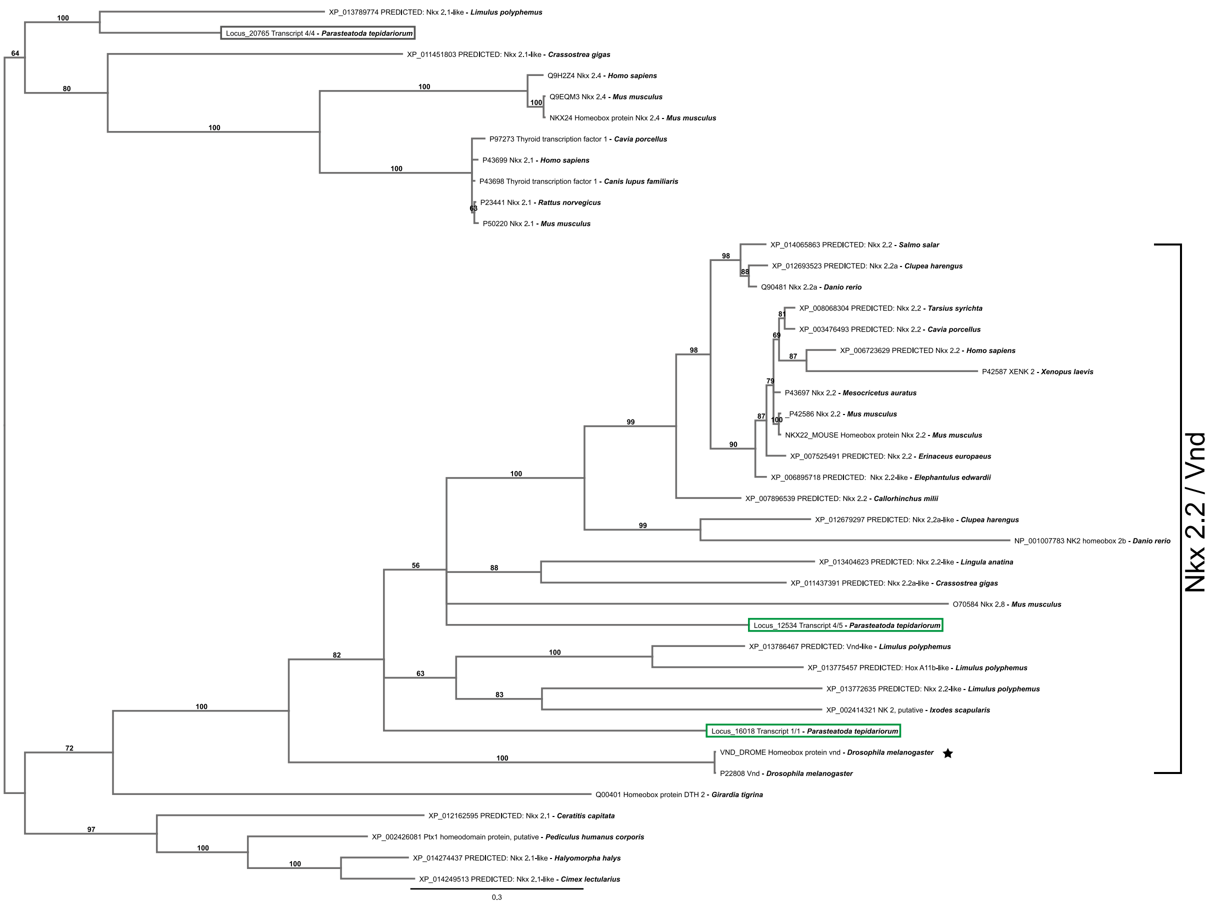

Figure S63. Bayesian phylogenetic tree of vnd. Sequence used for the initial BLAST search is marked with a star, Homologous *Parasteatoda* sequences marked with green box, other, non-homologous *Parasteatoda* sequences marked with grey box. Branch labels indicate posterior probability as determined by MrBayes.

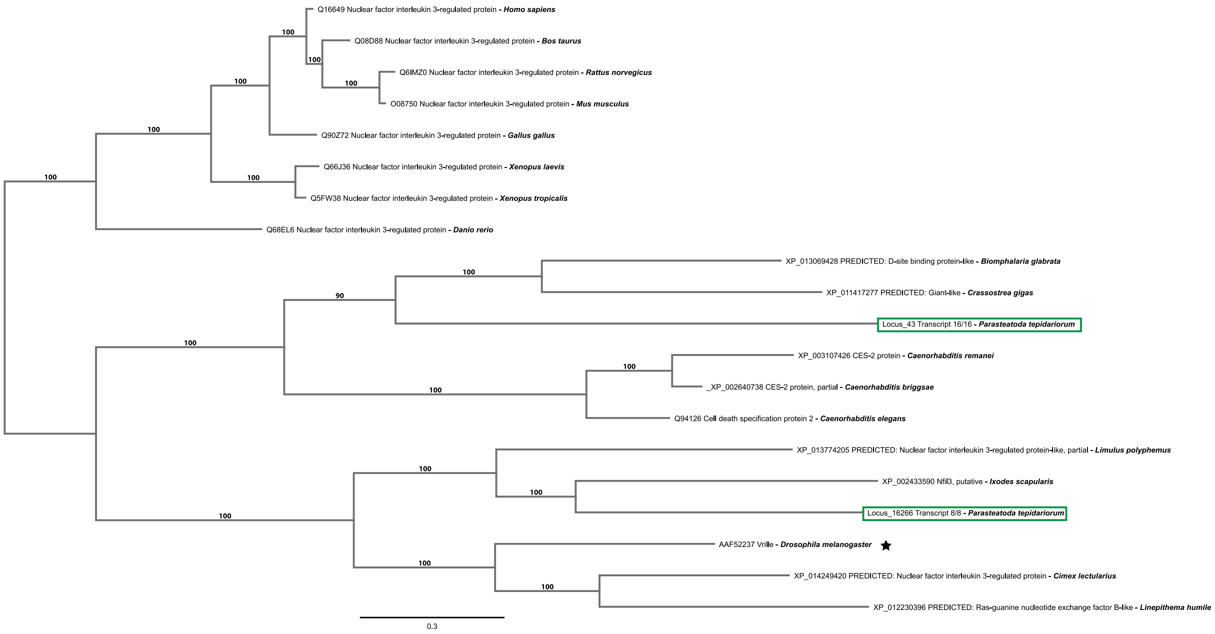

Figure S64. Bayesian phylogenetic tree of vri. Sequence used for the initial BLAST search is marked with a star, Homologous *Parasteatoda* sequences marked with green box, other, non-homologous *Parasteatoda* sequences marked with grey box. Branch labels indicate posterior probability as determined by MrBayes.

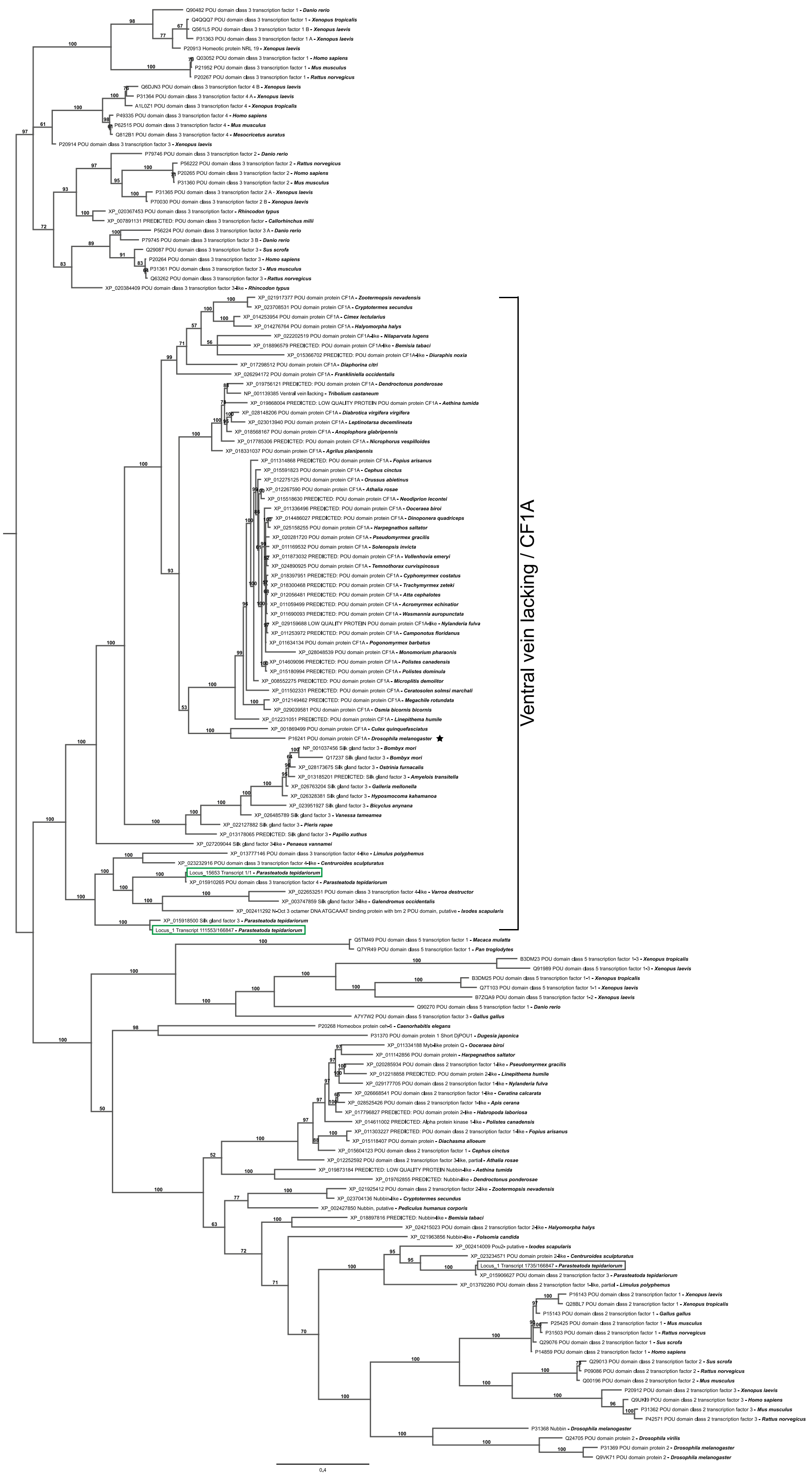

Figure S65. Bayesian phylogenetic tree of vvl. Sequence used for the initial BLAST search is marked with a star, Homologous *Parasteatoda* sequences marked with green box, other, non-homologous *Parasteatoda* sequences marked with grey box. Branch labels indicate posterior probability as determined by MrBayes.

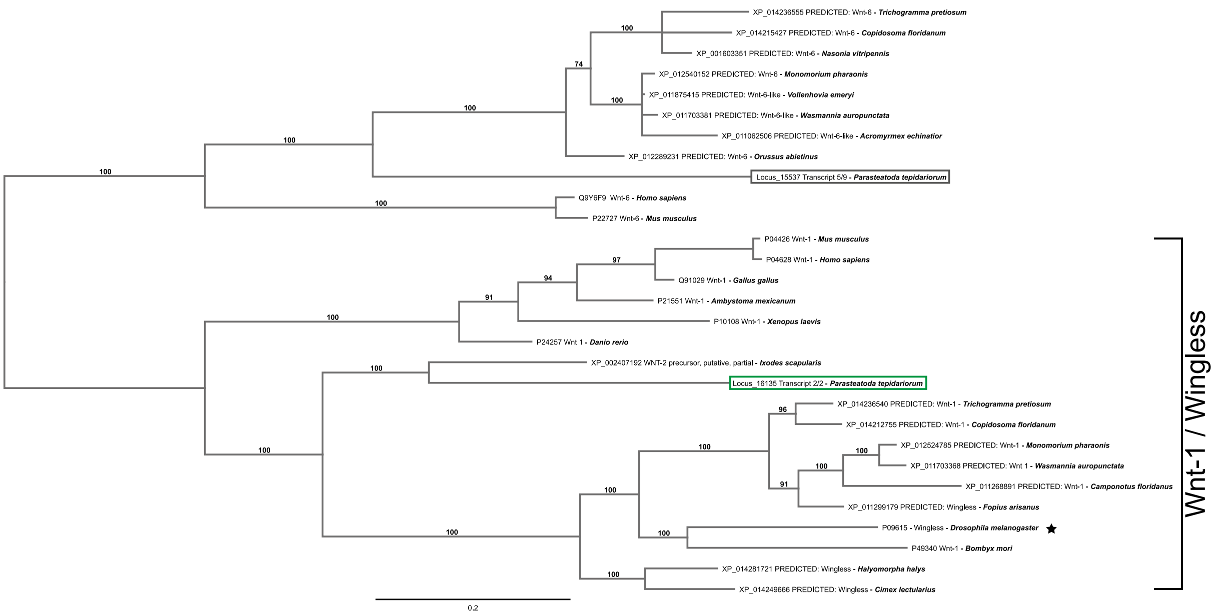

Figure S66. Bayesian phylogenetic tree of wg. Sequence used for the initial BLAST search is marked with a star, Homologous *Parasteatoda* sequences marked with green box, other, non-homologous *Parasteatoda* sequences marked with grey box. Branch labels indicate posterior probability as determined by MrBayes.
